# Energy paradox in REM sleep: balancing supply and consumption in brain metabolism

**DOI:** 10.1038/s42003-026-10646-6

**Published:** 2026-07-27

**Authors:** Yusuke Takahashi, Yoko Ikoma, Ko Matsui

**Affiliations:** 1 Super-network Brain Physiology, Graduate School of Life Sciences, Tohoku University, Sendai, Japan; 2 Super-network Brain Physiology, Graduate School of Medicine, Tohoku University, Sendai, Japan

**Keywords:** Metabolism, Neurophysiology, Neuro-vascular interactions, Astrocyte, REM sleep

## Abstract

The brain’s capacity for information processing depends on precisely regulated energy dynamics. Yet how metabolic supply adapts to shifting computational demands across brain states remains unclear. Using wide-field fluorescence imaging through the intact skull of live mice, we simultaneously monitored brain blood volume (BBV), astrocytic pyruvate, and neuronal ATP levels during natural sleep. We found that large-scale metabolic dynamics are coupled to neuronal activity but reorganize in a state-dependent manner. During non-rapid eye movement (NREM) sleep, theta-band electrocorticogram (ECoG) activity predicted subsequent blood volume changes, accompanied by rapid anterior-to-posterior vascular waves. In contrast, REM sleep was marked by a pronounced increase in BBV, originating in the posterior cortex and slowly propagating across the brain. This was accompanied by elevated astrocytic pyruvate; paradoxically, however, neuronal ATP levels declined sharply. These findings reveal a dynamic interplay among neurons, astrocytes, and the vasculature, suggesting that distinct energy-allocation strategies underlie the brain’s computational flexibility.

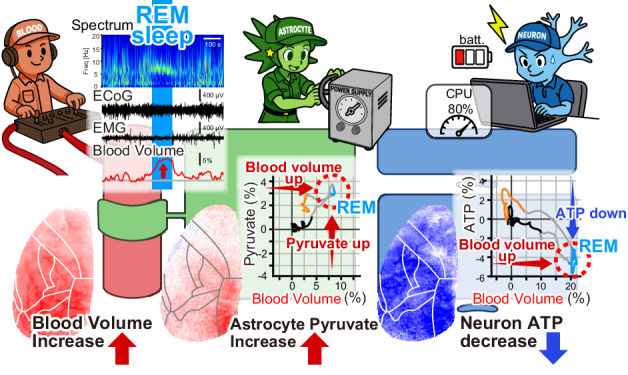

## Abstract (web summary)

Wide-field imaging in mice resolves state-dependent cortical blood-volume dynamics and reveals a REM-sleep paradox, where blood volume and astrocytic pyruvate rise while neuronal ATP falls, uncovering a dissociation between supply and consumption.

## Introduction

Energy is essential for driving information processing^1^. In the brain, interwoven information-processing and metabolic networks finely regulate energy supply and consumption to meet computational demands.

In this study, we investigated energy dynamics in live mice through the intact skull using wide-field fluorescence imaging^2^ to monitor local brain blood volume (BBV), as well as cytosolic pyruvate and ATP levels in astrocytes and neurons^3^. We observed a tight coupling between energy dynamics and brain activity, with this association shifting to distinct functional relationships depending on brain state (e.g., non-rapid eye movement (NREM) sleep, REM sleep, and wakefulness)^4^. Given the limited energy availability in the brain, such constraints and competition for energy have likely contributed to the evolution of distinct energy-information coupling strategies. Elucidating energy dynamics in the biological brain represents a key step toward understanding the mechanisms underlying the brain’s unique information-processing capabilities and its unparalleled energy efficiency compared to modern computers^5^.

The firing of an action potential disturbs the ionic environment, requiring energy to restore homeostasis^6^. During an action potential, extracellular Na⁺ enters the neuronal cytosol, followed by the efflux of intracellular K⁺. To restore the ionic balance to the basal state, the Na⁺/K⁺-ATPase actively pumps Na⁺ out of the cell and K⁺ back in, working against their respective concentration gradients using ATP as an energy source. Through the continuous activity of Na⁺/K⁺-ATPase, neurons are able to represent information by firing complex patterns of multiple action potentials. In addition to neurons, astrocytes also express Na⁺/K⁺-ATPase and play a major role in the reuptake of excess extracellular K⁺ released during neuronal activity^7^. It is widely assumed that a substantial portion of the brain’s energy consumption is devoted to fueling Na⁺/K⁺-ATPase activity in both neurons and astrocytes, highlighting the significant energetic cost of maintaining ionic homeostasis across cell types^8,9^.

In addition to action potential generation, synaptic plasticity, which alters signal flow within neuronal networks, also requires significant energy. Cytosolic ATP is essential for synthesizing neurotransmitters, packaging them into synaptic vesicles, and driving synaptic vesicle cycling. Moreover, the synthesis, trafficking, and insertion of receptors, transporters, and ion channels also demand energy. Thus, substantial energy is required to support synaptic plasticity and memory formation.

The ultimate source of brain energy substrates is glucose delivered via the vasculature; however, many pyramidal neurons are not in direct contact with blood vessels. Thus, glucose is often assumed to be primarily taken up by astrocytes. Nevertheless, as extracellular glucose concentrations can be substantial, neurons are also capable of directly taking up glucose^10^. In fact, neurons have been shown to directly take up more than half of blood-borne glucose^11^.

Glucose taken up by neurons is converted into pyruvate and subsequently used for ATP production through mitochondrial metabolism. In contrast, glucose taken up by astrocytes is converted to pyruvate, which can either enter astrocytic mitochondria for oxidative metabolism or be further converted into lactate. Lactate produced in astrocytes can be released into the extracellular space via monocarboxylate transporters (MCTs). This lactate is subsequently taken up by neurons through neuronal MCTs, a process known as the astrocyte–neuron lactate shuttle (ANLS)^12,13^. Lactate taken up by neurons is reconverted into pyruvate and ultimately utilized by mitochondria to generate ATP. The relative contributions of direct neuronal glucose uptake and astrocyte-mediated lactate shuttling to neuronal ATP production likely vary depending on brain state and metabolic demand.

Delivery of energy substrates to neurons can be modulated by several mechanisms. For example, vascular dilation and constriction regulate the initial step of glucose and oxygen delivery^2,14^. This process can be adjusted on demand either through modulation of neurovascular coupling^15–17^ or via neuronal signaling to astrocytes^18^. In addition to receiving glucose from the vasculature, astrocytes can store energy by converting excess glucose into glycogen. In particular, in association with K⁺ uptake during periods of elevated neuronal activity, stored glycogen can be mobilized to generate glucose and subsequently pyruvate, which can be used either for oxidative metabolism within astrocytes or converted into lactate for export.

Under conditions in which astrocyte-mediated substrate transfer plays a dominant role, increased metabolic utilization of pyruvate for ATP production within astrocytes could alter metabolite availability within astrocyte-mediated transfer pathways, potentially creating a trade-off between astrocytic and neuronal energy states. Thus, astrocytes and neurons may exhibit a context-dependent, partially reciprocal relationship in their energy states, depending on the relative contribution of different metabolic pathways. Another level of regulation in astrocyte-mediated energy delivery is the efficiency of MCTs in both astrocytes and neurons. However, neuronal energy supply is supported by multiple parallel routes, including direct glucose uptake. Therefore, the energy states of astrocytes and neurons are likely governed by a flexible balance between these pathways, which may vary depending on metabolic demand associated with different modes of information processing. Conversely, metabolic state may, in turn, influence the properties of neuronal computation^19^, suggesting a bidirectional interaction between energy metabolism and information processing.

ATP is essential for cell survival; therefore, multiple mechanisms likely act to balance ATP consumption and production, ensuring stable cytosolic ATP levels under physiological conditions. Indeed, in vitro studies have shown that neuronal ATP levels decrease only under hyperexcitation induced by strong electrical stimulation or exogenous glutamate exposure, or following pharmacological inhibition of mitochondrial ATP production pathways^20–22^. Consistent with these findings, in vivo studies have demonstrated that neuronal ATP levels remain stable in response to mild sensory stimuli in mice^22^, while a marked reduction in ATP levels is observed during epileptic seizure activity^3^. These observations suggest that brain energy substrates exist in a dynamic equilibrium, with compensatory mechanisms largely maintaining stable energy levels in healthy cells. It is likely that the delicate balance between energy consumption and supply in the brain is continuously adjusted depending on the computational state of the neuronal circuit.

Although sleep is often associated with “rest,” the brain remains highly active, with neuronal activity persisting but exhibiting frequency patterns distinct from those observed during wakefulness^23,24^. During NREM sleep, delta-frequency oscillations in the electroencephalogram (EEG) or electrocorticograms (ECoG) become prominent, although other frequency bands, such as the theta band, are also present, albeit at lower power^25^. In this study, we unexpectedly found that fluctuations in theta-band power, rather than those in the dominant delta band, closely resemble BBV dynamics. Subsequent detailed analysis revealed theta-band ECoG activity during NREM sleep precedes BBV dynamics by ~ 4–5 s and can predict their temporal evolution. This finding suggests a tight coupling between neuronal activity and energy dynamics, which may contribute to maintaining stable levels of energy substrates.

Interestingly, we found that the delicate balance between energy consumption and supply breaks down during the transition to REM sleep. REM sleep is characterized by the emergence of strong theta-band EEG activity and the absence of most other frequency bands^26^ and is associated with processes of memory selection and storage^27^, which are proposed to be reflected as dreams. During this stage, synchronous activity between the hippocampus and cortex is enhanced^28^. These neuronal activities and the formation of synaptic plasticity are thought to require large amounts of metabolic energy. Consistent with this assumption, a marked increase in BBV was observed^4,29,30^, which is generally associated with increased delivery of energy substrates, although this relationship is not directly measured in the present study. In parallel with the BBV surge, cytosolic pyruvate levels measured in astrocytes increased.

However, unexpectedly, a prominent decrease in neuronal cytosolic ATP was observed^31^. This decrease could result from (1) a reduction in the supply of energy substrates (e.g., lactate) to neurons, (2) a decrease in energy production within the neuronal cytosol, or (3) a substantial increase in ATP consumption driven by information processing specific to REM sleep. Although mechanisms (1) and (2) appear unlikely, as they would not support the increased energy demands, if such reductions in neuronal energy supply or production were to occur, this metabolic shift during REM sleep may serve an as-yet-unrecognized adaptive function. This ATP drop may reflect transient local energy redistribution that prioritizes synaptic reorganization^32^ over immediate energy homeostasis.

In this study, we aimed to uncover how brain-state-dependent energy dynamics arise from coordinated interactions among neurons, astrocytes, and vasculature, and how these dynamic metabolic processes underpin distinct modes of information processing across sleep-wake cycles. We propose that the dynamic interaction between information processing and metabolism constitutes a key mechanism underlying the unique characteristics of the biological brain^33^.

## Results

### Visualization of energy dynamics through the intact mouse skull

**Fig. 1:**
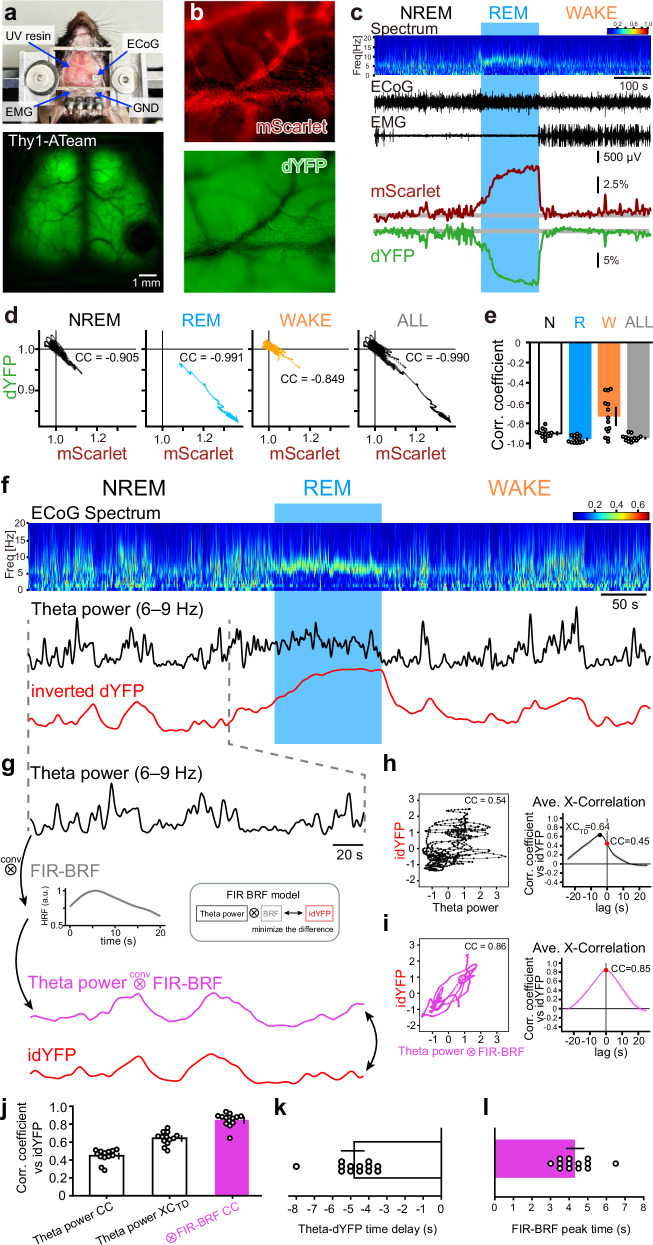
Theta-band power is tightly coupled with brain blood volume changes. **a** Wide-field fluorescence images were acquired through the intact skull using a macro-zoom microscope in head-fixed mice. Shown is an example of a whole-cortex fluorescence image from a Thy1-ATeam mouse. Typically, only the left hemisphere was imaged, as the implanted screw electrode obstructed a part of the contralateral field of view. **b** Fluorescence images of albumin-mScarlet expressed in blood plasma and YFP expressed in neurons. Blood vessels appear as dark regions in the direct YFP (dYFP) image. **c** REM sleep was characterized by prominent theta oscillations (6–9 Hz) in the ECoG and low EMG amplitude. During REM sleep, mScarlet fluorescence intensity increased while dYFP intensity decreased. **d** Scatter plots demonstrating the negative correlation between mScarlet and dYFP fluorescence intensities during NREM, REM, WAKE, and all combined states (CC, Pearson correlation coefficient). **e** Summary of the correlation coefficients (14 episodes from *n* = 3 mice; error bars, SEM). The robust negative correlation indicates that the dYFP signal provides a reliable proxy for inverted BBV changes. **f** Theta-band (6–9 Hz) power of ECoG signal correlated with the inverted dYFP signal. Top to bottom: ECoG power spectrogram, z-scored theta-band power, z-scored idYFP. **g** The theta-band power of ECoG was convolved with a finite-impulse-response brain-blood-volume response function BRF (FIR-BRF) and compared with idYFP. The FIR-BRF that minimized the mean-squared error between the two waveforms was calculated. **h** Scatterplot of idYFP versus theta-band power of ECoG from one NREM sleep episode (left). Cross-correlation functions were calculated across episodes and averaged (right, mean ± SEM, 13 episodes from *n* = 3 mice). The coefficient at zero-time lag (CC) is marked in red; the peak correlation with temporal delay is marked in black (XC_TD_). **i** Scatterplot of idYFP versus theta-band power convolved with FIR-BRF (left). The average cross-correlation function (right) shows a higher peak at zero-time lag compared with the raw theta-band power. **j** Summary of correlation coefficients between idYFP and theta-band signals (13 episodes from *n* = 3 mice; error bars, SEM). The FIR-BRF-convolved theta-band power showed the strongest correlation with idYFP. **k** Time delay at which the cross-correlation between the raw theta-band power and idYFP reached its maximum (13 episodes from *n* = 3 mice; error bars, SEM). Negative values indicate that theta-band power led idYFP. **l**, Peak latency of the calculated FIR-BRF (13 episodes from *n* = 3 mice; error bars, SEM).

Our goal was to elucidate how cerebral energy dynamics couple to information processing. Because craniotomy can markedly alter vascular and astrocytic activity by changing intracranial pressure and possibly provoking inflammation, we imaged brain activity through the native, unthinned skull of live mice. Immediately after skin incision and skull exposure, we coated the bone with a transparent resin to preserve optical clarity. A custom stainless-steel head frame was affixed to the skull, allowing each mouse to be secured in a simplified stereotaxic apparatus beneath a macro-zoom fluorescence microscope. This setup enabled video-rate imaging of the entire cortical surface (Fig. 1a). Electrocorticograms (ECoGs) were obtained with stainless-steel screw electrodes inserted through the skull over one cortical hemisphere and the cerebellum, leaving the contralateral cortex undisturbed for imaging. Electromyograms (EMGs) were recorded from sutured neck-muscle electrodes. Combined ECoG and EMG signals allowed reliable classification of non-rapid eye movement (NREM) sleep, REM sleep, and wakefulness.

Local blood flow is a key determinant of energy distribution in the brain. Three principal descriptors have been proposed: (1) vascular volume, (2) flow velocity, and (3) the oxy/deoxy hemoglobin ratio. Although these parameters are partly interdependent, they can also vary independently, and debate continues over which of them most strongly influences cerebral energy supply and information-processing capacity^34,35^. Here, we focused on fluctuations in vascular volume, referred to as brain blood volume (BBV), within the cortex. Our earlier studies showed that fluorescence emitted from parenchymal cells is attenuated by overlying blood vessels, which therefore appear as shadows in wide-field images^2,4,36^. Green autofluorescence excited by blue light, as well as signals from genetically encoded fluorescent proteins expressed in astrocytes or neurons, are all diminished because hemoglobin absorbs both excitation and emission light^37^. Vessel dilation consequently reduces the detectable parenchymal fluorescence. In this “shadow imaging” paradigm, fluctuations in fluorescence intensity can serve as an inverse proxy for BBV dynamics (Supplementary Movie 1).

To verify this relationship, we used Thy1-ATeam transgenic mice, which express the FRET-based ATP sensor AT1.03YEMK (ATeam) in a subset of neurons^20,38^. Within the cortex, expression is largely confined to layer 5 pyramidal cells. The sensor comprises CFP and YFP joined by an ATP-binding domain. We first analyzed the direct YFP (dYFP) signal obtained by exciting YFP at 505 nm, a wavelength that barely excites CFP; this fluorescence is therefore essentially independent of ATP binding (Fig. 1a). To test whether dYFP fluctuations inversely reflect BBV changes, we visualized plasma with an albumin-mScarlet fusion protein (Alb-mScarlet) expressed from an adeno-associated virus vector targeted to the liver^39–41^. The virus was injected two weeks before imaging. Secreted Alb-mScarlet entered the circulation and labeled the cerebral vasculature (Fig. 1b). Because YFP is absent from blood vessels, the same vessels appeared as dark shadows in dYFP images (Fig. 1b).

Prior work has shown that cerebral blood flow and BBV rise during REM sleep^4^. Consistent with this, Alb-mScarlet intensity increased during REM sleep, whereas dYFP intensity decreased, and the two traces were nearly mirror images (Fig. 1c). Across all vigilance states, dYFP and mScarlet signals were strongly anticorrelated (*n* = 14; NREM, *r* = −0.901; REM, *r* = −0.957; WAKE, *r* = −0.734; all states combined, *r* = −0.947) (Fig. 1d,e). These data confirm that dYFP provides a reliable inverse measure of BBV. Because YFP fluorescence is quenched at acidic pH, cytosolic pH shifts could influence the dYFP signal. However, such pH effects appear minor in our recordings, or they fluctuate in synchrony with BBV. We therefore treat dYFP as an inverse proxy for BBV in all subsequent analyses.

### Theta-band ECoG activity closely correlates with blood volume changes

After establishing that dYFP variations serve as a reliable proxy for BBV fluctuations, we investigated how neuronal activity relates to BBV. We found that BBV fluctuations were tightly coupled to theta-band ECoG power (6–9 Hz), particularly during NREM sleep (Fig. 1f).

Although REM sleep is usually described as a state with strong theta-band activity, it is more precise to view REM as a period of persistently high theta-band power. In contrast, theta-band oscillations recur intermittently during NREM sleep, and their power waxes and wanes over time. During NREM sleep, we observed a loose similarity between the inverted direct YFP (idYFP) and theta-band power (Fig. 1g). Scatter plots confirmed a moderate correlation in NREM (Fig. 1h), whereas correlations were weak in REM and wakefulness (Supplementary Fig. 1b). Cross-correlation analysis showed that the highest correlation between theta-band power and idYFP occurred with a lag of about 4 to 5 s, with theta-band power leading idYFP (Fig. 1h, k). Consequently, the peak lagged correlation (temporally delayed cross-correlation; XC_TD_) consistently exceeded the zero-lag correlation (Pearson correlation coefficient; CC). These findings indicate that ECoG theta-band fluctuations precede BBV changes, implying that vascular fluctuations during NREM sleep can be predicted several seconds in advance from theta-band activity of ECoG.

To capture this neurovascular coupling, we first applied a double-gamma hemodynamic response function (HRF), which is widely used in previous studies^42^ (Supplementary Fig. 1a). Convolving theta-band power with this HRF noticeably strengthened its correlation with idYFP and shifted the cross-correlation peak to zero lag. We next derived an individually optimized finite-impulse-response brain-blood-volume response function (FIR-BRF) that predicts idYFP from theta-band power with higher fidelity (Fig. 1g). This FIR-BRF models how past neuronal activity contributes to current hemodynamic signals, effectively capturing the transformation from neuronal dynamics to blood volume changes. Applying this FIR-BRF further increased the correlation between measured and predicted BBV signals. Because the FIR-BRF is fitted to each recording without assuming a predefined shape, it provides a more flexible representation of neurovascular coupling than the canonical HRF. The optimized FIR-BRF peaked at 4–5 s (Fig. 1l), matching the lag observed in the original cross-correlation.

These findings indicate that theta-band ECoG activity during NREM sleep reliably predicts subsequent BBV fluctuations, suggesting a homeostatic mechanism that dynamically adjusts energy delivery to match ongoing neuronal demands. While the underlying biological mechanism remains unclear (see Discussion for possible interpretations), our findings demonstrate a robust and reproducible link between theta-band dynamics and vascular activity.

### State-dependent large-scale organization of cortical blood volume fluctuations

**Fig. 2:**
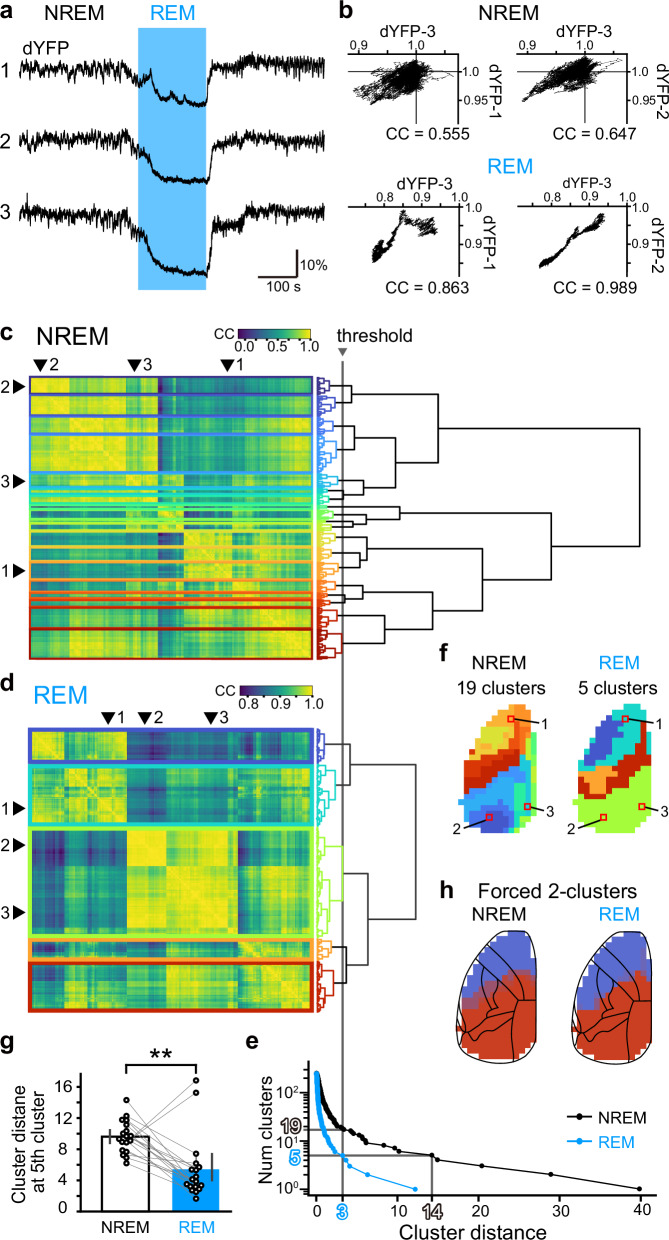
Brain blood volume (BBV) fluctuations globally synchronize during REM sleep. **a** dYFP fluorescence waveforms from three tiles in the left cortical hemisphere (locations shown in **f**). During REM sleep (blue shading), a prominent decrease in dYFP signal, corresponding to increased BBV, was synchronously observed across the cortex. **b** Scatterplots comparing dYFP signals between tile pairs. The spatially closer pair (tiles 2 and 3) showed higher correlations than the distant pair (1 and 3), with correlation strengthening during REM compared to NREM sleep. **c** Correlation matrix of dYFP signals across all tiles during a representative NREM sleep episode. Each row shows correlations between one tile and all others. The raw dYFP waveforms of tiles 1–3 are shown in (**a**), and their spatial location is shown in (**f**). Rows were reordered by hierarchical clustering, with the dendrogram (right) indicating cluster hierarchy. Applying a clustering threshold divided the cortex into 19 regions (**f**, left). **d** Representative correlation matrix during REM sleep. Using the same threshold, fewer (5 regions) but larger clusters emerged, reflecting widespread synchrony of dYFP signals across tiles (**f**, right). **e** Relationship between clustering distance (x-axis) and the number of clusters (y-axis). For any distance threshold, fewer clusters were detected during REM sleep (blue) than NREM sleep (black), indicating stronger synchrony of BBV fluctuations during REM sleep. In this example, clustering into 5 clusters corresponded to distances of 14 (NREM) and 3 (REM). **f**, Spatial clustering of cortical tiles during NREM and REM sleep. **g** Across episodes, the cluster distance yielding 5 clusters was smaller during REM sleep than NREM sleep (*p* = 0.0017, Cohen’s *d* = 1.28,18 episodes from *n* = 3 mice, paired t-test; **p < 0.01; error bars, SEM). **h** Forcing division of the cortex into two clusters consistently revealed an anterior cluster (blue; motor and somatosensory areas) and a posterior cluster (red; occipital and medial cortex) across animals (*n* = 3) in both NREM and REM sleep.

To explore state-dependent changes in blood volume, we analyzed the spatiotemporal dynamics of the dYFP signal across the cortex. Although dYFP waveforms differed among cortical regions (Fig. 2a), BBV fluctuations became synchronized over broader areas during REM sleep than during NREM sleep (Fig. 2b). We quantified this pattern by applying hierarchical clustering to dYFP traces from multiple cortical segments, using their temporal correlations as the similarity metric (Fig. 2c, d). For any chosen distance threshold, the number of clusters was consistently smaller in REM sleep than in NREM sleep (Fig. 2e), indicating greater spatial synchronization of BBV fluctuations in REM sleep (Fig. 2e, g). For instance, a threshold that partitions the cortex into five clusters during REM sleep was used as a reference to enable direct comparison of spatial organization across states. Applying the same threshold to NREM sleep yields nineteen clusters (Fig. 2c, d, f), reflecting reduced large-scale synchronization. Notably, the clustering analysis always segregated the cortex into two principal functional groups in both vigilance states: an anterior (somatosensory–motor) cluster and a posterior (medial–occipital) cluster (Fig. 2h). These results suggest that large-scale spatial organization of BBV fluctuations along the anterior–posterior axis is a persistent feature across sleep states, independent of the overall level of synchronization.

### Spatial characteristics of the fast and slow components of the blood volume dynamics

Because BBV rises slowly and globally during REM sleep, interregional correlations become remarkably high in that state. As a result, the correlation matrix in Fig. 2d is plotted on a color scale restricted to roughly 0.8 to 1.0 for REM sleep, whereas the NREM matrix covers the full span from about 0.0 to 1.0.

To resolve regional similarities and differences more clearly, we separated the dYFP signal into slow (< 0.05 Hz) and fast (0.05–0.2 Hz) components. The 0.05 Hz boundary was chosen based on the temporal characteristics of the REM-associated BBV increase, which unfolds over tens of seconds (period > 20 s), placing it well below 0.05 Hz. This separation therefore isolates the large-scale, state-transition-related vascular component from faster fluctuations. Importantly, the 0.05–0.2 Hz band corresponds to the established frequency range of spontaneous vessel diameter oscillations (vasomotion) reported in rodents^2,43^. Thus, this band captures physiologically defined vascular dynamics rather than an arbitrary frequency range. We note that while alternative cutoffs could be used, this separation provides a biologically interpretable distinction between global, slow vascular shifts associated with brain-state transitions and local, faster vasomotor dynamics.

**Fig. 3:**
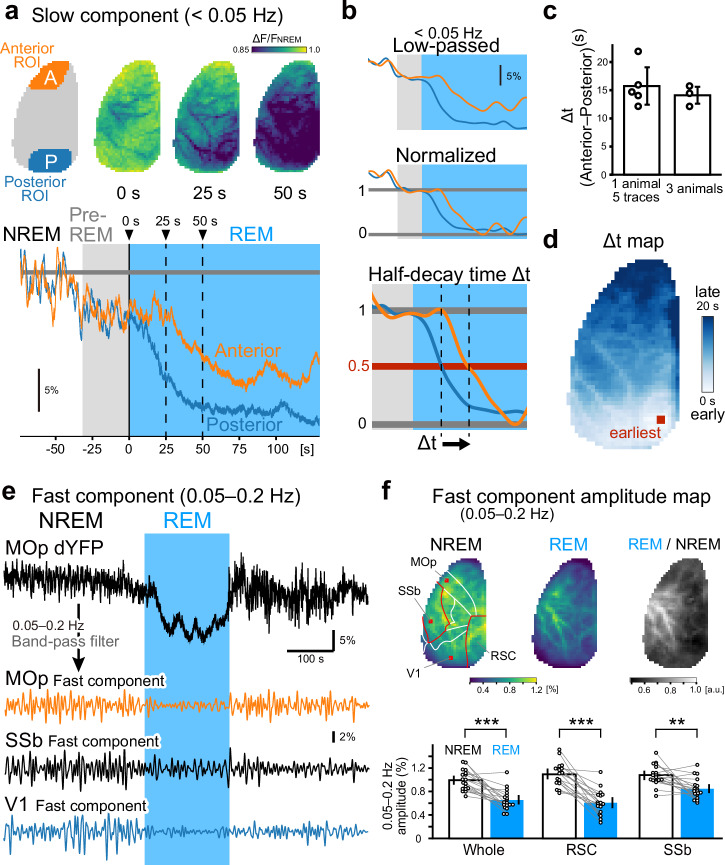
Slow and fast components of cortical blood volume dynamics across sleep stages. **a** Representative snapshots of dYFP fluorescence during the transition from NREM to REM sleep. Anterior and posterior regions of interest (ROIs) are defined in Fig. 5i. Bottom traces: raw dYFP signals from anterior (orange) and posterior (blue) ROIs. At the NREM-to-pre-REM transition, dYFP signals decreased nearly simultaneously in both ROIs, whereas at the pre-REM to REM transition, the posterior signal decreased ∼15 s earlier than the anterior. **b** Schematic of time delay (Δt) quantification. After low-pass filtering and normalization of dYFP signals, Δt was defined as the difference in half-decay times between anterior and posterior signals. **c** The posterior dYFP signal reached its half-decay point ∼15 s earlier than the anterior (15 episodes from *n* = 3 mice; error bars, SEM). **d** Representative Δt map showing propagation of the dYFP signal decrease (reflecting BBV increase) across the cortex. The dYFP signal decrease originated in posterior regions and propagated anteriorly over ∼20 s. **e** Top trace: dYFP fluorescence signal recorded from the primary motor cortex (MOp). Bottom traces: fast components (0.05–0.2 Hz band-pass filtered dYFP signals) from MOp, sensorimotor barrel field (SSb), and primary visual cortex (V1) (locations indicated by red tiles in **f**). The amplitude of the fast component was markedly reduced during REM sleep in MOp and V1. **f** Quantitative analysis of fast-component amplitude (ΔF/F, %). Top: spatial maps of amplitude during NREM, REM, and the REM/NREM amplitude ratio. Amplitude was globally reduced during REM sleep but relatively preserved in SSb. Bottom: comparison of amplitudes during NREM (gray) and REM (blue) for the whole cortex (Whole), retrosplenial cortex (RSC), and SSb. ROI boundaries are outlined in red in (**b**). Amplitude was reduced in all regions during REM sleep (Whole, *p* = 1.9 × 10^–5^, Cohen’s *d* = 1.95; RSC, *p* = 1.7 × 10^–6^, Cohen’s *d* = 2.34; SSb, *p* = 0.0017, Cohen’s *d* = 1.29; 18 episodes from *n* = 3 mice, paired t-test; **p < 0.01, ***p < 0.001; error bars, SEM).

We first analyzed the slow (< 0.05 Hz) component of BBV fluctuations that emerge during the transition from NREM to REM sleep (Fig. 3a-d). A pronounced decrease in the dYFP signal, indicating a synchronous BBV increase, began about 50 s before this REM onset; we refer to this interval as the pre-REM period (Fig. 3a). The dYFP trace was low-pass filtered below 0.05 Hz, set to a baseline of 1 at the pre-REM period, and normalized so that its maximum decrease was 0 (Fig. 3b). We then computed the half-decay time (Δt) across cortical regions. Δt analysis showed that the large-amplitude BBV rise originated in the posterior cortex, first appearing in the retrosplenial cortex (RSC), and reached anterior regions about 15 s later (Fig. 3c, d). This posterior-to-anterior wave likely reflects a state-dependent metabolic shift tailored to REM sleep.

We next focused on the fast component of the dYFP signal (0.05–0.2 Hz). Spontaneous dilation and constriction of cerebral vessels, known as vasomotion, typically occur in this band^2,43^ (Fig. 3e). After band-pass filtering the dYFP signal in this range, we found that the amplitude of rapid BBV fluctuations fell during REM sleep. This reduction was evident across the cortex and was especially pronounced in posterior regions such as the primary visual cortex (V1). Thus, while overall BBV rises slowly and globally during REM sleep, its high-frequency variations diminish. These observations suggest that REM sleep represents a metabolic phase in which BBV increases steadily, but fast BBV fluctuations remain small and stable.

Interestingly, the reduction in the fast dYFP component was less pronounced in the sensorimotor barrel field (SSb) than in other cortical regions. To quantify regional differences, we divided the cortex into a grid, calculated the amplitude of the 0.05–0.2 Hz dYFP component in each tile, and compared the amplitude values between NREM and REM sleep (Fig. 3f). The REM-to-NREM amplitude ratio in SSb tiles was closer to one than in most other regions (white to light gray). In contrast, most other areas, particularly the retrosplenial cortex (RSC), showed marked reductions (dark gray). These findings indicate that the SSb retains relatively stronger fast BBV fluctuations during REM sleep, whereas regions such as the RSC show more pronounced reductions. This pattern suggests region-specific differences in how vascular dynamics are modulated across sleep states, which may relate to underlying differences in local activity demands.

### Spatiotemporal dynamics of the fast component of the blood volume during NREM and REM sleep

**Fig. 4:**
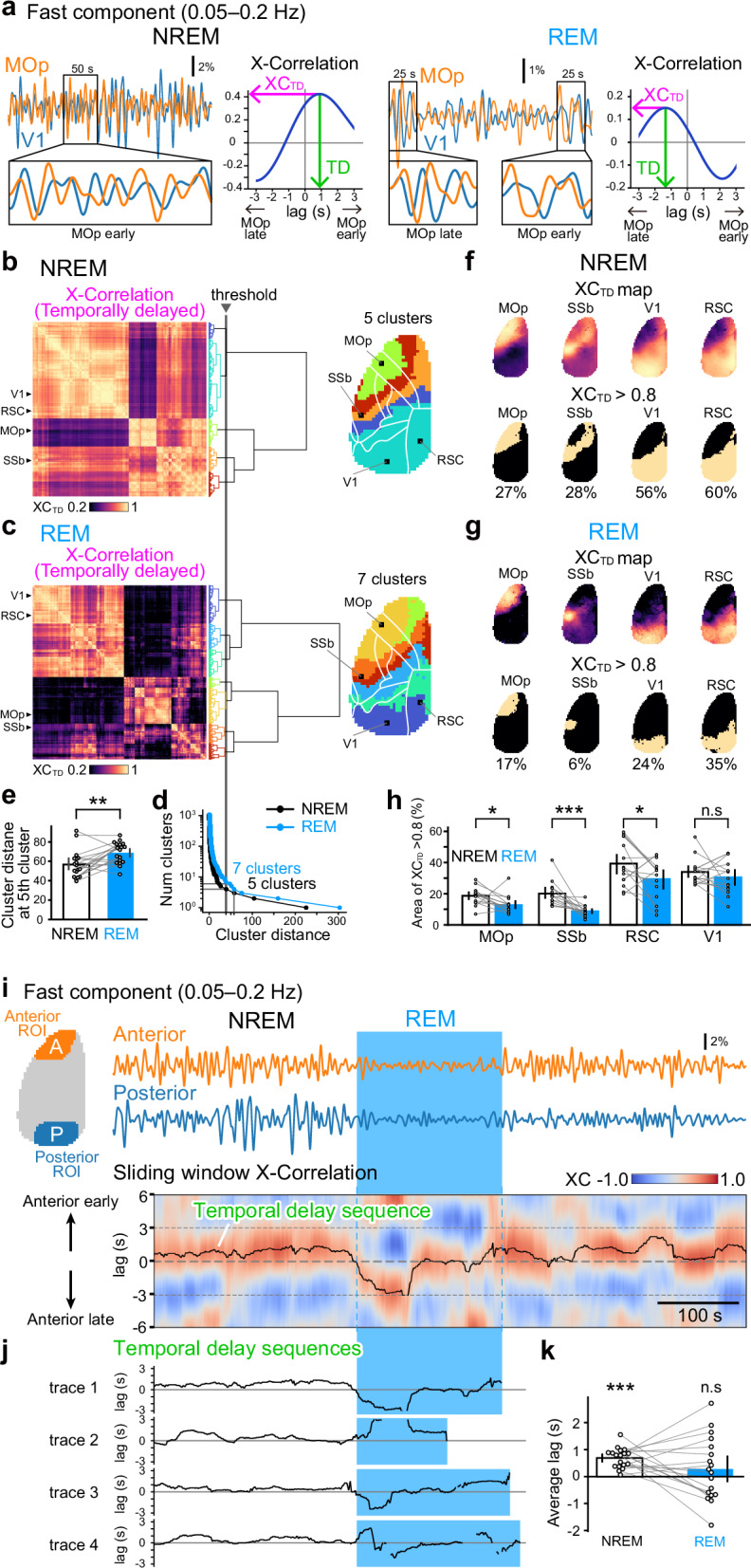
Distinct spatiotemporal dynamics of the fast component of the dYFP signal during NREM and REM sleep. **a** Example of lagged cross-correlation analysis. Band-pass filtered (0.05–0.2 Hz) dYFP signals from MOp and V1 were compared. The peak of the cross-correlation function defines the temporally delayed cross-correlation (XC_TD_), and the corresponding time lag defines the temporal delay (TD). Examples are shown for NREM (left) and REM (right). **b** XC_TD_ matrix of fast-component dYFP signals across cortical regions during a representative NREM sleep episode. Rows in the matrix were ordered by hierarchical clustering, and a distance threshold (gray line) yielded 5 clusters (right map). Seed regions are marked with triangles and remapped in (**f**). **c** XC_TD_ matrix and clustering during REM sleep from the same recording. Using the same threshold resulted in 7 smaller clusters, indicating reduced long-range synchronization of BBV fluctuations compared to NREM sleep. **d** Relationship between cluster distance (x-axis) and number of clusters (y-axis). For any threshold, fewer clusters were detected during NREM sleep (black) than REM sleep (blue), indicating greater synchrony of the fast BBV fluctuations in NREM sleep. **e** Across episodes, the distance yielding 5 clusters was smaller in NREM sleep than REM sleep (*p* = 0.0048, Cohen’s *d* = 0.95,18 episodes from *n* = 3 mice, paired t-test; **p < 0.01; error bars, SEM). **f** XC_TD_ maps during NREM sleep seeded from MOp, SSb, RSC, and V1 (top). Bottom: cortical areas with XC_TD_ > 0.8 and the percentage of total cortical area indicated below. **g** Corresponding XC_TD_ maps during REM sleep. **h** Comparison of cortical areas with XC_TD_ > 0.8. Highly correlated areas were significantly larger during NREM sleep than REM sleep for MOp, SSb, and RSC, but not for V1 (MOp, *p* = 0.015, Cohen’s *d* = 1.06; SSb, *p* = 0.00036, Cohen’s *d* = 1.89; RSC, *p* = 0.042, Cohen’s *d* = 0.74; V1, *p* = 0.33, Cohen’s *d* = 0.36; 15 e*p*isodes from *n* = 3 mice, paired t-test; *p < 0.05, **p < 0.01, ***p < 0.001; error bars, SEM). **i**, Representative fast-com*p*onent traces from anterior (A) and posterior (P) cortex (top). Sliding-window cross-correlation (50 s window) shows dynamic temporal relationships. The heatmap represents the cross-correlation function at each time point, and the black line tracks the time lag of the peak cross-correlation. **j** Additional examples of anterior–posterior temporal delay sequences. **k** Average time lag between anterior and posterior activity. During NREM sleep, anterior signals consistently preceded posterior signals, whereas REM sleep showed variable patterns (NREM, *p* = 4.5 × 10^–7^, Cohen’s *d* = 1.85; REM, *p* = 0.32, Cohen’s *d* = 0.24; 18 episodes from *n* = 3 mice, one-sample t-test, ***p < 0.001; error bars, SEM).

As shown in Figs. 2 and 3, cross-correlation analyses based on raw dYFP signals are strongly influenced by the large-amplitude slow component (< 0.05 Hz). When the fast component (0.05–0.2 Hz) was isolated, distinct fluctuation dynamics became apparent (Fig. 3e–f); however, its amplitude during REM sleep was substantially reduced compared to NREM sleep, making direct comparison of spatial coordination based on amplitude alone difficult. To assess interregional similarity in the fast component of the dYFP signal, we calculated cross-correlation functions between all pairs of cortical tiles (Fig. 4). Importantly, zero-lag correlations, such as those used in Fig. 2, may underestimate coordination when signals are temporally shifted. For example, two regions with similar but time-delayed fluctuations can appear weakly correlated at zero lag. To capture such delayed coordination and to determine whether fast dYFP fluctuations exhibit spatiotemporal propagation across the cortex, we performed lagged cross-correlation analysis across all cortical tile pairs (Fig. 4). This approach allows us to distinguish reduced amplitude from altered spatiotemporal organization of vascular dynamics. Specifically, for each pair of cortical tiles, we computed the lagged cross-correlation function between their band-pass filtered (0.05–0.2 Hz) dYFP signals and defined its peak value as the temporally delayed cross-correlation (XC_TD_), with the corresponding time lag defined as the temporal delay (TD) (Fig. 4a). XC_TD_ thus measures functional connectivity while allowing for a temporal offset.

Computing XC_TD_ and TD across all tile pairs yielded symmetric matrices analogous to a standard correlation matrix, where each entry represents the XC_TD_ or TD between a given pair of cortical tiles (Supplementary Fig. 2c, left). Remapping each row onto cortical space highlights regions most correlated with a given seed tile (Supplementary Fig. 2c, right). The rows were then reordered by hierarchical clustering to reveal spatial groupings of tiles with similar fast-component fluctuations (Fig. 4b, c). Unlike Fig. 2c and d, both matrices are displayed on the same color scale.

Using a single clustering threshold defined based on the NREM data to enable direct comparison, the fast dYFP component partitioned the cortex into five clusters during NREM sleep, arranged roughly along the anterior–posterior axis (Fig. 4b, right; Fig. 4d). Applying the same threshold to REM sleep yielded seven smaller clusters (Fig. 4c, right; Fig. 4d), reflecting increased spatial fragmentation of fast BBV fluctuations. Across all analyzed NREM and REM sleep episodes, the cluster distance needed to generate five clusters was significantly larger during REM sleep (Fig. 4e). The relationship between cluster number and clustering threshold further indicates that fast dYFP fluctuations are spatially more synchronized in NREM sleep than in REM sleep (Fig. 4d, e).

To probe the cluster-size difference, we projected representative rows of the XC_TD_ matrix onto cortical space (Fig. 4f, g). These maps show which cortical regions have fast-component dYFP fluctuations that correlate with those of a chosen seed tile. We then applied a threshold of 0.8 to isolate tiles that were strongly correlated with the seed (Supplementary Fig. 2d). During NREM sleep, the thresholded area covered a large portion of the ipsilateral cortex, indicating widespread spatial synchronization of fast BBV fluctuations. By contrast, in REM sleep, this highly correlated region contracted sharply and remained tightly centered on the seed tile (Supplementary Fig. 2e, f). We quantified this localization by calculating the percentage of cortical area exceeding the high XC_TD_ threshold (> 0.8) and found that localization of the fast component increased during REM sleep in every region except V1 (Fig. 4h; Supplementary Fig. 2g).

We next examined how the fast component of the dYFP signal propagates across the cortex. For each episode, we measured the temporal delay (TD) sequence using a sliding window cross-correlation between signals in anterior and posterior regions of interest (ROIs) (Fig. 4i). Here, TD was defined as the time lag at which the cross-correlation function reached its peak (Fig. 4a), with positive values indicating that the anterior signal leads the posterior signal. This sliding window approach allowed us to track how the leading/lagging relationship between the two regions shifted over time within a single episode. During NREM sleep, the anterior ROI consistently led the posterior ROI by about one second (Fig. 4i, j), indicating a stable front-to-back wave. In REM sleep, however, this orderly pattern broke down. The propagation direction became variable, often reversing within a single REM episode, resulting in a wide fluctuation of the mean lag and a non-significant difference from zero (Fig. 4j, k). These results suggest that fast BBV waves have a consistent anterior-to-posterior flow in NREM sleep but lose directional coherence in REM sleep.

We computed the temporal delay (TD) for every tile pair by cross-correlation and displayed the results as a TD matrix (Supplementary Fig. 3). Each row of this matrix was then remapped onto cortical space to show the TD distribution of all tiles relative to a given seed tile. For each seed, we averaged the row values and projected that mean TD back onto the seed’s location in the original cortical map. Repeating this procedure for every tile produced a row-wise mean TD map, in which color encodes the average timing of each tile’s peak correlation relative to the rest of the cortex: red indicates earlier-occurring signals, blue indicates later ones. During NREM sleep, the map revealed a stable anterior-to-posterior wave, with the anterior cortex leading the posterior by roughly one second. In REM sleep, however, propagation patterns varied: some episodes showed a posterior-to-anterior sequence, others retained the anterior-to-posterior pattern, and still others displayed complex, ambiguous flows. As illustrated in Fig. 4j, the direction of the TD sequence could even alternate within a single REM episode.

In summary, BBV dynamics during NREM sleep are dominated by fast anterior-to-posterior waves that cross the cortex in roughly 1 s. In contrast, during REM sleep, they are governed by a slow posterior-to-anterior wave that sweeps across the cortex in about 15 s, superimposed on smaller, fast fluctuations whose direction is variable (Supplementary Fig. 8).

### Brain-state-dependent emergence of blood volume motifs

**Fig. 5:**
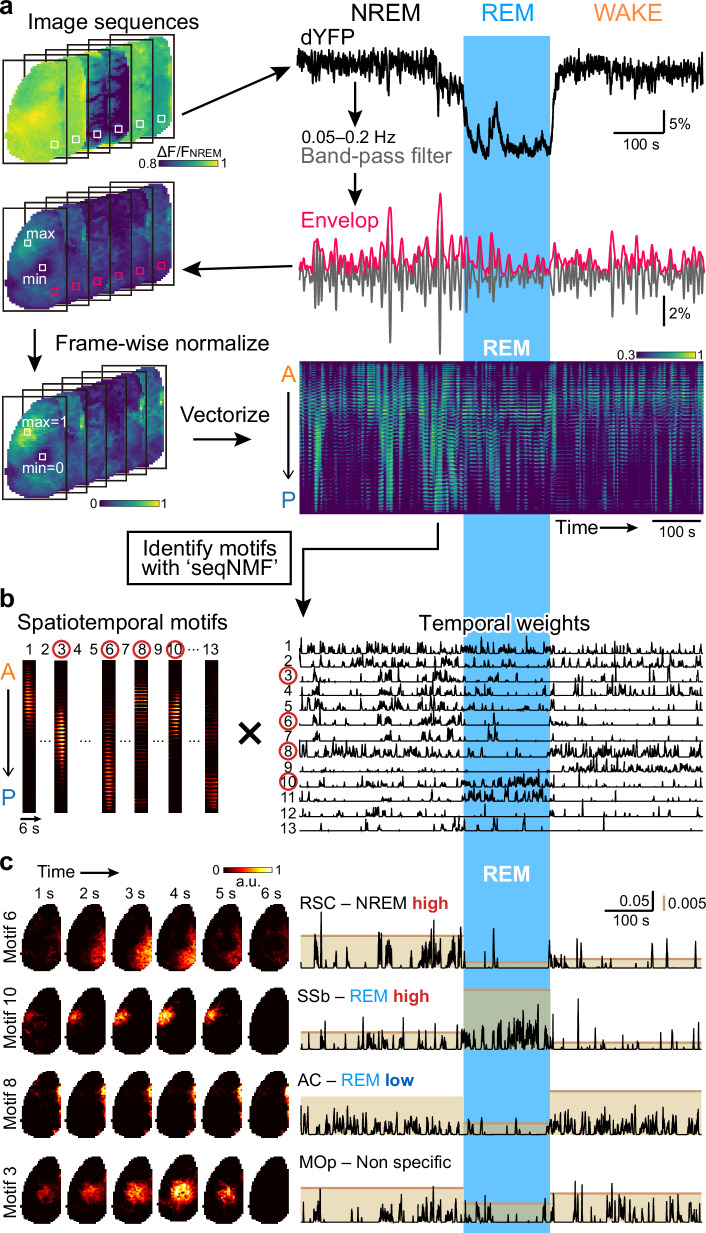
Extraction of spatiotemporal motifs of brain blood volume (BBV) fluctuations. **a** Preprocessing pipeline for unsupervised motif extraction using seqNMF. Time-series image sequences including NREM, REM, and WAKE episodes were collected. dYFP signals from cortical tiles were band-pass filtered (0.05–0.2 Hz) along the time axis (top), and the upper envelope was obtained with a Hilbert transform (pink, middle). Each frame was then min-max normalized, and tiles were vectorized into a time-series matrix (tiles × frames). **b** Using seqNMF, the time-series matrix was decomposed into spatiotemporal motifs (left, 6 s components) and corresponding temporal weights (right). Motifs highlighted in red (3, 6, 8, 10) are shown in detail in (**c**). **c** Four representative motifs (left) with corresponding temporal weights (right). Active areas in motifs 6, 10, 8, and 3 corresponded mainly to RSC, SSb, AC, and MOp, respectively. Their temporal occurrence patterns were distinct: motif 6 (NREM-high), motif 10 (REM-high), motif 8 (REM-low), and motif 3 (non-specific). Notably, motif 6 activity in the RSC gradually increased during NREM and peaked just before the transition into REM (see Supplementary Fig. 4).

We further analyzed the spatiotemporal structure of the fast BBV component (0.05–0.2 Hz). Our goal was to identify short, recurring spatiotemporal patterns (“motifs”) whose frequencies depend on brain state. To this end, we applied seqNMF, an unsupervised matrix-factorization method^44,45^, to the fast component of the dYFP signal (Fig. 5a), and 6 s long motifs were excavated. The extracted motifs (Fig. 5b, left) were grouped manually based on spatiotemporal features into ten types that appeared consistently across sleep episodes and animals (Supplementary Fig. 4a, b; Supplementary Movie 2, 3).

For each motif, we computed a temporal weight, the occurrence rate along the time axis (Fig. 5b, right; Supplementary Fig. 4c, middle column). These weights depended strongly on brain state (NREM, REM, and WAKE; Fig. 5c; Supplementary Fig. 4c). For instance, Motif 10 in Fig. 5c (Motif E, F in Supplementary Fig. 4c), which is largely confined to the sensorimotor barrel field (SSb), occurred most preferentially during REM sleep. Motifs that dominated during NREM sleep included Motifs A, B, I, and J (Supplementary Fig. 4c); their activity centers ranged across motor cortex (MOs), retrosplenial cortex (RSC), and primary visual cortex (V1). Notably, motifs involving the occipital region (e.g., Motif 6 in Fig. 5c; Motifs I and J in Supplementary Fig. 4c) displayed a characteristic temporal profile: their occurrence rose steadily during NREM sleep and peaked just before the transition to REM. This pattern indicates that blood-volume dynamics in the posterior cortex are temporally aligned with the upcoming transition to REM sleep.

### Enhanced blood volume is associated with increases in intracellular metabolite levels

**Fig. 6:**
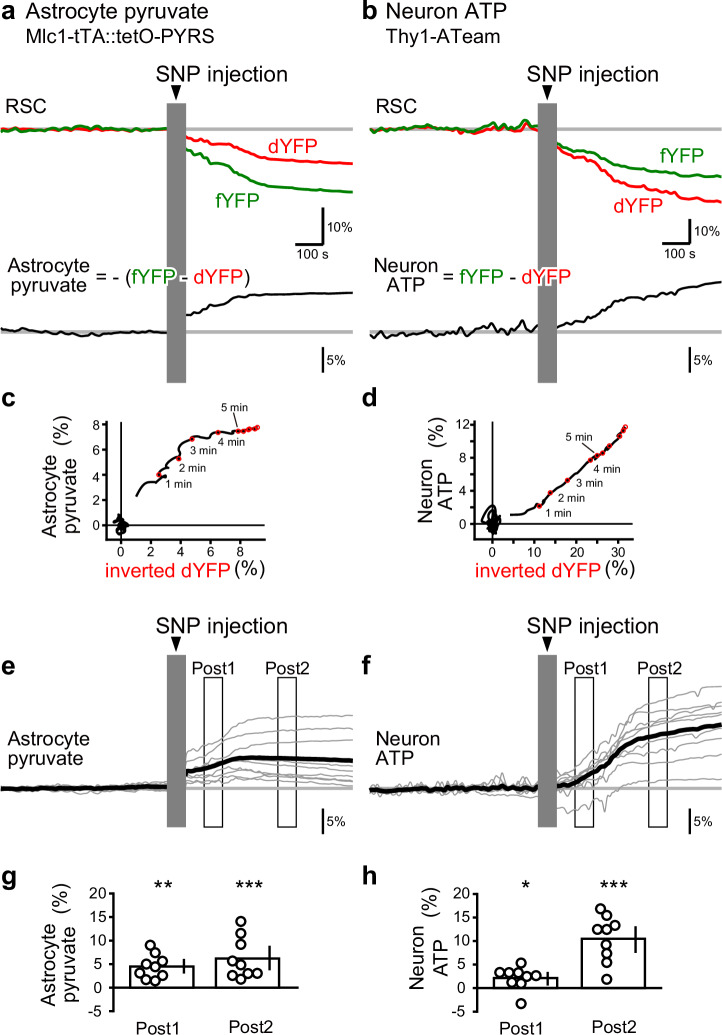
SNP-induced vasodilation increases astrocytic pyruvate and neuronal ATP. **a** A FRET-based cytosolic pyruvate sensor (PYRS) was expressed in astrocytes (Mlc1-tTA::tetO-PYRS mice). The direct YFP signal (dYFP, red; ∼510 nm excitation) served as an indicator of brain blood volume (BBV). The FRET signal (fYFP, green; ∼430 nm excitation) decreases with rising pyruvate. Both dYFP and fYFP decreased after SNP injection (gray bar, 60 s), reflecting increased BBV, but the larger decrease in fYFP indicated elevated astrocytic pyruvate signal estimated by—(fYFP – dYFP). **b** A FRET-based cytosolic ATP sensor (ATeam) was expressed in neurons (Thy1-ATeam mice). Here, FRET efficiency increases with ATP binding, and neuronal ATP signal was estimated by fYFP – dYFP. After SNP injection, the smaller decrease in fYFP relative to dYFP indicated increased neuronal ATP signal. **c** Trajectory of astrocytic pyruvate versus inverted dYFP (idYFP). Pyruvate increased in BBV but reached saturation ∼3 min after SNP injection. **d** Trajectory of neuronal ATP versus idYFP. ATP increased with BBV, but unlike pyruvate, showed no saturation. Averaged pyruvate (**e**) and ATP (**f**) signals across trials (*n* = 9 trials in 3 animals, respectively). Analysis windows: Post1 (1–2 min) and Post2 (4–5 min) after SNP injection. **g** Pyruvate signal increased significantly in both Post1 and Post2 (one-sample t-test with Holm correction; Post1, *p* = 0.00071, Cohen’s *d* = 1.74; Post2, *p* = 0.0029, Cohen’s *d* = 1.41; error bars, SEM). **h** ATP signal increased significantly in both periods (Post1, *p* = 0.028, Cohen’s *d* = 0.89; Post2, *p* = 0.00020, Cohen’s *d* = 2.14), with a stronger rise in Post2. The Post1/Post2 ratio differed significantly between pyruvate and ATP signals (one-sample t-test, *p* = 0.0047; error bars, SEM). Two-way mixed-design ANOVA revealed a significant interaction between signal type and time point, with post-hoc tests showing significance only for ATP between Post1 and Post2 (ATP Post1 vs Post2, *p* = 0.00019; Pyr Post1 vs Post2, *p* = 0.099; Post1 ATP vs Pyr, *p* = 0.13; Post2 ATP vs Pyr, *p* = 0.13).

Our findings revealed complex spatiotemporal patterns of brain blood volume (BBV) fluctuations that depended on brain state. These observations led us to hypothesize that BBV fluctuation patterns may be associated with corresponding changes in intracellular metabolite concentrations, which could in turn relate to the metabolic conditions underlying information processing. To examine whether BBV changes are reflected in pyruvate and ATP concentration dynamics in astrocytes and neurons, respectively, we artificially increased BBV using a vasodilator, sodium nitroprusside (SNP), and monitored fluorescence signals using the respective sensors (Fig. 6).

FRET sensors for pyruvate (PYRS) and ATP (ATeam) were expressed in astrocytes and neurons, respectively, using Mlc1-tTA::tetO-PYRS or Thy1-ATeam transgenic mice. We have previously shown that it is important to consider that BBV and cytosolic pH changes can profoundly affect fluorescence signals. Specifically, fluorescence can be obstructed by blood vessels and thus dilation would result in a decrease in the fluorescence. In addition, it also needs to be taken into account that the sensor protein based on YFP is more readily quenched by acidic pH compared to CFP. Traditionally, when analyzing data obtained with FRET sensors using CFP as a donor and YFP as an acceptor, the ratio between the fluorescence emitted from CFP by exciting with violet light (FRET-CFP or fCFP) is compared with the fluorescence from YFP using the same violet light excitation (FRET-YFP or fYFP). However, if the cytosolic pH decreases, the YFP/CFP ratio may decrease even if there is no change in the concentration of the target-sensed molecule. Therefore, in order to account for the BBV and pH-induced changes, the YFP fluorescence directly excited with blue light (direct YFP or dYFP) was examined, assuming that dYFP is not affected by concentration changes in the target molecule. We also assumed that fYFP and dYFP are affected equally by BBV and pH fluctuations. Then fYFP would differ from dYFP only by its sensitivity to the changes in the target molecule concentration. We also assumed that there is minimal concentration fluctuation of the target molecule, pyruvate or ATP in astrocytes or neurons, respectively during NREM sleep. Consistent with this assumption, fYFP and dYFP fluorescence fluctuation nearly completely matched in their waveforms. Therefore, dYFP was scaled to match the fYFP fluorescence in NREM sleep (see Methods for further discussions on the calculations). The difference between fYFP and scaled dYFP only became prominent during REM sleep or in response to SNP injection. PYRS reduces its FRET efficiency upon binding, so fYFP is expected to decrease relative to dYFP with an increase in pyruvate. On the contrary, fYFP of ATeam is expected to increase with an increase in the ATP.

After SNP administration, an increase in BBV (indicated by a decrease in dYFP and fYFP) was observed as expected (Fig. 6a, b). This increase is expected to enhance the delivery of energy substrates. BBV increase was accompanied by a significant increase in both astrocytic pyruvate and neuronal ATP levels (Fig. 6a, b, g, h). Interestingly, the time course of these changes showed distinct temporal profiles. Astrocytic pyruvate levels increased rapidly in the initial phase (1–2 min post-injection, Post1), followed by a slower increase and plateau in the later phase (4–5 min, Post2) (Fig. 6c, e, g). In contrast, neuronal ATP exhibited a more gradual and sustained increase (Fig. 6d, f, h). Although not directly measured, neuronal pyruvate levels may also increase under these conditions, consistent with known pathways of substrate delivery, including astrocyte-derived lactate and direct glucose uptake. The observed temporal offset between astrocytic pyruvate and neuronal ATP changes is consistent with, but does not establish, a sequential relationship between these variables, in line with the general role of pyruvate as a key substrate for mitochondrial ATP production. It should be noted that, after SNP administration, no ECoG changes were detected during Post1, whereas theta-band power decreased during Post2 (Supplementary Fig. 5).

### Increase in blood volume and theta-band power preceding the transition to REM sleep

**Fig. 7:**
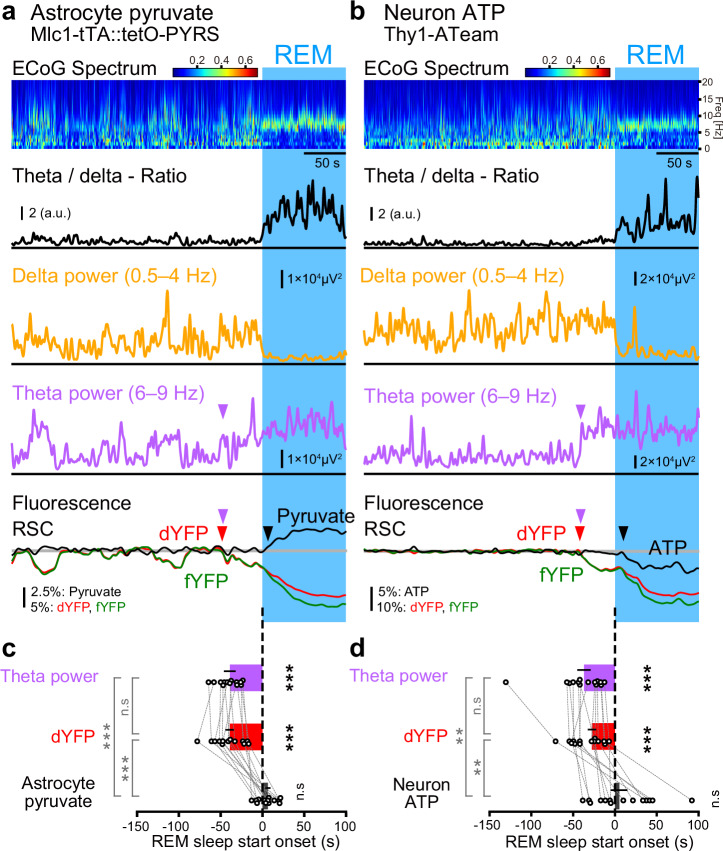
Theta-band power of the ECoG and dYFP changes precede the onset of REM sleep. Representative recordings from Mlc1-tTA::tetO-PYRS (**a**) and Thy1-ATeam (**b**) mice. From top to bottom: ECoG spectrogram, theta/delta power ratio, delta-band power (0.5–4 Hz), theta-band power (6–9 Hz), and optical signals (dYFP, fYFP, and pyruvate or ATP) in RSC. Arrowheads indicate automatically detected change points (purple, theta-band; red, dYFP; black, pyruvate/ATP). **c** Timing of changes in theta-band power of ECoG, dYFP, and astrocytic pyruvate signals relative to REM sleep onset (13 episodes from *n* = 3 mice). Increases in theta-band power and decreases in dYFP occurred significantly earlier than REM sleep onset (one-sample t-test with Holm correction, theta-band power, *p* = 3.0 × 10^–8^, Cohen’s *d* = 2.99; dYFP, *p* = 7.9 × 10^–7^, Cohen’s *d* = 2.12), whereas pyruvate did not (*p* = 0.101, Cohen’s *d* = 0.47). Latencies were similar for theta-band power and dYFP changes, but both preceded pyruvate signal change (one-way ANOVA with Tukey’s post-hoc; theta vs dYFP, *p* = 0.896; dYFP vs pyruvate, *p* = 4.1 × 10^–5^; theta vs pyruvate, *p* = 9.0 × 10^–6^; error bars, SEM). **d** Corresponding analysis for theta-band power, dYFP, and ATP signals (14 episodes from *n* = 3 mice). Theta-band power and dYFP again preceded REM sleep onset, whereas ATP signal did not (one-sample t-test with Holm correction, theta-band power, *p* = 4.5 × 10^–4^, Cohen’s *d* = 1.35; dYFP, *p* = 5.0 × 10^–5^, Cohen’s *d* = 1.76; ATP, *p* = 0.303, Cohen’s *d* = 0.39). No latency difference was found between theta-band power and dYFP, but both preceded ATP signal change (one-way ANOVA with Tukey’s post-hoc; theta vs dYFP, *p* = 0.257; dYFP vs ATP, *p* = 0.00318; theta vs ATP, *p* = 0.0035; error bars, SEM).

To elucidate the sequence of events leading into REM sleep, we performed a detailed temporal analysis of neuronal and metabolic dynamics (Fig. 7). We found that the dYFP signal began to decrease, indicating an increase in BBV, ∼50 s before the REM sleep onset (Fig. 7a, b). The onset of REM sleep, defined by ECoG and EMG, is characterized by a sharp rise in the ECoG theta/delta power ratio. Notably, an increase in ECoG theta-band power was detected earlier, preceding this classical definition of REM sleep onset. The pre-REM increase in ECoG theta-band power was accompanied by a concurrent rise in BBV (Fig. 7c, d).

In contrast, although a prominent BBV increase was evident during this pre-REM period, no significant changes in astrocytic pyruvate and neuronal ATP levels were detected, as the fYFP and dYFP signals remained completely overlapped. The two traces, fYFP and dYFP, from both astrocytic PYRS and neuronal ATeam signals began to diverge only after the sharp rise in the theta/delta power ratio at the defined onset of REM sleep (Fig. 7c, d).

Thus, a transitional phase characterized by neurovascular changes is observed prior to the onset of REM sleep, suggesting the presence of a pre-REM state associated with the transition. Upon REM onset, intracellular metabolite signals exhibit a marked, state-dependent reorganization.

### Dynamics of astrocytic pyruvate and neuronal ATP across sleep states

**Fig. 8:**
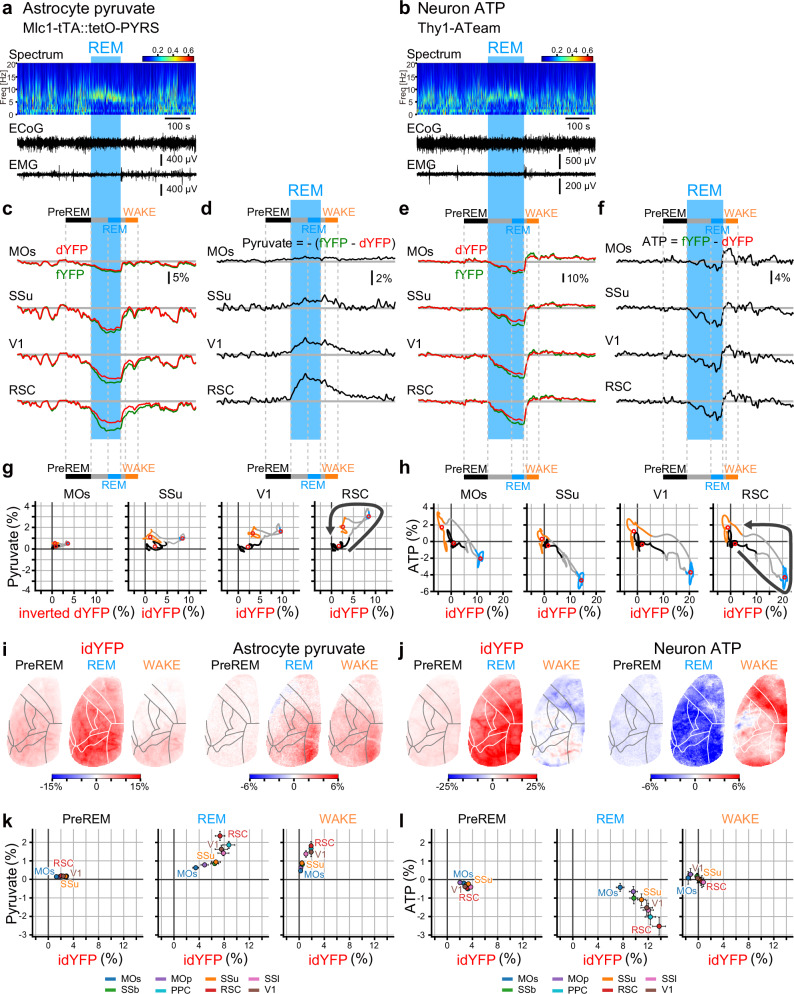
Astrocytic pyruvate increases while neuronal ATP decreases during REM sleep. **a**, **b** Representative recordings from an Mlc1-tTA::tetO-PYRS mouse expressing a pyruvate sensor in astrocytes (panels **a**, **c**, **d**, **g**, **i**, **k**) and a Thy1-ATeam mouse expressing an ATP sensor in neurons (panels **b**, **e**, **f**, **h**, **j**, **l**). ECoG spectrograms, raw ECoG, and EMG traces indicate sleep-wake states. **c**–**f** Fluorescence signals from four cortical regions: secondary motor cortex (MOs), somatosensory upper limb cortex (SSu), primary visual cortex (V1), and retrosplenial cortex (RSC). Astrocytic pyruvate (**d**) and neuronal ATP (**f**) signals were calculated by correcting FRET signal (fYFP) with dYFP to remove blood volume and pH change effects (pyruvate: – (fYFP – dYFP; ATP: fYFP – dYFP). Astrocytic pyruvate (**d**) increased during REM sleep and declined gradually after awakening, whereas neuronal ATP (**f**) decreased during REM sleep and recovered rapidly upon awakening. **g**, **h** Trajectories astrocytic pyruvate (**g**) or neuronal ATP (**h**) signals versus inverted dYFP (idYFP, a proxy for BBV) during pre-REM (100 s before REM onset; black), REM (last 50 s before awakening; light blue), and WAKE (50 s after awakening; orange) periods. Red circles indicate the mean values for each period. Arrows indicate the temporal progression direction of trajectories. Cortical maps of idYFP and astrocytic pyruvate (**i**) and neuronal ATP (**j**), averaged over each sleep period. All cortical maps represent absolute ΔF/F values. **k**, **l** Scatter plots summarizing mean astrocytic pyruvate (**k**) and neuronal ATP (**l**) signal changes against idYFP in eight cortical regions (26 episodes from *n* = 3 mice) for pre-REM, REM, and WAKE periods. Data are presented as mean ± SEM. During REM sleep, posterior regions (e.g., RSC, V1) showed larger increases in idYFP and astrocytic pyruvate and stronger decreases in neuronal ATP. Individual data points for each episode underlying the regional means shown in (**k** and **l**) are provided per cortical region in Supplementary Fig. 7.

Finally, we examined fluctuations in intracellular metabolites during REM sleep. Because blood volume increases during this state, intracellular metabolite levels were expected to rise accordingly. During REM sleep, strong theta-band oscillations appeared in the ECoG while delta waves largely disappeared (Fig. 8a, b). In this phase, astrocytic pyruvate levels increased, as expected (Fig. 8c, d, i; Supplementary Movie 4). In the MOs and SSu regions, the increases in blood volume (reflected by a reduction in the dYFP signal) and pyruvate levels were relatively modest, whereas in V1 and RSC, these changes were more pronounced. Across all regions, elevated pyruvate levels persisted into the awake state and gradually declined thereafter.

Because astrocytic pyruvate is thought to be highly dependent on glucose supplied from the blood^46^, we further analyzed the relationship between the inverted dYFP (idYFP; an index of blood volume) and the pyruvate signal across sleep-wake states (Fig. 8g). In every region, a slight increase in idYFP was observed during the pre-REM period (black trace); however, no corresponding change in pyruvate levels was detected (see also Fig. 7). In contrast, upon entry into REM sleep, both idYFP and pyruvate levels increased in parallel (blue trace).

Interestingly, upon awakening, idYFP promptly returned to baseline NREM sleep levels, whereas astrocytic pyruvate levels remained elevated during the WAKE (shortly after awakening; post-REM) period (orange trace) and declined more gradually toward baseline. Consequently, the relationship between idYFP and astrocytic pyruvate signals was overall positively correlated but displayed a clear hysteresis pattern (Fig. 8g). The mean changes in idYFP and pyruvate signals during pre-REM, REM, and WAKE periods across cortical regions are summarized in Fig. 8k. Notably, the relative increase in astrocytic pyruvate compared with idYFP was particularly prominent in the RSC during REM sleep.

Neuronal ATP levels were similarly analyzed. Unexpectedly, during REM sleep, neuronal ATP levels decreased despite the presumed enhancement of energy supply from increased blood volume (Fig. 8e, f, j; Supplementary Movie 5). Across most cortical regions, the relationship between idYFP and neuronal ATP signals was negatively correlated (Fig. 8h). Both the increase in idYFP and the decrease in ATP levels returned immediately to baseline upon awakening (orange traces). This rapid normalization of neuronal ATP contrasted the lingering elevation of astrocytic pyruvate after REM sleep. The mean changes in idYFP and ATP signals during pre-REM, REM, and WAKE (post-REM) periods across cortical regions are summarized in Fig. 8l.

Notably, the decrease in neuronal ATP occurred alongside an increase in idYFP and was particularly prominent in the RSC and V1 during REM sleep. Regional analysis further revealed an anterior-to-posterior pattern, with idYFP, astrocytic pyruvate, and neuronal ATP showing smaller changes in anterior regions (e.g., MOs, MOp) and larger changes in posterior regions (e.g., RSC, V1). In contrast, regions such as the SSb showed relatively smaller changes across these signals, despite sustained BBV levels.

These data show that both blood supply and astrocytic pyruvate appear to be enhanced during REM sleep; however, neuronal ATP levels paradoxically decreased.

## Discussion

In this study, we demonstrate that brain energy dynamics are not merely passive reflections of neuronal activity^3^, but can diverge from it in a state-dependent manner across behavioral states. By applying wide-field in vivo imaging of blood volume, astrocytic pyruvate, and neuronal ATP levels, we reveal a dynamic and coordinated interplay among neurons, astrocytes, and the vasculature. Distinct from most previous studies, our method required no cranial window; instead, a resin coating applied immediately after scalp removal preserved the natural transparency of the moist, intact skull. This approach enabled the monitoring of unperturbed brain blood volume (BBV) and intracellular metabolite level dynamics under physiological conditions. Collectively, our findings highlight the critical role of the tripartite metabolic network in flexibly coordinating energy supply and consumption to meet computational demands.

Under physiological conditions, neuronal activity and energy supply are tightly coupled through well-established neurovascular and neuron-glia metabolic interactions. During NREM sleep, we found that applying a simple brain-blood-volume response function (FIR-BRF) to the theta-band component of the ECoG could predict brain blood volume (BBV) changes ∼4 s in advance. This finding suggests the presence of homeostatic optimization mechanisms that adjust BBV to minimize metabolic fluctuations and maintain stable neuronal function.

Although the underlying mechanism remains unclear, this temporal relationship is consistent with the involvement of intermediate processes linking neuronal activity to vascular responses^35^. Recent studies have reported that infraslow oscillations in cortical activity are associated with fluctuations in noradrenaline (NA; or norepinephrine, NE) levels and cerebral blood volume during NREM sleep^47–49^. These observations suggest that neuromodulatory dynamics may contribute to the coupling between neuronal activity and vascular responses. In this context, the finding that theta power precedes BBV by several seconds indicates that theta-band activity captures aspects of neuronal dynamics closely associated with subsequent BBV fluctuations during NREM sleep.

Large-scale anterior to posterior propagation patterns were observed upon REM sleep onset, and related propagation dynamics have also been reported under anesthesia^50^, suggesting that such activity may reflect a general organizing principle of large-scale brain dynamics across states. Clustering analyses revealed the patterns of BBV changes differed markedly between NREM and REM sleep (Supplementary Fig. 8). We also identified multiple short spatiotemporal motifs of BBV dynamics, with particular motifs preferentially occurring in specific brain states and presumably associated with distinct information-processing modules. Specifically, the sensorimotor barrel field (SSb) was characterized by a REM sleep-specific fast component of BBV activity, with confined correlated cluster areas, only a small reduction in amplitude, and the presence of motifs with high occurrence during REM. This may reflect a replay of virtual whisker-guided navigation during mouse dreams^51^. Together, these observations indicate that BBV dynamics are finely regulated to meet the energy demands of recurring functional modules engaged across behavioral states.

We observed a surge in BBV beginning 50 s before the classically defined onset of REM sleep, which is marked by a sharp increase in the ECoG theta/delta power ratio. Interestingly, astrocytic Ca²⁺ has been reported to rise about 20 s prior to the onset of epileptic neuronal hyperactivity^52^. Together, these findings suggest that astrocytes and the vasculature may not only respond passively to neuronal activity but may also exhibit dynamics that precede changes in neuronal network states^39,53–55^. These dynamics may be associated with broader state-dependent changes, including metaplastic states^2,56–58^. We further found that the BBV surge during the transition into REM sleep originates in the occipital cortex, implicating the RSC as a potential regulator initiating REM sleep^59^.

Notably, despite this pre-REM BBV surge, neither astrocytic pyruvate nor neuronal ATP levels changed, indicating that the balance of energy supply and consumption remained intact during this pre-REM phase. Only after REM onset did the fYFP and dYFP fluorescence signals diverge in both PYRS and ATeam sensors, reflecting changes in metabolite concentrations in astrocytes and neurons. No pyruvate or ATP changes observed during the pre-REM BBV surge, contrasts with the SNP experiment (Fig. 6), in which pharmacological vasodilation was accompanied by increases in both pyruvate and ATP. The pre-REM BBV increase occurs alongside a concurrent rise in theta-band activity, so that the enhanced substrate delivery may be offset by a corresponding increase in energy consumption, resulting in no net change in metabolite levels. In contrast, SNP-induced vasodilation increases BBV without an accompanying rise in neuronal activity (Supplementary Fig. 5), so that the additional substrate supply may not be consumed, which would be reflected as detectable increases in pyruvate and ATP signals.

These pre-REM hemodynamic changes may also be consistent with known state-dependent modulation of neuromodulators such as NA and acetylcholine, which are known to influence vascular tone. Optogenetic inhibition of locus coeruleus (LC) reduces NA levels and can induce REM sleep^47,48,60^. The resulting vasodilation may reflect a state-dependent process that could support substrate availability for the metabolic demands of the upcoming REM state. However, as these variables were not directly measured in the present study, their contribution remains to be determined.

The balance between energy supply and consumption shifts during REM sleep. Alongside the increase in cerebral blood volume, astrocytic pyruvate levels also increased. This pyruvate increase may reflect a combination of enhanced substrate delivery from the vasculature and elevated glycolytic activity within astrocytes.

Here, we use the term “paradox” to denote an apparent inconsistency under a simplified assumption, namely, that increased substrate delivery would be expected to elevate intracellular ATP levels. This usage echoes the classical term “paradoxical sleep,” in which REM sleep exhibits wake-like neuronal activity despite behavioral quiescence. Under this assumption, an increase in cerebral blood volume would be expected to elevate the availability of energy substrates to neurons, whether through direct uptake from the vasculature or via astrocyte-mediated transfer (e.g. ANLS), leading to an increase in neuronal ATP levels. However, despite this expected increase in substrate availability, neuronal cytosolic ATP levels markedly decreased during REM sleep. This dissociation indicates that substrate availability alone is insufficient to account for neuronal ATP dynamics, suggesting that additional factors must be considered.

This ATP decrease could result from one or more nonexclusive mechanisms: (A) increased ATP consumption within neurons, (B) reduced energy transfer from astrocytes to neurons, and (C) impaired ATP production within mitochondria (Supplementary Fig. 9). We first considered the possibility of (A), increased neuronal ATP consumption.

An acute increase in ATP demand in neurons during REM sleep could contribute to the observed ATP reduction. However, several observations suggest that increased neuronal firing alone is unlikely to fully account for the magnitude of ATP decrease observed here. Neuronal ATP is thought to be used primarily by the Na⁺/K⁺-ATPase to restore ion gradients disrupted by action potential firing. However, the rate of action potential firing reportedly increases only modestly (<1.3-fold) in the retrosplenial cortex (RSC) and primary visual cortex (V1), while decreasing in motor and somatosensory cortices during REM sleep^61–63^. Moreover, our previous study on epileptic seizures found no clear correlation between seizure duration (a proxy for total firing) and the magnitude of ATP reduction^3^, suggesting that firing-driven ATPase activity is unlikely to primarily account for the observed ATP decrease.

REM sleep state is characterized by highly synchronous neuronal activity, prominent theta-band oscillations, enhanced hippocampo-cortical communication, and memory-related synaptic reorganization, all of which are presumed to require substantial energy resources. Therefore, ATP consumption may be increased by these presumably energy-intensive processes. This interpretation is supported by our observation that the ATP reduction was most pronounced in the RSC, a region that strongly synchronizes with the hippocampus during REM sleep, plays a central role in spatial memory formation, and is implicated in both the initiation and maintenance of REM sleep^59,64^. In addition, our earlier epilepsy study also suggested that ATP is consumed for large-scale neuronal circuit reorganization following seizures^3^. Collectively, these findings suggest that neuronal ATP consumption during REM sleep may reflect not only the maintenance costs of ion pumping but also the energetic demands of large-scale processes such as memory reorganization and sleep-state transitions.

These considerations indicate that while increased ATP consumption likely contributes, it may not be sufficient to fully explain the observed ATP decrease. We therefore considered additional mechanisms that could limit neuronal ATP availability independently of, or in conjunction with, increased consumption. Concurrently, transient reductions in astrocyte-to-neuron substrate transfer, possibly through modulation of monocarboxylate transporter (MCT) activity, may limit neuronal ATP synthesis (mechanism B, above). Impaired MCT efficiency in astrocytes could lead to pyruvate accumulation within astrocytes while restricting lactate delivery to neurons. Supporting this idea, astrocytic acidification during REM sleep has been reported in the lateral hypothalamus^4^. Whether similar acidification occurs in cortical astrocytes remains unknown; however, pH and Ca²⁺ shifts in astrocytes may plausibly modulate MCT activity. In addition, concurrent reductions in neuronal MCT activity could further explain the reported rise in extracellular lactate levels during REM sleep^65^.

Emerging evidence also suggests that mitochondrial oxidative phosphorylation may be downregulated during REM sleep, favoring a shift toward glycolytic ATP production (mechanism C, above). Astrocytes may become acidified due to proton cotransport during glutamate reuptake, which could lead to reduced mitochondrial oxygen consumption within astrocytes^66^. This metabolic shift toward glycolysis is hypothesized to limit reactive oxygen species (ROS) production and preserve oxygen availability for neuronal use. Neurons may likewise shift toward glycolysis, a less efficient but faster ATP production that generates fewer ROS, thereby enabling rapid synaptic plasticity while protecting mitochondria from oxidative stress. Such protection is crucial, as ROS impair mitochondrial ATP synthesis and their suppression is essential for sustaining neuronal activity^67,68^. Supporting this glycolytic shift hypothesis, PET studies in humans have reported increased cerebral blood flow and glucose utilization without a corresponding rise in oxygen consumption during REM sleep^69^. Previous studies have shown that cortical tissue can intermittently become locally hypoxic even under healthy conditions^70^, suggesting that such limitations of oxygen utilization may itself serve an adaptive function. This shift toward glycolysis would also be expected to promote pyruvate accumulation, which is consistent with the elevated astrocytic pyruvate levels observed during REM sleep (Fig. 8).

The functional significance of this metabolic reorganization remains speculative yet compelling. The transient reduction in neuronal ATP may represent a state of prioritized resource allocation, in which short-term energy homeostasis is temporarily compromised to support processes critical for memory reorganization and emotional processing during REM sleep. Such adaptive reallocation of energy could constitute a fundamental strategy that balances immediate energy demands with the preservation of long-term synaptic health and stability.

Collectively, our findings suggest that brain energy metabolism operates within a dynamic hierarchy, in which conventional homeostatic relationships between blood flow and metabolism can be flexibly modulated across brain states. Importantly, the dissociation observed here between neuronal ATP levels and blood volume highlights a limitation of interpreting hemodynamic signals as direct proxies for neuronal energy status. This dissociation is not simply the coexistence of increased blood flow and energy demand, but reflects a deviation from the expectation that increased substrate delivery would be accompanied by increased intracellular ATP levels. While fMRI signals reliably reflect changes in blood flow and vascular dynamics, they do not necessarily provide a direct measure of neuronal energy availability or the balance between energy supply and consumption. Our results suggest that additional layers of regulation, including astrocyte-mediated processes and intracellular metabolic states, can decouple vascular signals from neuronal energy levels (Supplementary Fig. 10). These findings indicate that increases in blood flow do not always translate into increased neuronal ATP availability, and that the relationship between vascular and metabolic signals is more complex than commonly assumed. By directly visualizing components of this pathway, our study provides a framework for understanding how vascular signals, metabolic processes, and neuronal activity are differentially coordinated across brain states. The state-dependent shifts in metabolite levels uncovered here provide new insights into how the biological brain supports complex computation under dynamic metabolic constraints.

Further studies will be required to directly investigate the molecular regulators of astrocyte-neuron substrate transfer and mitochondrial function during REM sleep. Elucidating these mechanisms may not only deepen our understanding of cognitive function but also provide critical insights into the metabolic vulnerabilities that underlie sleep disorders, neurodegenerative diseases, and psychiatric conditions associated with disrupted energy regulation.

## Methods

### Animals

This study was conducted in accordance with the Regulations for Animal Experiments and Related Activities at Tohoku University, and all procedures were approved by the Institutional Animal Care and Use Committee of Tohoku University (2019LsA-017). Efforts were made to minimize animal suffering and reduce the number of animals used. Mice were maintained at 24–26 °C under a 12 h light-dark cycle, with ad libitum access to standard chow and water. After weaning, mice were housed with same-sex littermates to promote environmental enrichment. At the conclusion of the experiments, animals were euthanized by placement in a sealed chamber containing vaporized isoflurane (~1 mL per liter of chamber volume), inducing anesthetic overdose via inhalation, and death was confirmed by the absence of respiration. A total of 15 male mice older than 15 weeks of age were used. Two transgenic lines were studied: Thy1-ATeam mice, expressing a FRET-based ATP sensor in neurons, and Mlc1-tTA::tetO-PYRS, expressing a FRET-based pyruvate sensor in astrocytes.

*Thy1-ATeam* transgenic mice were used to monitor cytosolic ATP concentration dynamics in neurons. Details of this transgenic line are described elsewhere^20^; briefly, expression of the fluorescence resonance energy transfer (FRET)-based ATP sensor ATeam1.03^YEMK^ (hereafter referred to as ATeam^38^) was driven by the neuron-specific Thy1 promoter. ATeam consists of cyan fluorescent protein (CFP) and yellow fluorescent protein (YFP), linked by the ε subunit of *Bacillus subtilis* F₀F₁-ATP synthase, which serves as the ATP-specific binding domain. In the absence of ATP, the extended and flexible conformation of the ε subunit keeps the two fluorophores separated, resulting in reduced FRET efficiency. Upon ATP binding, the ε subunit undergoes a conformational change that brings the fluorophores closer together, thereby increasing FRET efficiency. The apparent dissociation constant (Kd) of ATeam1.03^YEMK^ for ATP is 1.2 mM.

*Mlc1-tTA::tetO-PYRS* double-transgenic mice were used to monitor cytosolic pyruvate concentration dynamics in astrocytes. In *Mlc1-tTA* transgenic mice, the astrocyte-specific *Mlc1* promoter drives expression of the tetracycline transactivator (tTA)^71^. The *tetO-PYRS* knock-in mice were generated by inserting a *tetO-PYRS-hGHpA* cassette into the 3′ untranslated region of the mouse *Actb* gene using CRISPR/Cas9-mediated genome editing, as previously described^72^. *Mlc1-tTA* mice were crossed with *tetO-PYRS* knock-in mice to obtain double-transgenic animals. In these mice, astrocyte-specific expression of tTA activates the *tetO* promoter, leading to expression of the FRET-based pyruvate sensor PYRS^3^ (Niino et al., manuscript in preparation) in a subpopulation of astrocytes^73^. PYRS was constructed by linking CFP and YFP to the pyruvate dehydrogenase regulator (PdhR) from *Escherichia coli*, which serves as the pyruvate-binding domain. The *PdhR* gene was synthesized with mammalian codon optimization and further modified for the construction of PYRS. The apparent dissociation constant (Kd) and Hill coefficient for pyruvate binding were determined to be 26 µM and 1.2, respectively. The nucleotide sequence of the *PYRS* cDNA has been deposited in the DNA Data Bank of Japan (DDBJ)/European Molecular Biology Laboratory (EMBL)/GenBank under accession number LC849112. Ligand binding modulates the conformational relationship between CFP and YFP, thereby altering FRET efficiency. Similar to other previously reported PdhR-based FRET sensors^74,75^, increasing pyruvate concentrations result in enhanced CFP emission and reduced YFP emission. The conformational change induced upon pyruvate binding likely increases the distance between CFP and YFP, thereby decreasing FRET efficiency.

### Visualization of brain blood volume dynamics

To directly measure dynamic changes in brain blood volume (BBV), blood vessels were fluorescently labeled by expressing in the liver a fluorescent protein, mScarlet, fused to the C-terminus of albumin (Alb)^39,40^. Adeno-associated virus serotype 8 (AAV8)^40,41^ was used for gene delivery. The Alb-mScarlet fusion protein expressed in the liver was secreted into the bloodstream and incorporated into the blood plasma. AAV8/P3-Alb-mScarlet was produced at the Viral Vector Core of Gunma University using an ultracentrifugation protocol^76^. The viral vector was dissolved in phosphate-buffered saline (PBS) at a concentration of 2 × 10^12^ vg/mL. This solution was administered systemically via intraperitoneal (i.p.) injection at 270 µL per mouse. AAV injection was performed 7 days after the surgery described below. Following a 2-week period to allow stable Alb-mScarlet fluorescence expression in the bloodstream, imaging experiments were conducted. mScarlet fluorescence intensity increase correlated with increases in blood vessel dilation, and thus with local BBV increases. The mScarlet fluorescence dynamics also showed an inverse correlation with directly excited YFP fluorescence (dYFP) expressed in astrocytes and neurons, indicating that the inverted dYFP (idYFP) could serve as a surrogate measure of BBV dynamics.

### Surgical procedures

Prior to surgical procedures, mice were lightly anesthetized with isoflurane. Once unresponsiveness to toe-pinch was confirmed, deep anesthesia was induced via intraperitoneal (i.p.) injection of a mixed anesthetic solution containing 0.75 mg/kg medetomidine hydrochloride (Domitor; Nippon Zenyaku Kogyo Co., Ltd., Fukushima, Japan), 4 mg/kg midazolam (Midazolam; Sandoz Inc., Japan), and 5 mg/kg butorphanol tartrate (Vetorphale; Meiji Seika Pharma Co., Ltd., Tokyo, Japan). The concentration of each component in the mixture was adjusted so that the volume of injection at 0.1 mL per 10 g of body weight achieved the intended concentration.

To prevent ocular dryness and contamination from disinfectants, the eyes were covered with petroleum jelly. After shaving the fur on the head, the exposed area was disinfected with ethanol, and the scalp was incised to expose the cranium. The anesthetized mouse was then secured to auxiliary ear bars and mounted on a stereotaxic frame (Narishige, Tokyo, Japan) for surgery. The frame height was adjusted to align the bregma and lambda at the same horizontal level.

A stainless-steel wire electrode (AS 633; Cooner Wire, CA, USA) was sutured to the neck muscle for electromyography (EMG) recordings. Prior to implanting stainless-steel screws for electrophysiological recordings, two cranial holes (each slightly less than 1 mm in diameter) were drilled above the right hemisphere at the primary visual cortex (V1) and over the right cerebellum. A 1 mm diameter screw (standard metric M1) was inserted into each hole using a fine Phillips head screwdriver until the tip of the screw just contacted the brain surface. The screw implanted in the cerebellum served as the common ground, while the screw placed over the cerebral cortex was used for intracranial electroencephalography (EEG), or more specifically, electrocorticography (ECoG) recordings.

A stainless-steel head chamber frame (CF-10; Narishige, Tokyo, Japan) was used as a holding jig to stabilize the mouse’s head under the wide-field macro-zoom microscope. The chamber frame was positioned horizontally and attached only to the anterior and posterior edges of the exposed cranium using instant adhesive (Loctite 454 J; Henkel), ensuring that the cortical surface remained unobstructed for imaging. Lead wires from all three electrodes (one wire electrode for EMG, one screw electrode over the cortex, and one screw electrode over the cerebellum) were soldered to a multi-pin connector assembly. To secure the screw electrodes to the skull and prevent short circuits, the gaps between the wires were filled with instant adhesive (Loctite 454 J), which also served as electrical insulation.

To observe cerebellar blood flow and energy metabolite dynamics, it was essential to secure a stable and intact optical window over the brain. To achieve this, a thin layer of UV-curable resin (Jelly Nail; IML Inc., Tokyo, Japan) was applied to the skull surface and cured with UV light. In mice, the acutely exposed cranium is initially highly transparent; however, desiccation rapidly renders the bone completely opaque. The resin coating prevented dehydration of the skull, thereby maintaining its transparency for over a month. The resin and brief UV exposure, which are routinely used in human fingernail applications, pose no health concerns. Moreover, the short UV exposure required for curing did not induce photobleaching of the fluorescent proteins expressed in the mouse brain. Since part of the right cortex was covered by the screw electrode used for ECoG recordings, imaging was primarily performed on the exposed left hemisphere in this study.

To reverse the effects of medetomidine following surgery, 0.75 mg/kg of atipamezole hydrochloride (Antisedan; Nippon Zenyaku Kogyo Co., Ltd., Tokyo, Japan) was administered at a volume of 0.3 mL. The administered butorphanol upon surgery functions as an analgesic, providing pain relief for several hours post-surgery. To facilitate recovery, animals were housed in cages placed on warming plates set to ~30 °C for several days. Water was provided in gel form to ensure adequate intake during this period, and nesting material was supplied to promote environmental enrichment. Following surgery, each animal was housed individually to prevent damage to the implanted apparatus by cage mates. Throughout the 7-day post-surgical recovery period, animals were monitored twice daily for health status. If any signs of distress or minor weight loss were observed, the animal was immediately euthanized to ensure humane treatment. Initial electrophysiological and macro-zoom imaging tests were performed to verify signal integrity. If any abnormalities were detected, the animal was promptly euthanized without further experimentation. Only animals that passed these tests were included in the study, and no animals were excluded after data collection commenced.

### Pharmacological dilation of blood vessels

To pharmacologically dilate blood vessels, sodium nitroprusside (SNP) was used. SNP was dissolved in saline (150 mg/mL), dispensed into 3 µL, and kept at −25 °C until immediately before use. For the administration, the solution was further diluted with saline, and 300 µg SNP was subcutaneously injected into the cervical region.

### ECoG and neck EMG recording

During wide-field macro-zoom imaging experiments, the multi-pin male connector assembly mounted on the skull was connected to multiple electrical cables terminated with a multi-female pin connector. The wires connected to the screw electrodes in the cortex and cerebellum were routed to a bioelectrical amplifier (cat. no. AB-611J; Nihon Kohden, Tokyo, Japan), while the wires from the neck-muscle electrode and the cerebellar common ground electrode were routed to a separate amplifier. ECoG signals were amplified 2000-fold, band-pass filtered between 0.5 and 300 Hz, and digitized at 2 kHz. EMG signals were amplified 5000-fold, band-pass filtered between 50 and 300 Hz, and digitized at 2 kHz. The screw electrode placed over the cerebellum served as the common ground. Both signals were digitized using Spike2 software (version 7.20; Cambridge Electronic Design Ltd., UK), which controlled the Micro1401-3 plus ADC12 top box (CED, Cambridge, UK). Temporal synchronization between the electrophysiological recordings and the optical measurements was achieved using a waveform generator (AWG-50; ELMOS, Japan).

### Sleep stage detection

REM sleep was identified from ECoG recordings by a reduction in delta-band activity (1–4 Hz) and an increase in theta-band activity (6–9 Hz) in the absence of large EMG fluctuations. ECoG signals were processed using the Fourier transformation (window width: 7 s; step width: 0.7 s) to generate a time series of the square root power ratio between 6–9 Hz and 1–4 Hz (theta/delta power ratio). REM sleep was defined as periods when the theta/delta power ratio exceeded a predefined threshold while EMG values remained below a set threshold. If the theta/delta power ratio transiently fell below the threshold, these intervals were still considered part of the same REM sleep episode if separated by less than 20 s. Only REM sleep episodes lasting 50 s or longer were included in the analysis. Periods following REM sleep that consistently exhibited high EMG activity were classified as the awake state.

### Wide-field macro-zoom imaging

The stainless-steel chamber frame (CF-10) attached to the cranium of the mouse was fixed onto a MAG-A holder (Narishige, Tokyo, Japan), which was then mounted on a MAG-1 chamber. A custom plastic cylinder was attached to the MAG-1 chamber to loosely restrain the mouse’s body. The head-fixed mouse was placed under a wide-field macro-zoom microscope (MVX10; Olympus, Tokyo, Japan) equipped with image-splitting optics (W-VIEW GEMINI A12801-01; Hamamatsu Photonics, Shizuoka, Japan) and a digital CMOS camera (ORCA-Flash4.0 V3; Hamamatsu Photonics). The mouse was kept under the microscope for over 30 min to allow stable occurrences of sleep phases, including NREM and REM sleep, during the measurements. However, each measurement session was limited to less than 4 h. Throughout each session, the behavior and general condition of the mouse were monitored; if an animal showed vigorous body movements or signs of poor condition, the session was discontinued and the corresponding data were excluded from analysis.

An LED-based multi-color excitation light source (Niji; Blue Box Optics, Blackwood, UK) was attached to the fluorescence microscope (MVX10). (1) Excitation of CFP of PYRS and ATeam, (2) direct excitation of the YFP (dYFP) of PYRS and ATeam, and (3) excitation of mScarlet were achieved using (1) a 445 nm (Royal Blue) LED light source with a band-pass filter ET430/24x (Chroma Technology, VT, USA), (2) a 505 nm (Cyan) LED light source with a band-pass filter ET505/20x (Chroma), and (3) a 556 nm (Green/Yellow) LED light source with a band-pass filter ET580/25x (Chroma), respectively. Importantly, the 505 nm Cyan excitation (ET505/20x) selectively excited YFP without exciting CFP, allowing the directly excited YFP (dYFP) signal to be measured independently of FRET. A multi-band beamsplitter (69008bs; Chroma) was used to deliver multiple excitation wavelengths to the mouse while allowing the emission signals from CFP, YFP, and mScarlet to pass through. Using the W-VIEW GEMINI image-splitting optics, emissions from CFP (FRET-influenced CFP, fCFP) and mScarlet were transmitted, while emission from YFP (FRET-influenced YFP, fYFP and dYFP) was reflected by a multi-band mirror (ZT532dcrb; Chroma). The emission wavelengths were further filtered using a custom band-pass filter (passband: 473 ± 12 nm and 625 ± 15 nm, Chroma) for fCFP and mScarlet detection in Frame Segment 1, and a band-pass filter ET540/30 m (Chroma) for the detection of fYFP or dYFP in Frame Segment 2. This configuration enabled sequential detection of four fluorescent signals (fCFP, fYFP, dYFP, and mScarlet) using two split detection windows (Frame Segment 1 and 2) within a single camera (ORCA-Flash4.0).

Although fCFP images were routinely recorded, we found that the UV-curable resin (Jelly Nail) applied to the skull surface to maintain cranial transparency emitted substantial fluorescence at ~473 nm when excited with 430 nm Royal Blue light, even after the resin was fully cured. Therefore, fCFP images were not used for further analysis. Pyruvate and ATP concentration signals were analyzed not by the conventional ratiometric method between fCFP and fYFP, but by the difference method (dYFP − fYFP for PYRS and fYFP − dYFP for ATeam, respectively)^3,36^.

The magnification of the MVX10 microscope was set to ×0.63. The camera was controlled using HCImageLive software (Hamamatsu). The images were split into two segments (Frame Segment 1 and 2) according to the emission wavelengths, using the dichroic mirrors and filters mounted on the microscope and the W-VIEW GEMINI image-splitting optics, and projected onto the two halves of the CMOS sensor of a single imaging camera. The resolution of the combined image (including both Frame Segment 1 and 2) was 256 × 512 pixels; thus, each frame segment occupied 256 × 256 pixels. Frame acquisition timing, excitation light timing, and synchronization of imaging with electrophysiological recordings were controlled using waveform generators (AWG-50).

#### Configuration 1

For dYFP (from either PYRS or ATeam) and mScarlet imaging, the camera frame exposure time was set to 89 ms for both Frame Segment 1 (mScarlet image) and Frame Segment 2 (dYFP image). Images including both frame segments were recorded simultaneously at a sampling frequency of 10 Hz. Excitation lights were delivered alternately at 100 ms intervals with 89 ms durations, starting with Cyan and followed by Green/Yellow excitation. Frame capture timing was synchronized with excitation light delivery, both controlled by the AWG-50 waveform generator. Blue and Green/Yellow excitation resulted in dYFP and mScarlet images in Frame Segments 2 and 1, respectively. As these images were acquired alternately, the effective sampling frequency for each fluorescence image (dYFP and mScarlet) was 5 Hz.

#### Configuration 2

dYFP images can be considered as shadow images of the blood vessels. Therefore, dYFP fluorescence intensity primarily reflects the inverse of brain blood volume dynamics, although potential contributions from cytosolic pH fluctuations affecting YFP fluorescence should be taken into account. In this configuration, only Cyan excitation light was delivered, and only dYFP images in Frame Segment 2 were analyzed. The excitation light and frame exposure were synchronized, each with a duration of 39 ms, at a sampling frequency of 20 Hz. Since only a single excitation wavelength was used, dYFP images were acquired at this frequency.

#### Configuration 3

For ATP or pyruvate imaging, the camera frame exposure time was set to 239 ms for both Frame Segment 1 (fCFP image) and Frame Segment 2 (fYFP or dYFP image). Images including both frame segments were recorded simultaneously at a sampling frequency of 4 Hz. Excitation lights were delivered alternately at 250 ms intervals, with a 239 ms duration for Royal Blue excitation and a 39 ms duration for Cyan excitation. Thus, for the dYFP image, Cyan excitation was applied for only 39 ms within each 239 ms frame exposure, with the remaining exposure time occurring in the dark. Royal Blue excitation resulted in fCFP and fYFP images in Frame Segments 1 and 2, respectively, while Cyan excitation produced the dYFP image in Frame Segment 2. Since Royal Blue and Cyan excitation were delivered alternately, the effective sampling frequency for each of the three images (fCFP, fYFP, and dYFP) was 2 Hz.

### Extracting molecule concentration dynamics from FRET imaging in vivo

Various FRET-based sensors have been developed, but their specifications are typically characterized only in vitro. In FRET-based sensors with increasing FRET efficiency, using CFP as the donor and YFP as the acceptor, CFP fluorescence decreases while YFP fluorescence increases upon binding of the target molecule to the sensor protein. However, in live in vivo preparations, factors such as brain blood volume (BBV) dynamics and cytosolic pH fluctuations can substantially influence the detected fluorescence signals. As a result, situations in which both CFP and YFP signals either decrease or increase from baseline frequently occur, complicating interpretation of the target molecule dynamics.

Violet to yellow excitation and emission light are well absorbed by the blood within the vasculature. Therefore, when fluorescent proteins are diffusely expressed in brain cells, blood vessels that lack these proteins appear as dark shadows^36,37^. It has been shown that blood vessels spontaneously dilate and constrict (vasomotion) and change their diameter in response to nearby neuronal activity (functional hyperemia)^2^. Changes in vessel diameter lead to local brain blood volume (BBV) fluctuations, which in turn result in inverse changes in fluorescence signal intensity. However, for FRET-based sensors, if we assume that BBV changes similarly affect both CFP and YFP fluorescence, calculating the ratio of YFP to CFP fluorescence (Y/C ratio) can compensate for these BBV dynamics artifacts. This approach takes advantage of the multi-color fluorescence imaging properties of FRET-based sensors and has been widely used as a standard correction method.

However, CFP and YFP differ in their sensitivity to pH. Thus, fluctuations in local pH near the sensors can also affect fluorescence detection. For example, YFP fluorescence is highly sensitive to pH changes near neutral pH and is strongly quenched under mildly acidic conditions, whereas CFP fluorescence remains relatively stable. Consequently, when cytosolic pH decreases, YFP fluorescence declines while CFP fluorescence remains largely unaffected, resulting in a reduction of the Y/C ratio. If only the Y/C ratio is analyzed, such changes may be misinterpreted as reflecting a decrease in the target molecule concentration rather than a pH-dependent effect.

To address this issue, we developed a new correction method as an alternative to the Y/C ratio. This method involves acquiring two types of signals: YFP fluorescence obtained by direct excitation of YFP (dYFP), and YFP fluorescence generated via FRET from an excited CFP donor (fYFP). Since dYFP is not dependent on FRET, its fluorescence intensity does not reflect changes in the target molecule’s concentration but rather only environmental changes such as pH and BBV dynamics. In contrast, fYFP is affected by both the FRET efficiency changes dependent on the target molecule’s concentration and the same environmental changes as dYFP. Therefore, by subtracting dYFP from fYFP, it is possible to cancel out artifacts originating from environmental changes and extract the true signal representing the fluctuations of the target molecule. In addition, we found that the UV-cured resin exhibits high fluorescence in the CFP emission range with Royal Blue excitation; fCFP (FRET influenced CFP fluorescence) data was not readily usable for analysis. Thus, the difference method that uses only fYFP and dYFP fluorescence data was especially ideal in our imaging configuration.

However, even for the difference method, an assumption needs to be introduced to extract the target molecule dynamics. We assumed that during NREM sleep, the concentration of the target molecule was relatively stable and the fluctuations observed in fYFP and dYFP primarily rise from the fluctuations in the environmental changes (BBV and/or cytosolic pH). We also needed to assume that these environmental changes affect fYFP and dYFP similarly. In accordance with these assumptions, the fluorescence waveform of fYFP and dYFP was found to be almost perfectly parallel during NREM sleep. The absolute amplitudes of the signals are affected by the power of two excitation lights (Royal Blue and Cyan) and the gain of the CMOS sensor output relative to the light intensity, therefore, the amplitudes of fYFP and dYFP were adjusted so that the two signals matched nearly completely during the NREM sleep period. Specifically, we used the signal from NREM sleep period to model the relationship between the two signals and create a signal (scaled dYFP) that models the artifact component (i.e. pH and BBV dynamics) contained within fYFP.

The detailed procedure for calculating fluctuations in the concentration of the target molecule is as follows. First, a Gaussian filter was applied along the temporal axis (σ = 5 frames) to the acquired fYFP and dYFP image sets to reduce pixel-level noise. Next, the NREM sleep period, defined as the interval from 300 to 100 s before the onset of REM sleep, was identified within the image sets. For each corresponding pixel, dYFP intensity was plotted against fYFP intensity, and a linear regression was fitted. The resulting slope and intercept were then used to scale the dYFP signal. This “scaled dYFP” signal closely overlaid the fYFP signal during the NREM period used for regression fitting, indicating that it successfully captured shared components, such as environmental artifacts. The scaled dYFP signal thus represents the artifact component (e.g., changes in BBV and cytosolic pH) embedded within the fYFP signal. The baseline fluorescence level for both the scaled dYFP and fYFP signals was defined as the mean of the upper 75% of signal values during the NREM period. The difference between the signal and this baseline (ΔF) was calculated and normalized by the baseline to derive ΔF/F values for both channels. The ΔF/F of the scaled dYFP signal was then subtracted from that of the fYFP signal to yield a corrected signal representing cytosolic ATP concentration fluctuations in ATeam imaging. For PYRS imaging, where FRET efficiency decreases upon pyruvate binding, the corrected value was inverted. In both imaging modalities, deviations from the linear (dYFP – fYFP) relationship were observed during the transition to REM sleep, suggesting that changes in the target molecule concentration occurred. Whether the fluctuation of target molecule concentration also occurs in complete sync with the environmental factor (BBV and cytosolic pH) fluctuations during NREM sleep period remains unclear.

It should be noted that the (fYFP – dYFP) linear relationship was established during NREM sleep, whose hemodynamic fluctuations do not fully span the larger BBV changes encountered during REM. Thus, its application to REM involves an extrapolation. However, this interpretation is supported by the observation that SNP-induced vasodilation, which produces large BBV increases, results in deviations in the opposite direction (Supplementary Fig. 6). This opposite-direction response argues against a simple scaling artifact arising from the possible non-linear hemoglobin absorption at high blood volumes. Residual analysis further indicated that the deviation between fYFP and scaled dYFP during REM was not explained by BBV magnitude, but instead exhibited a consistent shift across the observed range. Together, these observations suggest that the REM-associated deviation between fYFP and scaled dYFP reflects a genuine change in the target molecule rather than a BBV-dependent nonlinearity.

### Brain registration and regional map generation

Due to variations in the orientation and positioning of each mouse brain relative to the microscope’s field of view, as well as inter-individual differences in brain dimensions, image registration was necessary to enable comparisons across different video datasets. All videos were aligned to a common reference, the Allen Mouse Brain Atlas, to ensure consistency in identifying and comparing brain regions^77^.

The atlas was first converted into a two-dimensional top-down view. Posterior and lateral cortical regions were manually trimmed to match the typical imaging field of view. The resulting reference atlas was then segmented into the following anatomical regions: secondary motor cortex (MOs), primary motor cortex (MOp), upper limb somatosensory cortex (SSu), lower limb somatosensory cortex (SSl), barrel field somatosensory cortex (SSb), posterior parietal cortex (PPC), auditory cortex (Aud), retrosplenial cortex (RSC), and primary visual cortex (V1). The auditory cortex, being relatively small, was excluded from further quantitative analyses. For imaging experiments involving ATP and pyruvate indicators, a modified version of the atlas was used in which the anterior cingulate area and medial portion of the RSC were slightly reduced.

Landmark-based registration was performed using MesoNet, a DeepLabCut-based pretrained deep neural network designed for automatic annotation of cortical landmarks^78^. In this study, bregma and lambda (corresponding to the 5th and 6th landmarks in the original MesoNet implementation) were automatically annotated using MesoNet. In contrast, the left and right frontal poles (2nd and 8th landmarks) were manually annotated. Using these four landmarks, a perspective transformation was applied to register each experimental video to the atlas space.

### Estimation of FIR-BRF (the finite-impulse-response brain-blood-volume response function)

To characterize the temporal relationship between ECoG theta-band power and brain blood volume (BBV) fluctuations during NREM sleep, we derived a data-driven response kernel termed the finite-impulse-response brain-blood-volume response function (FIR-BRF). This kernel captures how past neuronal activity contributes to current hemodynamic signals, effectively modeling the transformation from neuronal dynamics to blood volume changes. Unlike the canonical double-gamma hemodynamic response function (HRF), which has a fixed analytic form, the FIR-BRF makes no prior assumption about the shape of the response and is instead estimated directly from each NREM episode by multiple linear regression.

The input signal x(t) was the theta-band (6–9 Hz) power of the ECoG, originally sampled at 2000 Hz. The output signal y(t) was the inverted direct YFP signal (idYFP; ΔF/F), sampled at 2 Hz. To match the temporal resolution of the output signal, the theta-band power was downsampled to 2 Hz prior to regression. Both x(t) and y(t) were then z-scored within each NREM episode before regression. The FIR-BRF h(τ) was defined as a finite-duration kernel spanning 0–20 s (L = 40 samples at 2 Hz) that, when convolved with x(t), yields the predicted idYFP signal:

$$\hat{y}\left(t\right)={\sum }_{\tau =0}^{L-1}h(\tau )x(t-\tau )$$

The kernel coefficients h(τ) were estimated by minimizing the mean-squared error between the predicted and observed idYFP signals. Regression was performed independently for each NREM sleep episode, without regularization, yielding one FIR-BRF per episode (13 FIR-BRFs in total from 13 episodes across 3 mice). The peak latency of each estimated FIR-BRF, defined as the time lag τ at which h(τ) attained its maximum value (argmax), was used to characterize the temporal offset between theta-band activity and BBV changes (Fig. 1l).

### Assessment of regional similarity by hierarchical clustering of signals

Regional similarities in the dYFP signals across the cortex were assessed as shown in Fig. 2. The time-series image stack was segmented into NREM and REM sleep periods. For each segment, the ΔF/F images of dYFP signals containing the left hemisphere were divided into a grid consisting of 16 × 32 tiles. Among these, 249 tiles overlapped with the imaged brain region and were included in the analysis.

Within each sleep segment (NREM or REM), the ΔF/F dYFP signal time series from one tile was compared with that of every other tile in the brain region by calculating the correlation coefficient between their respective signals. Repeating this procedure for all tile pairs yielded a square matrix of pairwise correlation coefficients. Each row of this matrix represents a correlation vector: the pattern of similarity between one tile and all others. To assess the similarity between the tiles based on their overall correlation profiles, pairwise distances between these vectors were computed using Ward’s hierarchical clustering method. Clustering was then performed by applying a distance threshold chosen according to the desired number of clusters. Although similarity between tiles could also be evaluated directly using the raw ΔF/F time series, more robust and stable clustering results were obtained by using the correlation coefficient vectors as features for comparison.

### Temporal delay-based clustering for spatiotemporal similarity mapping

Assessing regional similarity solely by clustering correlation coefficient vectors can be misleading for two main reasons. First, a large but slow decrease in the dYFP signal was observed across all tiles at the onset of REM sleep. This global signal fluctuation can mask local dynamics and should be treated separately from higher-frequency components. Second, correlation coefficients do not account for temporal offsets between signals from different tiles. For example, if the signal in one tile consistently mirrors that of another with a fixed time delay, their correlation coefficient may still be low, misleadingly indicating weak similarity. Conventional functional connectivity analyses often rely on zero-lag correlation coefficients. However, in the brain, neural activity or blood flow changes in one region can propagate to others with finite time lags, potentially resulting in strong delayed correlations. To capture such spatiotemporal propagation of blood flow-related activity, this study performed a functional connectivity analysis that incorporates time lags (Fig. 4, Supplementary Fig. 2). These analyses were based on the high-frequency components of the dYFP signal, which better reflect localized and dynamic interactions.

For these analyses, the ΔF/F images of dYFP signals from the left hemisphere were first divided into a grid consisting of 64 × 128 tiles, of which 1065 tiles overlapped with the imaged brain region. The ΔF/F time series from each tile was band-pass filtered between 0.05 and 0.2 Hz to remove slow signal fluctuations, which were especially prominent during REM sleep. Frequency components above 0.2 Hz, which may have included instrumental noise, were excluded from further analysis.

Lagged cross-correlation functions were then computed for all possible pairs of tiles within the imaged brain region. The lagged cross-correlation function is defined as follows:

$${R}_{x1x2}(\tau )=\frac{1}{{\sigma }_{x1}{\sigma }_{x2}}\frac{1}{T}\int {x}_{1}(t)\cdot {x}_{2}(t+\tau )\,{dt}$$

where ${x}_{1}$ represents the signal from the seed tile (or the region of interest, ROI) and ${x}_{2}$ represents the time series signal from another tile. ${\sigma }_{x1}$ and ${\sigma }_{x2}$ denote the standard deviations of ${x}_{1}$ and ${x}_{2}$, respectively, and $T$ is the duration of overlap between the two signals. $\tau$ represents the time lag. Notably, ${R}_{x1x2}(0)$ corresponds to the conventional Pearson correlation coefficient. To accelerate computation, the convolution theorem was applied, which states that convolution in the time domain is equivalent to multiplication in the frequency domain. Accordingly, the cross-correlation function was computed using Fourier transforms as follows:

$${R}_{x1x2}(\tau )={{{{\mathscr{F}}}}}^{-1}\left\{{X}_{1}(f)\cdot {X}_{2}^{* }(f)\right\}$$

where ${X}_{1}$ and ${X}_{2}$ represent the frequency spectra obtained by applying the Fourier transform to ${x}_{1}$ and ${x}_{2}$, respectively. The symbol * denotes the complex conjugate, and ${{{{\mathscr{F}}}}}^{-1}$ indicates the inverse Fourier transform. From the resulting cross-correlation function, the time lag corresponding to the peak correlation was defined as the temporal delay (TD), and the correlation coefficient at that time was defined as the temporally delayed cross-correlation (XC_TD_). A TD matrix and an XC_TD_ matrix were constructed based on these values. By definition, the TD matrix is antisymmetric, while the XC_TD_ matrix is symmetric.

### Slow dYFP signal-based analysis of the REM sleep onset delays

To analyze the temporal dynamics of slow dYFP signals during REM sleep onset, ΔF/F images from each tile within the anterior and posterior regions of interest (ROIs) were averaged and subjected to low-pass filtering at 0.05 Hz, thereby isolating the low-frequency components of the signal. The resulting signals were normalized such that the average value during the 30 s immediately preceding REM sleep (defined as the pre-REM sleep period) was set to 1, and the minimum value during REM sleep was set to 0. For each ROI, the elapsed time from REM sleep onset, determined by EEG and EMG recordings, to the point at which the normalized signal decreased to half of its initial value was calculated. The time difference (Δt) between the anterior and posterior ROIs was then computed (Fig. 3b, c).

A similar analysis was performed at the tile level. First, the signal from each tile was smoothed and normalized, and its half-decay time was determined using the same method. The tile that exhibited the earliest decrease in signal (referred to as the “earliest” tile in Fig. 3d) was identified. A timing map was then generated by calculating the relative time difference between this earliest tile and each of the other tiles.

### Extraction of spatiotemporal motifs in dYFP signals

Analysis of the fast (0.05–0.2 Hz) temporal component of the dYFP signal revealed several recurring spatiotemporal patterns (Fig. 5). These patterns likely represent distinct motifs of cerebral blood volume fluctuations, possibly linked to repeated energy demands arising from neural information processing during NREM sleep, REM sleep, and wakefulness. To extract these spatiotemporal motifs of cortical vasomotion activity, we employed the seqNMF algorithm (https://github.com/ContextLab/seqnmf), which is based on non-negative matrix factorization with additional regularization terms. This method enables the identification of repeated spatiotemporal patterns within two-dimensional data by employing non-negative matrix factorization with penalty terms. Specifically, the data matrix X (N pixels × T time points) is factorized into a motif matrix W (N pixels × L time points × K motifs) and a temporal weight matrix H (K motifs × T time points).

Initially, each ΔF/F video was divided into a grid of 64 × 128 tiles, with 1065 tiles overlapping the imaged brain region. To reduce data size, the video sampling rate was downsampled from 20 Hz to 4 Hz. A band-pass filter (0.05–0.2 Hz) was applied to the dYFP signal in each tile, followed by the Hilbert transform. The absolute values of the resulting analytic signals were used to compute upper envelope signals, representing the instantaneous amplitude of cerebral blood volume fluctuations. Since envelope amplitudes tend to be lower during REM sleep, the temporal weights during this state were also biased toward lower values, complicating direct comparison of motif occurrence intensities across brain states. To address this, we applied min–max normalization to the envelope signals from each video frame, rescaling signal intensity to a 0–1 range. Additionally, frames were thresholded at 0.3 to suppress noise and stabilize motif extraction. Each frame was then vectorized, converting the data into a tiles × time points matrix (Fig. 5a).

The following parameters were used for motif extraction: number of motifs *K* = 15, maximum motif duration *L* = 24 (corresponding to 6 s at 4 fps), number of iterations = 100, sparsity and orthogonality penalty *λ* = 0.0001, and temporal orthogonality parameter λOrthH = 1. The extracted motifs were subsequently grouped into 10 categories based on their spatial features, using visual inspection. The motif duration parameter L (6 s) was chosen based on the frequency content of the input signals and the stability of extracted motifs. The dYFP signal was band-pass filtered between 0.05 and 0.2 Hz prior to envelope extraction. The power spectrum of this envelope signal showed that its frequency components were broadly distributed across periods longer than 6 s (< 0.16 Hz), suggesting that fast-component oscillations may contain spatiotemporal patterns at various time scales. We aimed to identify the longest motif duration that could be robustly extracted across recordings. When longer windows were tested (e.g., 10 s), the extracted motifs were not stable across recordings. Accordingly, a 6-second window was selected as the longest duration that allowed consistent identification of spatiotemporal motifs and represents a minimal repeating unit of the underlying dynamics.

### Detection criteria for ECoG and fluorescence signal onset

REM sleep onset was defined as the time point at which the ECoG theta/delta power ratio exceeded a predefined threshold. Around this time, deflections were observed in both ECoG signals and fluorescence signals from PYRS and ATeam. The following signal changes were temporally aligned to the REM sleep onset and analyzed: (1) an increase in ECoG theta-band power (6–9 Hz), (2) a decrease in dYFP signal intensity, (3) an increase in cytosolic pyruvate levels in astrocytes, and (4) a decrease in cytosolic ATP levels in neurons (Fig. 7). For all electrophysiological and fluorescence signals, onset detection was referenced to the REM sleep onset defined by the theta/delta power ratio. For each signal, the onset was defined as the threshold-crossing event that occurred closest in time to the REM onset.

To determine the onset of theta-band power increase, the ECoG theta-band power (6–9 Hz) time series was smoothed using a 10-second moving average. The mean and standard deviation of theta-band power during the NREM sleep period were then computed. The onset of theta-band power increase was defined as the earliest time point at which the smoothed signal exceeded the mean plus one standard deviation, within proximity to the identified REM sleep onset.

For dYFP, the baseline signal intensity was calculated from the NREM sleep period. The onset of the signal decrease was defined as the time point when the dYFP intensity fell more than 1% below this baseline. Because such decreases may also occur outside REM transitions, only the instance closest to REM onset was considered the relevant dYFP onset. Similarly, thresholds for pyruvate and ATP signals were set at a 0.5% increase (pyruvate) and a 0.5% decrease (ATP) from their respective NREM baselines. As with the dYFP signal, the onset was defined as the threshold-crossing point closest to REM onset.

### Randomization

This study used a within-animal design in which each animal served as its own control across vigilance states (NREM, REM, and WAKE) and before and after pharmacological vasodilation. Because animals were not allocated into separate control and treatment groups, randomization was not applicable and no randomization sequence was generated. All recordings were performed on the same imaging setup to avoid equipment- or location-related variability.

### Statistical analysis

All statistical analyses were conducted using custom-written scripts in Python. Formal tests of normality were not performed given the limited sample sizes; parametric tests were applied under the assumption of normality, and exact *p*-values are reported throughout. A significance threshold of p < 0.05 was applied throughout. Effect sizes are reported as Cohen’s *d*. In this study, biological replicates are defined as individual animals. Technical replicates represent repeated measurements or trials (e.g., sleep episodes) from the same animal. Data are reported as mean ± standard error of the mean (SEM).

For comparisons between two related groups, two-tailed paired t-tests were employed (Fig. 3b; Fig. 4e, h; Supplementary Fig. 2f, g; Supplementary Fig. 5c, d). To test whether the mean of a single sample differed from a defined value (typically zero), one-sample t-tests were conducted (Fig. 4k; Fig. 6g, h; Fig. 7c, d). For comparisons involving more than two groups, one-way analysis of variance (ANOVA) followed by Tukey’s post-hoc test was used (Fig. 7c, d; Supplementary Fig. 4c). To examine the interaction between signal type (pyruvate vs. ATP) and time point (Post1 vs. Post2), a two-way mixed-design ANOVA was performed (Fig. 6g, h). When multiple comparisons were required, *p*-values were adjusted using the Holm correction method to control for family-wise error rate.

### Reporting summary

Further information on research design is available in the Nature Portfolio Reporting Summary linked to this article.

## Supplementary information

Supplementary Information

Supplementary Movie 1

Supplementary Movie 2

Supplementary Movie 3

Supplementary Movie 4

Supplementary Movie 5

Supplementary Data 1

Description of Additional Supplementary files

Reporting Summary

Transparent Peer Review file

## Data Availability

The numerical source data underlying all graphs presented in the main and Supplementary Figs. are provided with this paper as a Supplementary Data file. Raw data supporting the findings of this study are available from the corresponding author on reasonable request.

## Code availability

Custom-written Python codes are available from the corresponding authors upon reasonable request.
