# Supplementary material for: Energy paradox in REM sleep: balancing supply and consumption in brain metabolism
Source: Commun Biol. 2026 Jul 27;9:979. doi: 10.1038/s42003-026-10646-6 (PMC13408869; doi:10.1038/s42003-026-10646-6)
Supplement: Supplementary file 1 — Supplementary Information [file 42003_2026_10646_MOESM1_ESM.pdf]

Supplementary Information:

Energy paradox in REM sleep: balancing supply and consumption in brain metabolism

Yusuke Takahashi[1], Yoko Ikoma[1], Ko Matsui[1, 2 *]

[1] *Super-network Brain Physiology, Graduate School of Life Sciences, Tohoku University, Sendai 980-8577 Japan*

[2] *Super-network Brain Physiology, Graduate School of Medicine, Tohoku University, Sendai 980-8577 Japan*

[*] Corresponding author.

E-mail: matsui@tohoku.ac.jp (K.M.)

Contents:

Supplementary Figures 1 – 10

Description of Supplementary Movies 1 – 5

Supplementary Figures

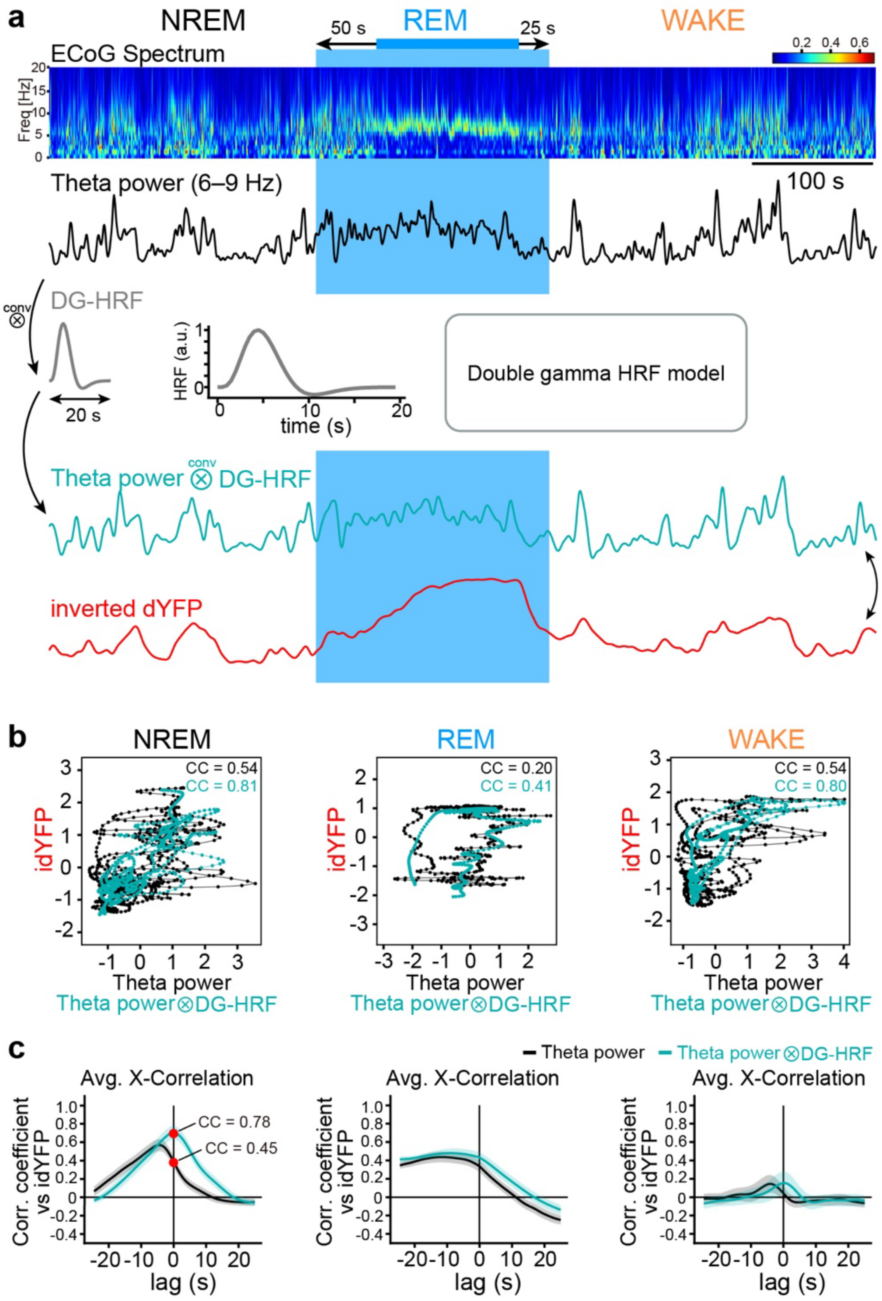

Supplementary Fig. 1: Theta-band power closely tracks inverted dYFP (idYFP) signal during NREM sleep. a, Representative ECoG spectrogram, raw theta-band power (6–9 Hz) of ECoG, HRF-convolved theta-band power, and idYFP signal across sleep-wake states. Theta-band power was convolved with a parameter-optimized double-gamma hemodynamic response function (DG-HRF; inset), yielding a signal more closely aligned with idYFP. All traces are z-scored. The cyan shading denotes the REM analysis window, defined as the classically defined

REM episode (blue bar) plus a peri-REM window (50 s before and 25 s after). **b,** Scatterplots showing correlations of idYFP with raw theta-band power (black) and DG-HRF-convolved theta-band power (cyan) during NREM, REM, and WAKE periods. Values indicate Pearson correlation coefficients (CC). **c,** Cross-correlation analysis (mean ± SEM, 14 episodes from n = 3 mice) between idYFP and raw theta-band power (black) or theta-band power convolved with DG-HRF (cyan) for each sleep-wake state. Red circles mark coefficients at zero-time lag (CC). Convolution with the HRF eliminates the ~4 s temporal lag between the theta power and idYFP and markedly improved correlation, particularly during NREM sleep.

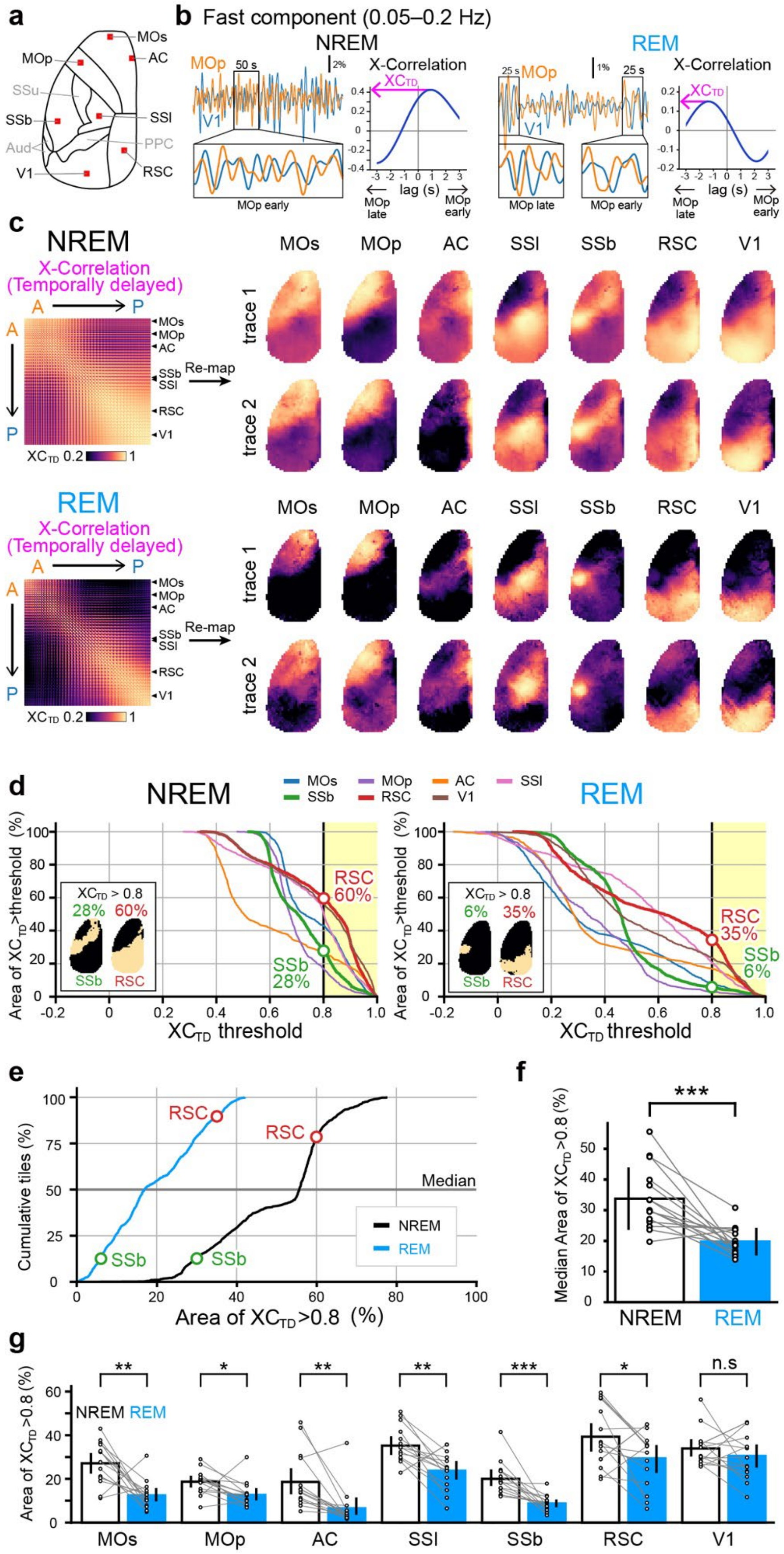

Fast component (0.05–0.2 Hz)
NREM
REM
X-Correlation
X-Correlation (Temporally delayed)
Re-map
Area of XC$_{TD}$>threshold (%)
XC$_{TD}$ threshold
Cumulative tiles (%)
Area of XC$_{TD}$>0.8 (%)
Median Area of XC$_{TD}$>0.8 (%)

Supplementary Fig. 2: The fast component of brain blood volume (BBV) fluctuations becomes spatially localized during REM sleep. a, Locations of representative tiles (red) in each brain region (MOs, secondary motor cortex; MOp, primary motor cortex; AC, anterior cingulate area; SSu, somatosensory upper limb cortex; SSl, somatosensory lower limb cortex; SSb, somatosensory barrel cortex; PPC, posterior parietal cortex; RSC, retrosplenial cortex; V1, primary visual cortex). **b,** Examples of lagged cross-correlation analysis. dYFP signals (0.05–0.2 Hz band-pass) from MOp and V1 tiles were analyzed, and the maximum cross-correlation coefficient across time lags was defined as temporally delayed cross-correlation (XC_{TD}). **c,** XC_{TD} matrices for all tile pairs during NREM and REM sleep (left). Rows represent XC_{TD} values between one seed tile and all others, ordered from anterior–lateral to posterior–medial. Vectors from selected seed tiles (arrows) were remapped onto cortical maps (right). **d,** Percentage of cortical area exceeding different XC_{TD} thresholds for each seed tile is plotted as a function of threshold. During REM sleep, the area with $XC_{TD} > 0.8$ were restricted (RSC 35%, SSb 6%), whereas during NREM sleep they were broader (RSC 60%, SSb 28%). Insets show cortical distribution of $XC_{TD} > 0.8$ for RSC and SSb seeds. Highly correlated fast-component fluctuations became spatially localized in REM. **e,** Cumulative distributions of cortical tiles sorted by area with $XC_{TD} > 0.8$ (blue, REM; black, NREM). The RSC seed during REM sleep (35%) ranked in the top 10% of all tiles. **f,** Median area with $XC_{TD} > 0.8$ was significantly larger in NREM than REM ($p = 0.00036$, 15 episodes from $n = 3$ mice, paired t-test; ***$p < 0.001$; error bars, SEM). **g,** Comparison of areas with $XC_{TD} > 0.8$ between sleep stages for each region. Significant reductions during REM were observed in all regions except V1 (MOs, $p = 0.0011$; MOp, $p = 0.015$; AC, $p = 0.0022$; SSl, $p = 0.0036$; SSb, $p = 0.00036$; RSC, $p = 0.042$; V1, $p = 0.33$; 15 episodes from $n = 3$ mice, paired t-test; *$p < 0.05$, **$p < 0.01$, ***$p < 0.001$; error bars, SEM).

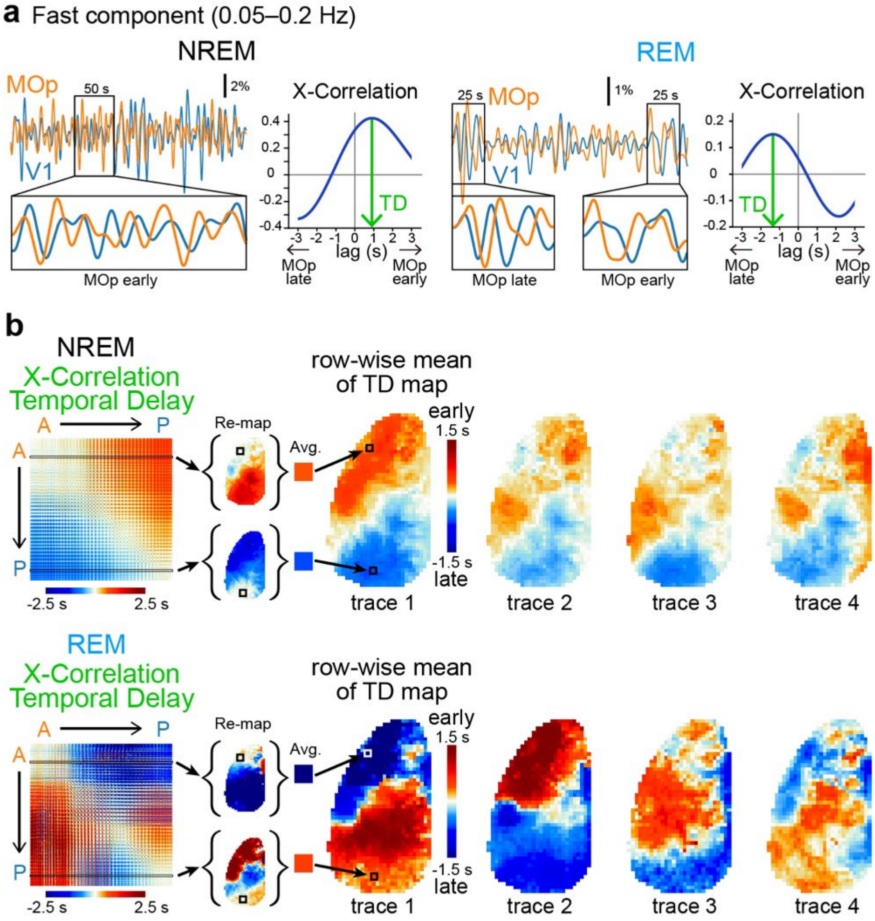

Supplementary Fig. 3: Temporal delays of fast-component dYFP correlations reveal stable propagation during NREM but variable patterns during REM sleep. a, Example of lagged cross-correlation analysis. Band-pass filtered dYFP signals (0.05–0.2 Hz) from MOp and V1 tiles were compared, and the time lag at peak cross-correlation was defined as the temporal delay (TD). **b,** TD matrices across all tile pairs during NREM and REM sleep. Each row shows TD values of one reference tile relative to all others, ordered from anterior–lateral to posterior–medial. Positive (orange) values indicate the reference tile leads; negative (blue) values indicate it lags. Each row was re-mapped onto a cortical map and averaged to generate "row-wise mean of TD maps", in which each tile reflects its average lead or lag relative to the entire cortex. Four representative examples are shown for each state. During NREM sleep, a consistent anterior-to-posterior propagation was evident, with anterior regions leading posterior regions by ~1 s. In contrast, REM sleep lacked such consistency, showing diverse and variable spatial patterns across trials.

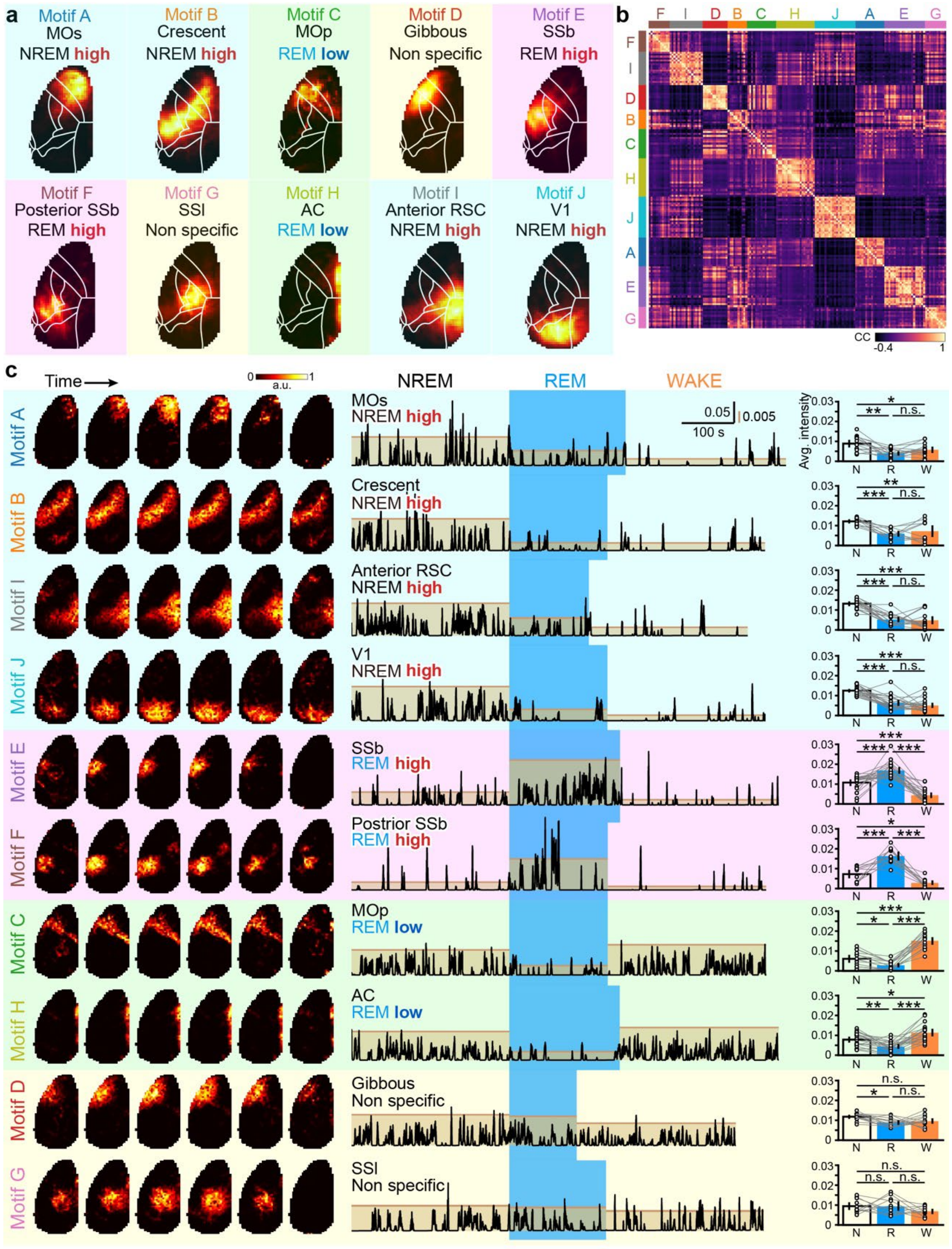

Supplementary Fig. 4: Identification of diverse spatiotemporal motifs of brain blood volume (BBV) dynamics using seqNMF. a, Average spatial patterns of 10 representative motif types (A–J) extracted from the fast component of the dYFP signal using seqNMF. Spatiotemporal motifs of 6 s in duration were extracted (225 motifs from 15 episodes from n = 3 mice) and manually

clustered based on spatial similarity. A total of 181 motifs were assigned to one of the 10 types:

Motif A (MOs): secondary motor cortex
Motif B (Crescent): crescent-shaped band spanning AC through SSb
Motif C (MOp): primary motor cortex
Motif D (Gibbous): gibbous, convex area centered on the anterior MOp
Motif E (SSb): barrel field somatosensory cortex
Motif F (Posterior SSb): posterior barrel field somatosensory cortex
Motif G (SSl): lower limb somatosensory cortex
Motif H (AC): anterior cingulate area
Motif I (Anterior RSC): anterior retrosplenial cortex
Motif J (V1): primary visual cortex

The remaining 44 motifs were classified as "Others". **b,** Correlation matrix of spatial similarity across the 181 classified motifs. Each motif was vectorized, and Pearson correlation coefficients were calculated. **c,** Representative examples of each motif type (left), their temporal weights across time (middle), and mean temporal weights across sleep-wake stages (NREM, REM, WAKE; right, error bars, SEM). Blue shading in the middle panel indicates the REM sleep period (duration varied across episodes). Distinct stage-dependent activity patterns emerged:

REM high: Motifs E (SSb), F (Posterior SSb), enhanced during REM
REM low: Motifs C (MOp), H (AC), suppressed during REM
NREM high: Motifs A (MOs), B (Crescent), I (Anterior RSC), J (V1), enhanced during NREM
Notably, temporal occurrence of occipital motifs (I, J) gradually increased during NREM and peaked just before REM onset.
Non specific: Motifs D (Gibbous), G (SSl), non-specific to any sleep-wake stage

Statistical comparisons (bar graphs) were performed using one-way ANOVA with Tukey's post-hoc test (p* < 0.05, **p < 0.01, ***p < 0.001).

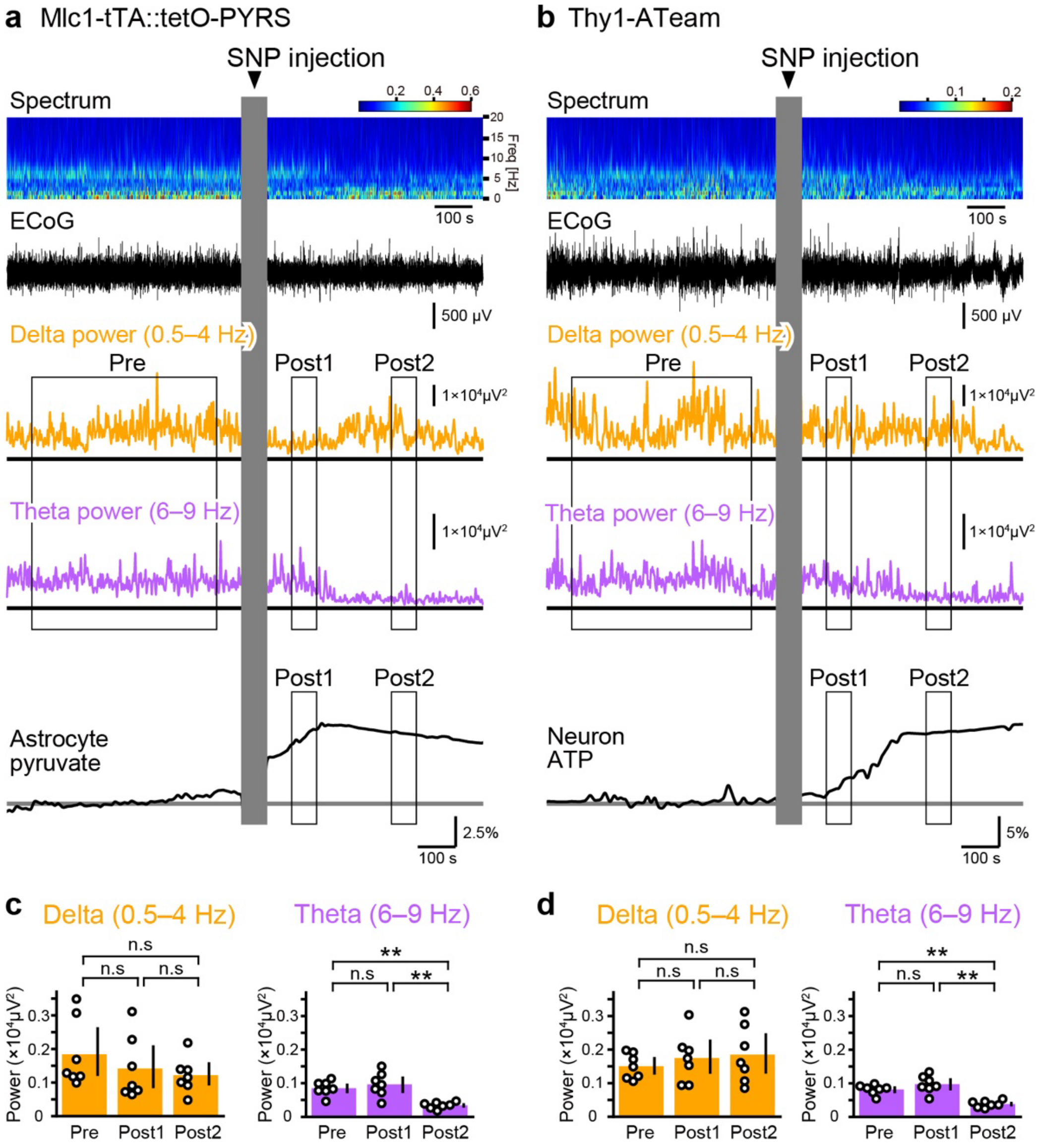

Supplementary Fig. 5: Effect of SNP administration on ECoG power. a, Representative recording from an Mlc1-tTA::tetO-PYRS mouse. Shown are the ECoG spectrogram, raw ECoG trace, delta-band power (0.5–4 Hz), theta-band power (6–9 Hz), and the astrocytic pyruvate signal [− (fYFP − dYFP)] before and after SNP administration. SNP was injected during the 60-s grey-shaded period. Black rectangles mark analysis windows: Pre (−9 to −1 min), Post1 (1–2 min), and Post2 (5–6 min). Signals within these windows were quantified for comparison in panels **c** and **d**. **b,** Representative recording from a Thy1-ATeam mouse. The bottom trace shows the neuronal ATP signal (fYFP − dYFP). **c, d,** Delta- and theta-band power during the Pre, Post1, and Post2 windows. Panel **c** summarizes astrocytic PYRS mice (7 episodes from $n = 3$ mice; error bars, SEM), and **d** summarizes neuronal ATeam mice (7 episodes from $n = 3$ mice; error bars, SEM). Delta power did not differ across windows in either mouse line (paired t-test with Holm

correction). Theta power was also unaffected by SNP injection during Post1 relative to Pre, but was significantly reduced in Post2 compared with both Pre and Post1 in both mouse lines (paired t-test with Holm correction, $^{**}p < 0.001$).

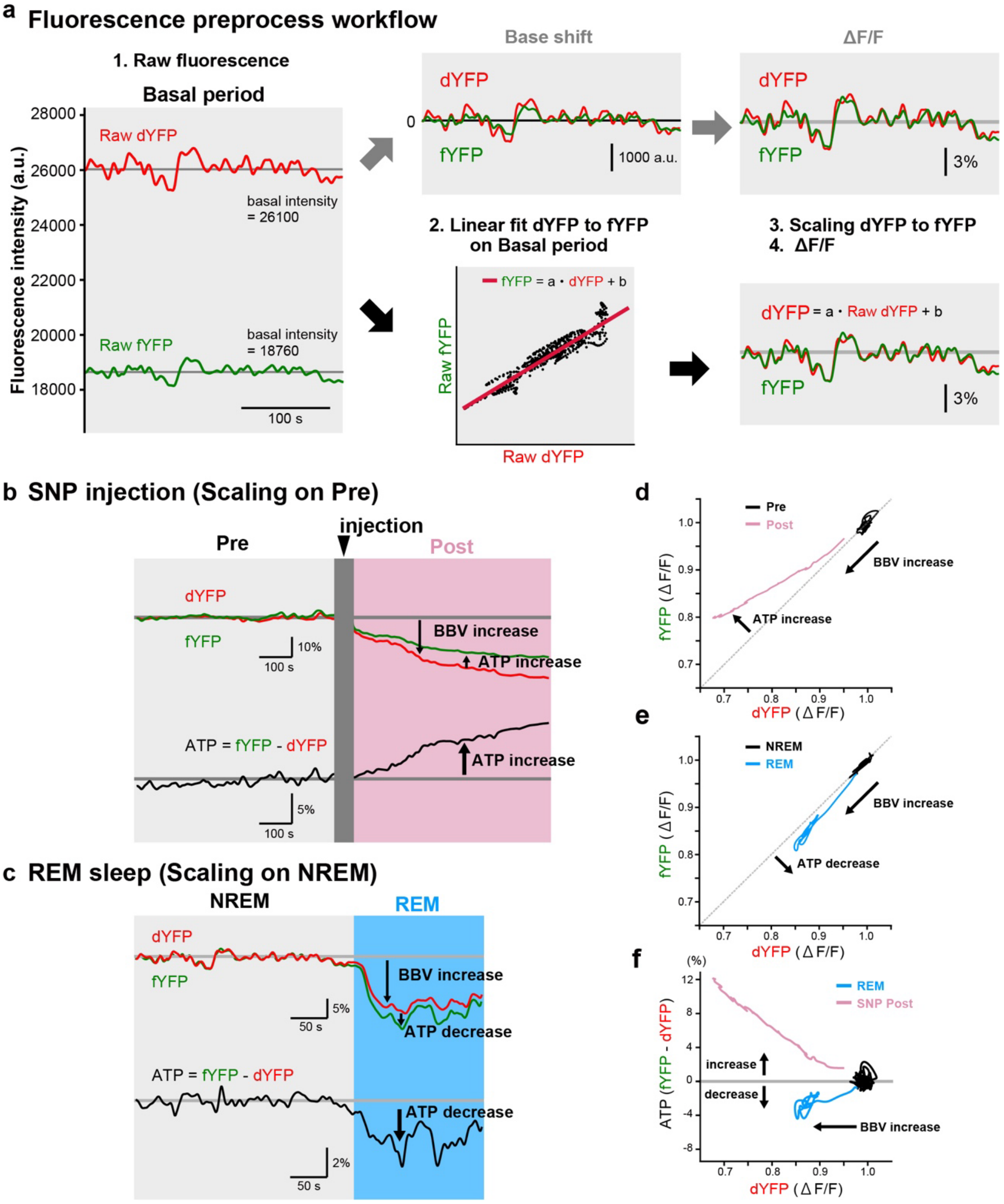

Supplementary Fig. 6: Preprocessing workflow for FRET sensor signals and validation of the linear scaling assumption using SNP-induced vasodilation and REM sleep.

a, Preprocessing workflow for extracting target metabolite signals from FRET sensor data. Raw fYFP (green; FRET YFP, excited via CFP) and raw dYFP (red; YFP directly excited at the YFP excitation wavelength) were acquired simultaneously and differ substantially in absolute intensity due to differences in excitation power and detector gain (left). Subtracting the basal intensity from each channel ("Base shift", top middle) leaves a residual offset between the two waveforms, and

direct ΔF/F normalization alone (top right) does not align them either. Raw dYFP was therefore scaled to raw fYFP by linear regression over a basal period (fYFP = a · dYFP + b, bottom middle), and the scaled dYFP was then expressed as ΔF/F (bottom right). The scaled dYFP closely overlaid fYFP during the basal period, indicating that environmental contributions (BBV and cytosolic pH) shared by both channels were captured by this scaling. **b,** SNP-induced vasodilation in a representative ATeam mouse (RSC ROI), with scaling on the pre-injection period. fYFP (green), scaled dYFP (red), and the ATP signal (fYFP − scaled dYFP, black) are shown. Both channels decreased after injection (BBV increase), but fYFP decreased less than scaled dYFP, yielding a positive ATP residual. **c,** NREM-to-REM transition in the same animal and ROI, with scaling on the NREM period (300–100 s before REM onset). Layout as in b. During REM, fYFP decreased more than scaled dYFP, yielding a negative ATP residual. **d,** fYFP versus scaled dYFP trajectory for the SNP experiment (pre, black; post, pink); the dashed line indicates the diagonal. The post-injection trajectory deviates upward from the diagonal, indicating an ATP increase concurrent with BBV increase. **e,** Same plot for the NREM-to-REM transition (NREM, black; REM, blue). The REM trajectory deviates downward from the diagonal, indicating an ATP decrease concurrent with BBV increase. **d,** Direct comparison of the ATP residual trajectories during REM (blue) and after SNP injection (pink) for the same ROI. The horizontal axis represents dYFP (ΔF/F) as an inverse proxy for BBV, with leftward motion indicating BBV increase. Both conditions involve a comparable BBV increase, yet the ATP residuals follow opposite directions. This bidirectional behavior is incompatible with a hemodynamic scaling artifact, which would predict a unique sign of fYFP minus scaled dYFP for a given direction of BBV change, and supports the interpretation that the residuals reflect genuine ATP dynamics.

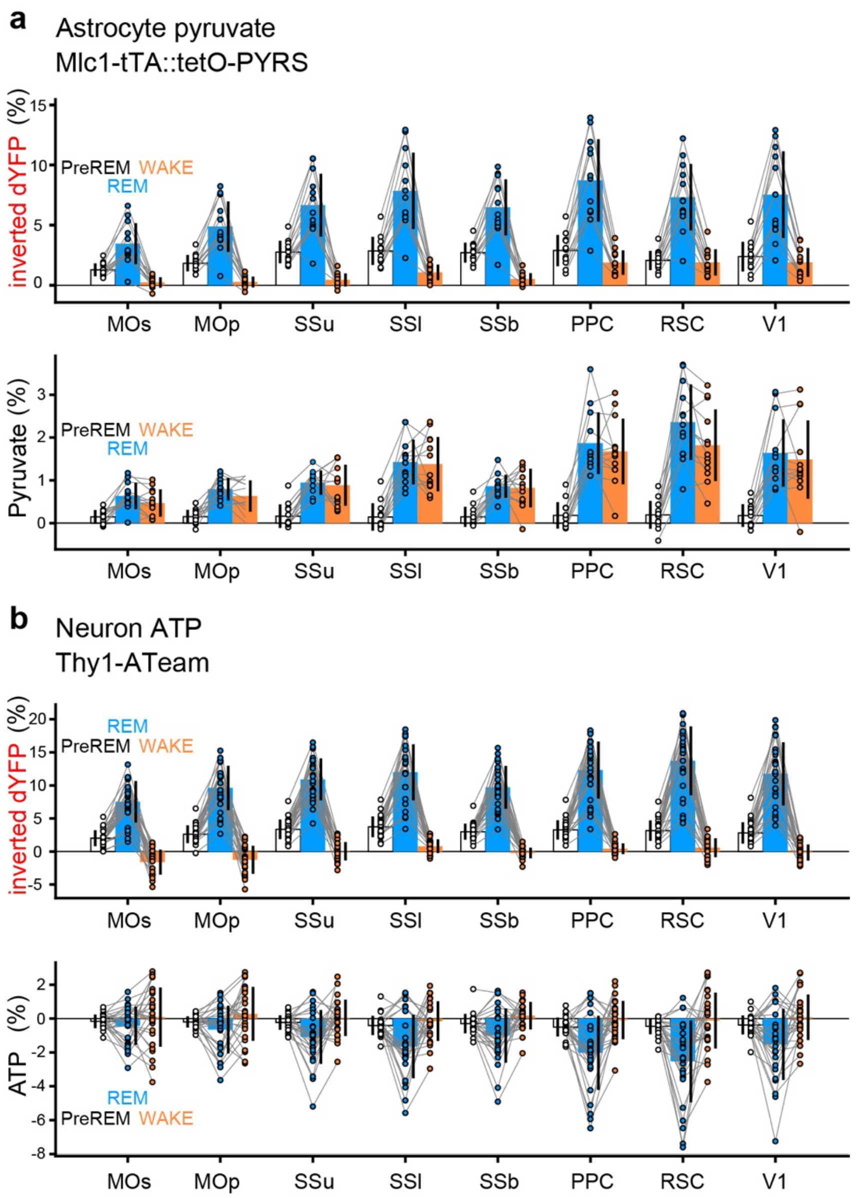

Supplementary Fig. 7: Region-wise comparison of astrocytic pyruvate, neuronal ATP, and idYFP signals across sleep–wake states.

a, Inverted dYFP (idYFP; top) and astrocytic pyruvate (bottom) signals from Mlc1-tTA::tetO-PYRS mice in eight cortical regions: secondary motor cortex (MOs), primary motor cortex (MOp), somatosensory upper limb cortex (SSu), somatosensory lower limb cortex (SSl), somatosensory barrel field cortex (SSb), posterior parietal cortex (PPC), retrosplenial cortex (RSC), and primary visual cortex (V1). Bars indicate mean values during PreREM (100 s before REM onset; white), REM (last 50 s before awakening; light blue), and WAKE (50 s after awakening; orange); error bars, SEM. Open circles represent individual episodes, and grey lines connect data points from the same episode. Astrocytic pyruvate increased during REM sleep across all eight regions, with

progressively larger increases toward posterior regions. **b,** Inverted dYFP (idYFP; top) and neuronal ATP (bottom) signals from Thy1-ATeam mice, plotted in the same format as in a. Neuronal ATP decreased during REM sleep across all eight regions, again with progressively larger decreases toward posterior regions, while idYFP showed a parallel anterior-to-posterior gradient of REM-associated increase. All values are absolute ΔF/F (%) (15 episodes from n = 3 mice for both a and b; error bars, SEM). These regional bar graphs correspond to the same dataset visualized as scatter plots in Fig. 8k, l.

	NREM	REM
Fast	Widespread and synchronized fluctuations<br>**Direction**: Anterior → Posterior (**~1 sec**)	Decreased and localized fluctuations<br>**Direction**: Unstable
Slow		Global increase in brain blood volume (BBV)<br>**Direction**: Posterior → Anterior (**~15 sec**)

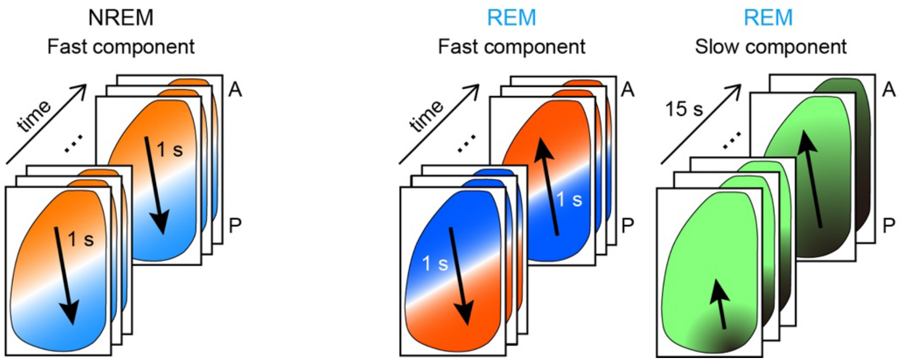

Supplementary Fig. 8: Summary of the spatiotemporal dynamics of brain blood volume (BBV) fluctuations in NREM and REM sleep. Schematic summary of the key spatiotemporal features of BBV fluctuations revealed in this study:

Fast component - NREM (left): The fast component (0.05–0.2 Hz) forms a stable propagating wave traveling from the anterior to posterior cortex in ~1 s.
Fast component - REM sleep (middle): Propagation of the fast component becomes fragmented and inconsistent, with variable flow direction alternating between anterior-to-posterior and posterior-to-anterior
Slow component - REM sleep (right): The slow component (< 0.05 Hz), emerging at REM onset, forms a large-scale wave propagating from posterior to anterior cortex over ~15 s.

These findings indicate that BBV dynamics are strongly state-dependent, adapting to the distinct information-processing and metabolic demands of NREM and REM sleep.

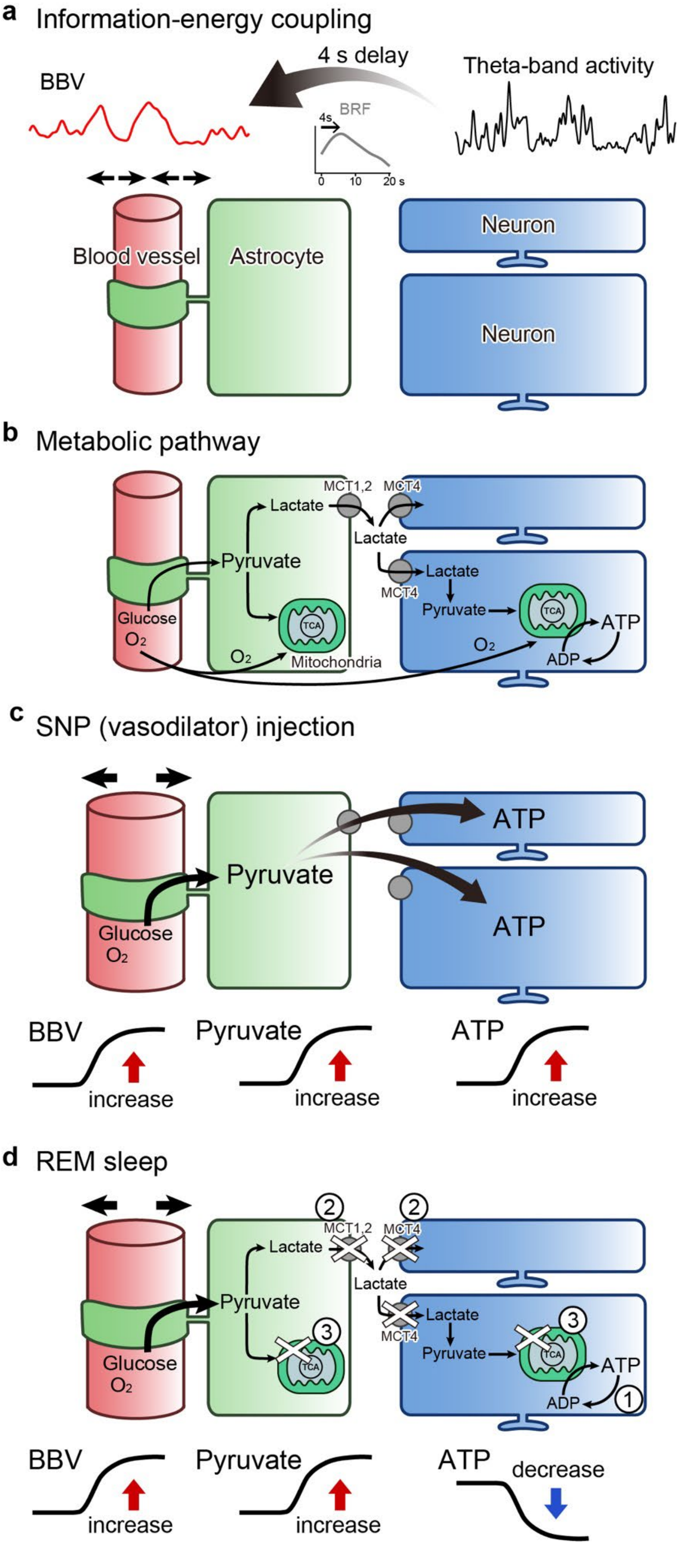
a
Information-energy coupling
4 s delay
Theta-band activity
BBV
BRF
4s
0
10
20 s
Blood vessel
Astrocyte
Neuron
Neuron
b
Metabolic pathway
MCT1,2
MCT4
Lactate
Lactate
Pyruvate
Lactate
MCT4
Pyruvate
Glucose
O2
TCA
TCA
ATP
O2
O2
Mitochondria
ADP
c
SNP (vasodilator) injection
ATP
Pyruvate
ATP
Glucose
O2
BBV
increase
Pyruvate
increase
ATP
increase
d
REM sleep
2
2
MCT1,2
MCT4
Lactate
Lactate
Pyruvate
3
Lactate
MCT4
Pyruvate
3
Glucose
O2
TCA
TCA
ATP
ADP
1
BBV
increase
Pyruvate
increase
ATP
decrease

Supplementary Fig. 9: Schematic of information-energy coupling. Summary of how neuronal information-processing states are coupled with metabolic pathways in the brain. **a NREM sleep:** Fluctuations in theta-band ECoG power closely correlated matched brain blood volume (BBV) dynamics. Convolving theta-band power with a simple brain-blood-volume response function (BRF), which created a delay of ~4 s, reproduced BBV fluctuations, indicating a predictive neurovascular coupling that optimizes energy supply to neuronal activity. **b Fundamental energy pathway:** Glucose from blood vessels is converted to pyruvate and lactate in astrocytes. Lactate is transported to neurons via monocarboxylate transporters (MCTs) and used for mitochondrial ATP production. Thus, increased blood volume is expected to raise intracellular energy availability. **c Effect of vasodilation:** Pharmacologically increasing BBV with SNP was accompanied by elevations in both astrocytic pyruvate and neuronal ATP, suggesting that enhanced blood supply can raise intracellular energy substrate levels. **d REM sleep paradox:** Despite elevated BBV and astrocytic pyruvate, neuronal ATP decreased. Possible explanations include:

(1) Increase energy consumption: Excessive ATP use during REM-specific information processing (e.g., memory reorganization).
(2) Restricted energy supply: Impaired lactate transfer from astrocytes to neurons, potentially via altered MCT function.
(3) Suppressed energy production: Reduced mitochondrial ATP production efficiency, possibly due to intra-astrocytic or neuronal acidification.

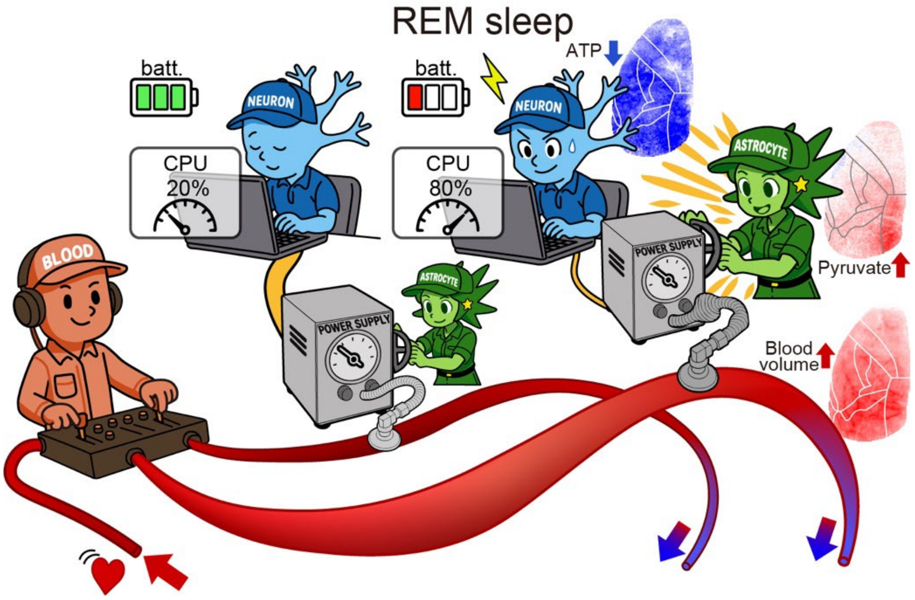

Supplementary Fig. 10: Schematic of dual-layered adaptive energy resource allocation strategy. Information processing in the brain demands energy, and limited resources must be efficiently allocated across functional submodules. While neuronal activity primarily drives energy delivery, blood circulation and metabolic processes also constrain computational capacity, forming a super-network that links information processing with metabolism. Our results indicate a homeostatic mechanism that continuously adjusts energy supply to neuronal demand. The brain dynamically regulates energy delivery by modulating blood flow, a principle exploited by functional MRI (fMRI), which infers computation from hemodynamic signals. However, our data reveal an additional astrocyte-mediated layer of regulation. During REM sleep, blood volume and astrocytic pyruvate increased, yet neuronal ATP decreased, indicating that neuronal energy availability cannot be fully captured by blood volume measures alone. We propose a dual-layered adaptive strategy: (1) vascular regulation of blood volume and (2) astrocytic control of substrate allocation. These mechanisms enable the brain to sustain complex computations with exceptional energy efficiency.

Description of Supplementary Movies

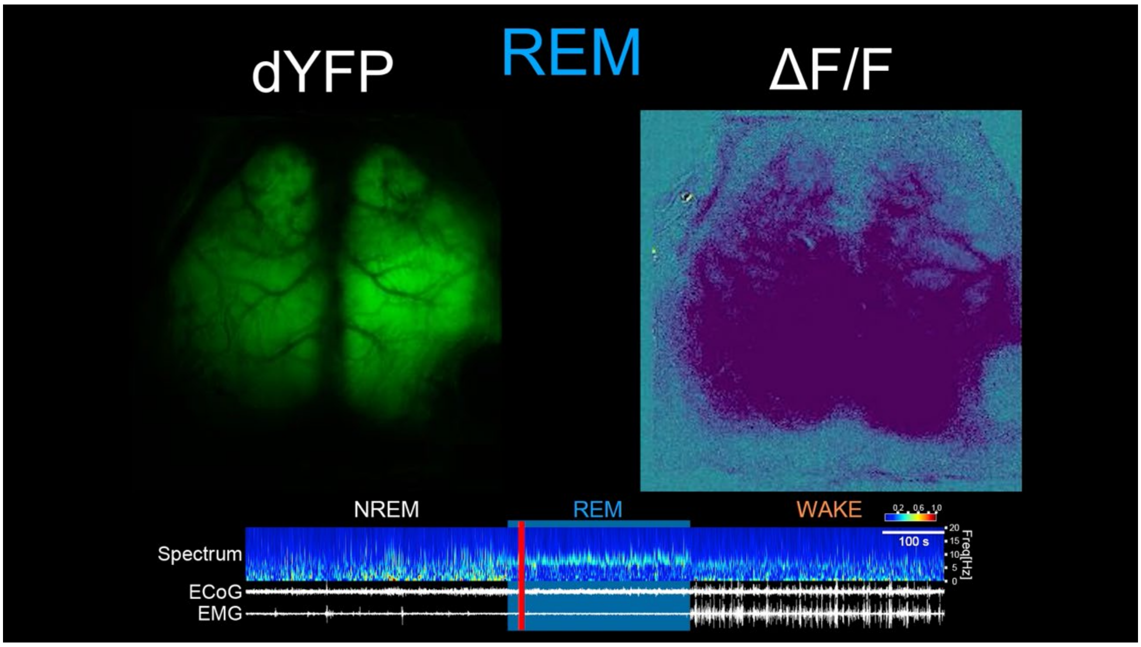

Supplementary Movie 1: Shadow imaging of brain blood volume (BBV) dynamics. Whole cortical view of the mouse brain acquired with a fluorescence stereo microscope through the intact skull. Excitation and emitted fluorescence from brain parenchymal cells are absorbed by hemoglobin, rendering blood vessels as dark shadows (left, dYFP). Thus, vessel dilation and constriction correspond to decreases and increases, respectively, in detected fluorescence. Fluctuations in dYFP intensity therefore primarily reflect local BBV dynamics. To enhance visibility, fluorescence images were normalized by subtracting and dividing by the basal fluorescence level (right, ΔF/F). Rapid BBV fluctuations were evident during NREM sleep, whereas a pronounced fluorescence decrease occurred upon REM onset, reflecting large vessel dilation and increased absorption. Upon awakening, fluorescence levels rapidly recovered.

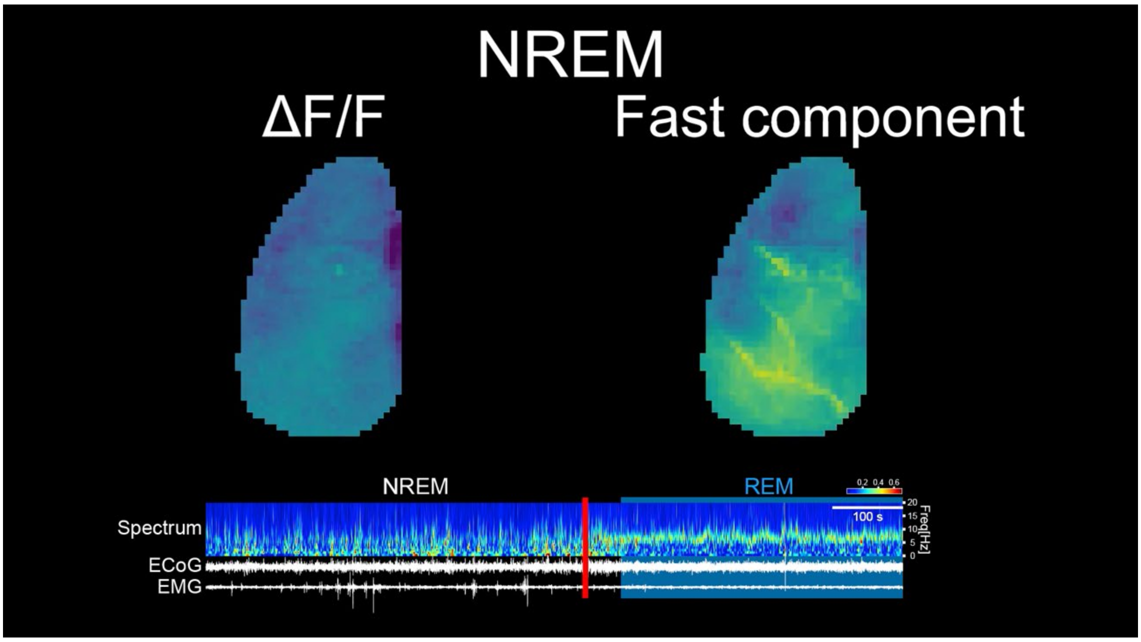

Supplementary Movie 2: Properties of the fast component of brain blood volume (BBV) fluctuations. Movie from the left hemisphere of a mouse brain. Left: ΔF/F of dYFP. Right: The fast component of BBV fluctuations was extracted by temporal band-pass filtering (0.05–0.2 Hz), revealing robust fast dynamics. The amplitude of these fluctuations decreased during REM sleep. Clustering analysis of correlated BBV activity demonstrated larger, fewer clusters during NREM compared to REM. Moreover, during NREM, the direction of correlated flow was consistently anterior-to-posterior with a propagation time of ~1 s, whereas this consistency was lost during REM.

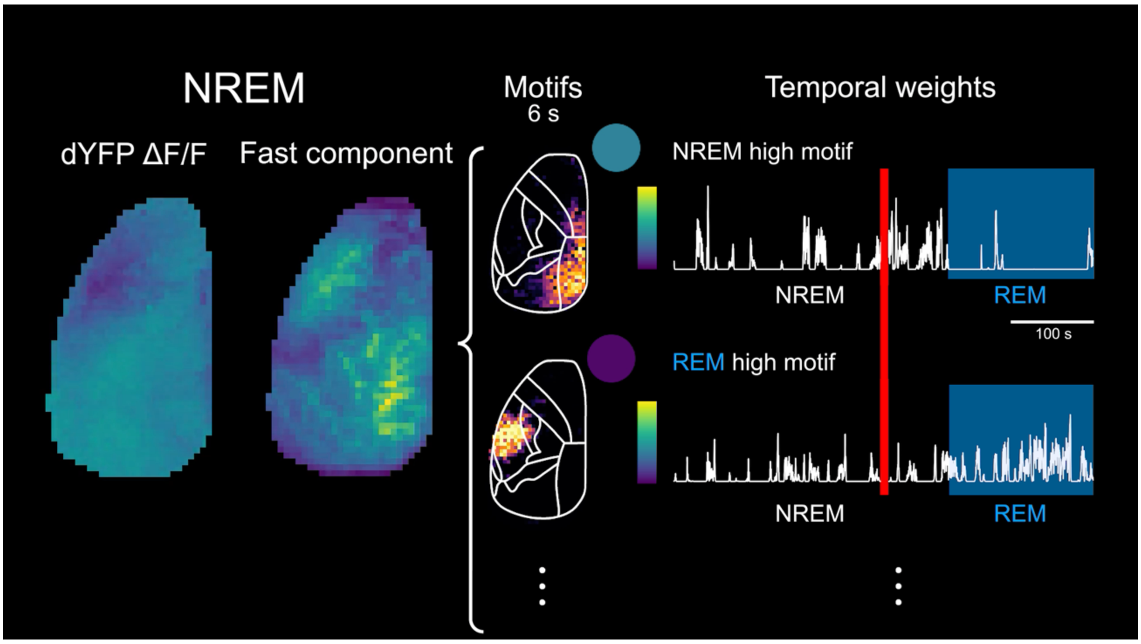

Supplementary Movie 3: Extraction of spatiotemporal motifs of brain blood volume (BBV) fluctuations. Movie from the left hemisphere of a mouse brain. Left: ΔF/F of dYFP. Right: The fast component of BBV fluctuations was extracted by temporal band-pass filtering (0.05–0.2 Hz), calculating the envelope of fluorescence intensity fluctuations, and frame-wise normalizing the intensity. This procedure revealed fast BBV dynamics that persisted during REM sleep, even when large vessel dilations dominated the original ΔF/F signal and caused slow fluorescence decreases. Spatiotemporal motifs of BBV fluctuations were identified using SeqNMF. Middle panels: Two representative motifs (6 s segments shown in loop). Far right: Temporal weights of these motifs. The upper motif ("NREM high") occurred predominantly during NREM and increased prior to REM onset, whereas the lower motif ("REM high") occurred more frequently during REM. Circles at the right shoulder of each motif indicate the motif's occurrence rate in the movie.

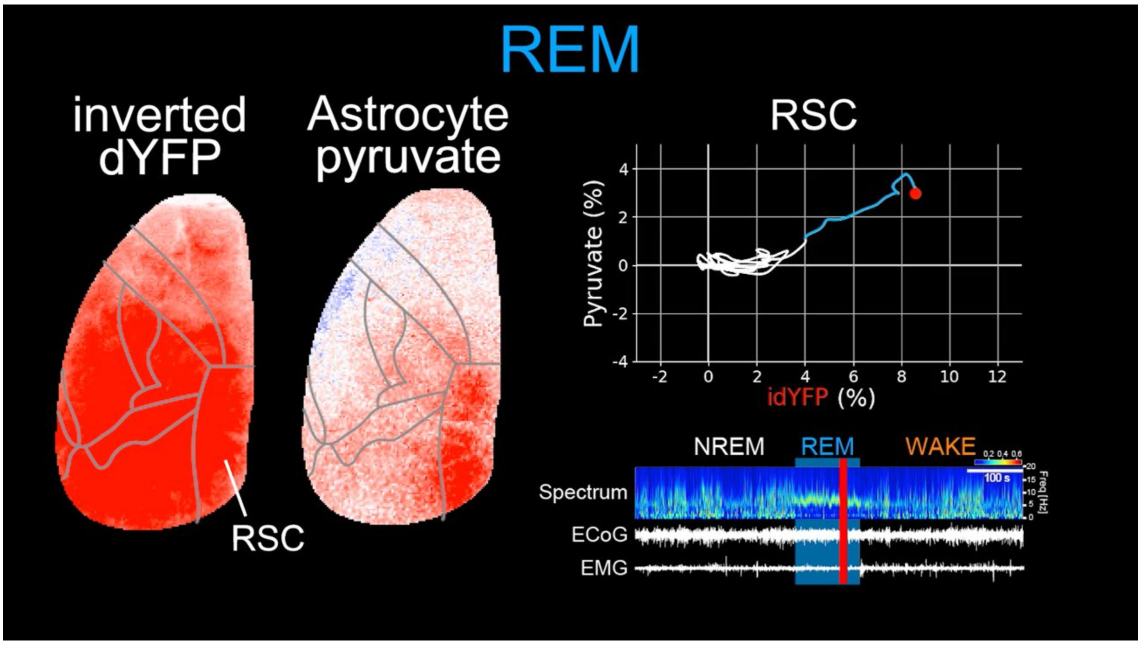

Supplementary Movie 4: Concurrent increases in brain blood volume (BBV) and astrocytic pyruvate during REM sleep. Movie from the left hemisphere of a transgenic mouse expressing a FRET-based fluorescent sensor for astrocytic cytosolic pyruvate (PYRS). Left: ΔF/F of idYFP (inverse direct excitation of YFP), which is insensitive to pyruvate but primarily reflects BBV dynamics. Right: Astrocytic pyruvate signals calculated as the difference between dYFP and fYFP (YFP emission excited by CFP; inversely sensitive to pyruvate concentration and to BBV). Upon entry into REM sleep, idYFP signals increased globally, indicating a widespread rise in BBV across the cortex. In parallel, pyruvate levels also increased, consistent with enhanced substrate supply associated with REM-related vasodilation.

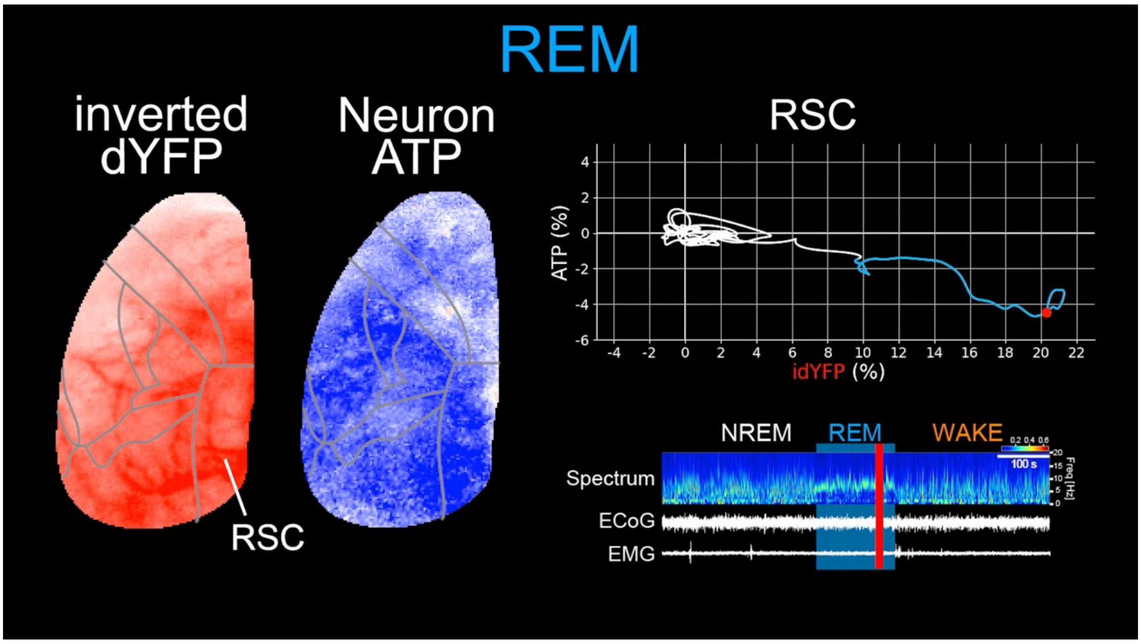

Supplementary Movie 5: Decrease in neuronal ATP during REM sleep. Movie from the left hemisphere of a transgenic mouse expressing a FRET-based fluorescent sensor for neuronal cytosolic ATP (ATeam). Left: ΔF/F of idYFP (inverse direct excitation of YFP), which is insensitive to ATP but primarily reflects BBV dynamics. Right: Neuronal ATP signals, calculated as the difference between fYFP (YFP emission excited by CFP; sensitive to ATP concentration but inversely affected by BBV) and dYFP. Upon entry into REM sleep, idYFP signals increased globally, indicating a widespread rise in BBV across the cortex. Unexpectedly, neuronal ATP levels decreased, despite the enhanced substrate supply expected with REM-associated vasodilation. This suggests the presence of an additional regulatory layer of energy management that adapts to the unique computational demands of REM sleep.
