# Supplementary material for: Energy paradox in REM sleep: balancing supply and consumption in brain metabolism
Source: Commun Biol. 2026 Jul 27;9:979. doi: 10.1038/s42003-026-10646-6 (PMC13408869; doi:10.1038/s42003-026-10646-6)
Supplement: Supplementary file 8 — Description of Additional Supplementary files [file 42003_2026_10646_MOESM8_ESM.pdf]

Description of Additional Supplementary files

Supplementary Data : The numerical source data underlying all graphs presented in the main and supplementary figures are provided with this paper as a Supplementary Data file.

Supplementary Movie 1: Shadow imaging of brain blood volume (BBV) dynamics. Whole cortical view of the mouse brain acquired with a fluorescence stereo microscope through the intact skull. Excitation and emitted fluorescence from brain parenchymal cells are absorbed by hemoglobin, rendering blood vessels as dark shadows (left, dYFP). Thus, vessel dilation and constriction correspond to decreases and increases, respectively, in detected fluorescence. Fluctuations in dYFP intensity therefore primarily reflect local BBV dynamics. To enhance visibility, fluorescence images were normalized by subtracting and dividing by the basal fluorescence level (right, ΔF/F). Rapid BBV fluctuations were evident during NREM sleep, whereas a pronounced fluorescence decrease occurred upon REM onset, reflecting large vessel dilation and increased absorption. Upon awakening, fluorescence levels rapidly recovered.

Supplementary Movie 2: Properties of the fast component of brain blood volume (BBV) fluctuations. Movie from the left hemisphere of a mouse brain. Left: ΔF/F of dYFP. Right: The fast component of BBV fluctuations was extracted by temporal band-pass filtering (0.05–0.2 Hz), revealing robust fast dynamics. The amplitude of these fluctuations decreased during REM sleep. Clustering analysis of correlated BBV activity demonstrated larger, fewer clusters during NREM compared to REM. Moreover, during NREM, the direction of correlated flow was consistently anterior-to-posterior with a propagation time of ~1 s, whereas this consistency was lost during REM.

Supplementary Movie 3: Extraction of spatiotemporal motifs of brain blood volume (BBV) fluctuations. Movie from the left hemisphere of a mouse brain. Left: ΔF/F of dYFP. Right: The fast component of BBV fluctuations was extracted by temporal band-pass filtering (0.05–0.2 Hz), calculating the envelope of fluorescence intensity fluctuations, and frame-wise normalizing the intensity. This procedure revealed fast BBV dynamics that persisted during REM sleep, even when large vessel dilations dominated the original ΔF/F signal and caused slow fluorescence decreases. Spatiotemporal motifs of BBV fluctuations were identified using SeqNMF. Middle panels: Two representative motifs (6 s segments shown in loop). Far

right: Temporal weights of these motifs. The upper motif (“NREM high”) occurred predominantly during NREM and increased prior to REM onset, whereas the lower motif (“REM high”) occurred more frequently during REM. Circles at the right shoulder of each motif indicate the motif’s occurrence rate in the movie.

Supplementary Movie 4: Concurrent increases in brain blood volume (BBV) and astrocytic pyruvate during REM sleep. Movie from the left hemisphere of a transgenic mouse expressing a FRET-based fluorescent sensor for astrocytic cytosolic pyruvate (PYRS). Left: ΔF/F of idYFP (inverse direct excitation of YFP), which is insensitive to pyruvate but primarily reflects BBV dynamics. Right: Astrocytic pyruvate signals calculated as the difference between dYFP and fYFP (YFP emission excited by CFP; inversely sensitive to pyruvate concentration and to BBV). Upon entry into REM sleep, idYFP signals increased globally, indicating a widespread rise in BBV across the cortex. In parallel, pyruvate levels also increased, consistent with enhanced substrate supply associated with REM-related vasodilation.

Supplementary Movie 5: Decrease in neuronal ATP during REM sleep. Movie from the left hemisphere of a transgenic mouse expressing a FRET-based fluorescent sensor for neuronal cytosolic ATP (ATeam). Left: ΔF/F of idYFP (inverse direct excitation of YFP), which is insensitive to ATP but primarily reflects BBV dynamics. Right: Neuronal ATP signals, calculated as the difference between fYFP (YFP emission excited by CFP; sensitive to ATP concentration but inversely affected by BBV) and dYFP. Upon entry into REM sleep, idYFP signals increased globally, indicating a widespread rise in BBV across the cortex. Unexpectedly, neuronal ATP levels decreased, despite the enhanced substrate supply expected with REM-associated vasodilation. This suggests the presence of an additional regulatory layer of energy management that adapts to the unique computational demands of REM sleep.
