# Supplementary material for: Energy paradox in REM sleep: balancing supply and consumption in brain metabolism
Source: Commun Biol. 2026 Jul 27;9:979. doi: 10.1038/s42003-026-10646-6 (PMC13408869; doi:10.1038/s42003-026-10646-6)
Supplement: Supplementary file 9 — Reporting Summary [file 42003_2026_10646_MOESM9_ESM.pdf]

nature portfolio

Corresponding author(s): Ko Matsui

Last updated by author(s): Jun 25, 2026

Reporting Summary

Nature Portfolio wishes to improve the reproducibility of the work that we publish. This form provides structure for consistency and transparency in reporting. For further information on Nature Portfolio policies, see our Editorial Policies and the Editorial Policy Checklist.

Statistics

For all statistical analyses, confirm that the following items are present in the figure legend, table legend, main text, or Methods section.

n/a	Confirmed	
☐	☒	The exact sample size (*n*) for each experimental group/condition, given as a discrete number and unit of measurement
☐	☒	A statement on whether measurements were taken from distinct samples or whether the same sample was measured repeatedly
☐	☒	The statistical test(s) used AND whether they are one- or two-sided<br>*Only common tests should be described solely by name; describe more complex techniques in the Methods section.*
☒	☐	A description of all covariates tested
☐	☒	A description of any assumptions or corrections, such as tests of normality and adjustment for multiple comparisons
☒	☐	A full description of the statistical parameters including central tendency (e.g. means) or other basic estimates (e.g. regression coefficient) AND variation (e.g. standard deviation) or associated estimates of uncertainty (e.g. confidence intervals)
☐	☒	For null hypothesis testing, the test statistic (e.g. *F*, *t*, *r*) with confidence intervals, effect sizes, degrees of freedom and *P* value noted<br>*Give P values as exact values whenever suitable.*
☒	☐	For Bayesian analysis, information on the choice of priors and Markov chain Monte Carlo settings
☒	☐	For hierarchical and complex designs, identification of the appropriate level for tests and full reporting of outcomes
☐	☒	Estimates of effect sizes (e.g. Cohen's *d*, Pearson's *r*), indicating how they were calculated

Our web collection on statistics for biologists contains articles on many of the points above.

Software and code

Policy information about availability of computer code

Data collection	In vivo imaging datasets using macro zoom microscope were collected using HCImage Live software.<br>ECoG and EMG signals were collected using Spike2 software.
Data analysis	All datasets were analyzed using custom code written in Python3.10.14.<br>All statistical analyses were performed using custom code written in Python3.10.14.

For manuscripts utilizing custom algorithms or software that are central to the research but not yet described in published literature, software must be made available to editors and reviewers. We strongly encourage code deposition in a community repository (e.g. GitHub). See the Nature Portfolio guidelines for submitting code & software for further information.

Data

Policy information about availability of data

All manuscripts must include a data availability statement. This statement should provide the following information, where applicable:

- Accession codes, unique identifiers, or web links for publicly available datasets
- A description of any restrictions on data availability
- For clinical datasets or third party data, please ensure that the statement adheres to our policy

The data that support the findings of the study are available from the corresponding author upon reasonable request.

Research involving human participants, their data, or biological material

Policy information about studies with human participants or human data. See also policy information about sex, gender (identity/presentation), and sexual orientation and race, ethnicity and racism.

Reporting on sex and gender	N/A
Reporting on race, ethnicity, or other socially relevant groupings	N/A
Population characteristics	N/A
Recruitment	N/A
Ethics oversight	N/A

Note that full information on the approval of the study protocol must also be provided in the manuscript.

Field-specific reporting

Please select the one below that is the best fit for your research. If you are not sure, read the appropriate sections before making your selection.

☒ Life sciences ☐ Behavioural & social sciences ☐ Ecological, evolutionary & environmental sciences

For a reference copy of the document with all sections, see nature.com/documents/nr-reporting-summary-flat.pdf

Life sciences study design

All studies must disclose on these points even when the disclosure is negative.

Sample size	Sample sizes were determined based on previous studies using similar imaging and behavioral paradigms. No statistical methods were used to predetermine sample size. For REM sleep imaging experiments, Thy1-ATeam transgenic mice (n = 6) and Mlc1-tTA::tetO-PYRS transgenic mice (n = 3) were used. For SNP injection experiments, Thy1-ATeam transgenic mice (n = 3) and Mlc1-tTA::tetO-PYRS transgenic mice (n = 3) were used. In all analyses, n refers to the number of individual animals.
Data exclusions	REM sleep episodes lasting less than 50 seconds were excluded from the analysis.
Replication	The main findings were reproduced across multiple independent animals, yielding comparable results.
Randomization	Animals were randomly assigned to experimental groups where applicable.
Blinding	No blinding was done in this study because knowledge of experimental conditions was required during data collection.

Reporting for specific materials, systems and methods

We require information from authors about some types of materials, experimental systems and methods used in many studies. Here, indicate whether each material, system or method listed is relevant to your study. If you are not sure if a list item applies to your research, read the appropriate section before selecting a response.

Materials & experimental systems

n/a	Involved in the study	
☒	☐	Antibodies
☒	☐	Eukaryotic cell lines
☒	☐	Palaeontology and archaeology
☐	☒	Animals and other organisms
☒	☐	Clinical data
☒	☐	Dual use research of concern
☒	☐	Plants

Methods

n/a	Involved in the study	
☒	☐	ChIP-seq
☒	☐	Flow cytometry
☒	☐	MRI-based neuroimaging

Animals and other research organisms

Policy information about studies involving animals; ARRIVE guidelines recommended for reporting animal research, and Sex and Gender in Research

Laboratory animals	Laboratory mice were used in this study. Experiments were performed using male transgenic mouse lines expressing fluorescent metabolite sensors (Thy1-ATeam and Mlc1-tTA::tetO-PYRS). Animals were housed under standard laboratory conditions with ad libitum access to food and water.
Wild animals	This study did not involve wild animals.
Reporting on sex	All experiments were conducted using male mice. Sex was not considered as a biological variable in this study.
Field-collected samples	This study did not involve samples collected from the field.
Ethics oversight	All animal procedures involved in this study were approved in advance and conducted in accordance with the Regulations for Animal Experiments and Related Activities at Tohoku University (2019LsA-017).

Note that full information on the approval of the study protocol must also be provided in the manuscript.

Plants

Seed stocks	N/A
Novel plant genotypes	N/A
Authentication	N/A
