# Supplementary material for: Energy paradox in REM sleep: balancing supply and consumption in brain metabolism
Source: Commun Biol. 2026 Jul 27;9:979. doi: 10.1038/s42003-026-10646-6 (PMC13408869; doi:10.1038/s42003-026-10646-6)
Supplement: Supplementary file 10 — Transparent Peer Review file [file 42003_2026_10646_MOESM10_ESM.pdf]

nature portfolio

Peer Review File

Energy paradox in REM sleep: balancing supply and consumption in brain metabolism

Corresponding Author: Professor Ko Matsui

This file contains all reviewer reports in order by version, followed by all author rebuttals in order by version.

Version 0:

Reviewer comments:

Reviewer #1

(Remarks to the Author)
The manuscript by Takahashi et al. investigates the coupling between cerebral blood flow and metabolic supply across different vigilance states using wide-field fluorescence imaging in live mice. The authors report a striking "energy paradox" during REM sleep: despite a global surge in brain blood volume (BBV) and astrocytic pyruvate levels, neuronal ATP levels decline sharply. The novelty of this study lies in the use of a non-invasive surgical approach combined with multi-parameter simultaneous imaging to analyze, in real time, the temporal sequence of blood flow changes across various brain regions during sleep. It also reveals the dynamics of energy metabolism between neurons and astrocytes under different brain states. However, several issues remain unaddressed:
1. The conclusion that the fYFP-dYFP linear relationship established during NREM holds true during the much larger hemodynamic surges of REM sleep is critical. At high blood volumes, hemoglobin absorption may become non-linear. To validate that the REM-associated deviation is a genuine metabolic signal rather than a scaling artifact, the authors should provide residual analysis plots (observed fYFP minus scaled dYFP vs. BBV amplitude) for representative ROIs. A synchronized shift during REM, independent of BBV magnitude, would substantially strengthen their claims.
2. The authors use dYFP as a combined proxy for both pH and BBV fluctuations without independent validation of their synchronization. Given wavelength-dependent differences in hemoglobin absorption and potential non-linear YFP quenching by pH, the subtraction method could inadvertently generate artifactual ATP "drops." A control experiment using an analyte-insensitive but pH/environment-sensitive fluorescent protein (e.g., a "dead" sensor or standalone Venus/EYFP) during REM transitions is necessary. If the ATeam signal drop significantly exceeds fluctuations of this control protein, it would provide critical evidence that the "Energy Paradox" has a metabolic origin.
3. The interpretation of the "Energy Paradox" is highly sensitive to whether the heatmaps represent absolute ΔF/F values or are normalized to the peak response within each map. The authors must clarify this and provide a quantified bar graph comparing the absolute ΔF/F of idYFP, pyruvate, and ATP specifically for SSb versus RSC. This would confirm whether the paradox persists in regions where the vascular supply surge is physically less prominent.
4. The choice of a 6-second window for seqNMF involves a trade-off between capturing the full hemodynamic profile and maintaining sufficient temporal resolution to distinguish discrete events. To enhance methodological clarity, the authors should briefly explain the rationale for selecting 6 seconds. Specifically, did they test other window lengths (e.g., 4 s or 10 s), and was 6 s identified as the optimal balance for capturing core motif structure without introducing excessive noise or pattern overlap?
5. Lack of regional comparison: For instance, in the SSb region, where blood flow remains relatively high in both NREM and REM sleep, what is the status of energy substrates in astrocytes and neurons under these conditions?
6. Unclear regional specificity of the "energy paradox": In which specific brain regions does the phenomenon of increased astrocytic pyruvate accompanied by decreased neuronal ATP occur? Does this occur uniformly across all cortical regions? This has not been clarified.
Minor points
1. Overall, the manuscript suffers from poor readability due to extensive computational analyses that are difficult to follow, particularly in Figure 4. It is recommended that the authors simplify, if possible, the presentation or provide more detailed descriptions to make the content more accessible to a broader audience.
2. It is also not very clear what is FIR-BRF in figure 1?
3. In Figure 2 and Figures 4d-e, how are the thresholds defined?
4. On Page 9, line 219, "figure 2c" should likely be "figure 2d".
5. In page 9, regarding the slow component analysis, it would be helpful to first introduce the 0.05-0.2 Hz band (fast

component), as its physiological relevance is explained. Otherwise, a reader might question why <0.05 Hz is considered "slow" and not, for example, 0.01 Hz or 0.02 Hz.
6. In Figure 3f, is the value shown also ΔF/F normalized to NREM?
7. The conclusion that the fast component in the SSb is "relatively preserved" during REM sleep is not convincing. The graph clearly shows a drop in BBV fast-component amplitude in the SSb during REM; it is simply less pronounced than in other regions.
8. In figure 4, it is not clear how the authors analyzed the time delay (TD). Given the highly fluctuating signals, how was it determined which signal leads the other?
9. In Figures 4b and 4c, what is represented on the y-axis? The methodology for this analysis is not clearly explained.

Reviewer #2

(Remarks to the Author)
I co-reviewed this manuscript with one of the reviewers who provided the listed reports. This is part of the Communications Biology initiative to facilitate training in peer review and to provide appropriate recognition for Early Career Researchers who co-review manuscripts.

Reviewer #3

(Remarks to the Author)
This study uses intact-skull widefield imaging to simultaneously monitor brain blood volume (BBV), astrocytic pyruvate, and neuronal ATP, aiming to reveal energy allocation during sleep-state transitions. The attempt to integrate neurovascular coupling, astrocytic metabolism, and neuronal energy status into a unified framework is valuable. The observation during REM sleep—BBV increase and astrocytic pyruvate elevation accompanied by neuronal ATP reduction—is intriguing and potentially important. However, several issues remain unresolved.
Previous studies have already shown that REM sleep is associated with vasodilation, increased glucose utilization in certain brain regions, elevated extracellular lactate, and enhanced astrocytic metabolism. Therefore, the findings of vascular dilation and increased astrocytic pyruvate during REM are not highly novel. In contrast, the observation that theta activity precedes and strongly correlates with vascular dilation shows some originality. The finding that astrocytic energy metabolism increases while neuronal ATP decreases is also notable, but the manuscript does not sufficiently explain the underlying mechanisms or biological significance.
Fig. 1: The theta–BBV coupling is interesting, but the theoretical explanation and causal interpretation remain insufficient. The authors report that theta-band ECoG power during NREM predicts BBV changes by ~4–5 seconds. However, three issues remain:
1. It is unclear why theta, rather than the more typical NREM rhythms such as delta or slow oscillations, best predicts BBV changes. Stronger theoretical justification is needed.
2. The temporal correlation between theta and BBV does not establish causality; the data only show that neural activity precedes vascular changes.
3. The mechanism underlying the relationship between theta activity and vascular changes is not adequately explained. In particular, Fig. 7 shows that theta and BBV changes appear before alterations in pyruvate or ATP during the pre-REM phase, suggesting that theta–BBV coupling and metabolic signals are not directly or immediately linked.
Fig. 2: The finding that dYFP signals show stronger spatial correlation and fewer clusters during REM is valid. However, the analysis concerns the spatial correlation structure of BBV-related signals rather than cortical state transitions or neuronal synchrony per se. Therefore, the authors should avoid overstating the results as indicating “greater cortical functional unity” or “stronger cortical synchrony” during REM. The data only support increased spatial synchronization of BBV fluctuations.
Fig. 3: The rationale for dividing signals into slow (<0.05 Hz) and fast (0.05–0.2 Hz) components is not sufficiently justified. While the separation is conceptually helpful for distinguishing global slow BBV increases from faster local fluctuations during REM, the choice of the 0.05 Hz cutoff and the 0.05–0.2 Hz fast band requires further explanation.
Transition from Fig. 3 to Fig. 4: The logical progression is unclear. Fig. 3 shows a reduction in the fast component amplitude during REM, but the manuscript then directly proceeds to lag-aware spatial correlation analysis without clearly explaining why amplitude analysis alone is insufficient or what specific biological question the time-delay analysis addresses.
Fig. 6: The SNP experiment shows that pharmacologically induced vasodilation increases BBV, astrocytic pyruvate, and neuronal ATP, with pyruvate rising faster than ATP. This supports the idea that increased BBV can be accompanied by changes in energy-related signals. However, the authors’ interpretation is too strong. The data do not demonstrate a causal chain (BBV increase → astrocytic pyruvate increase → neuronal ATP increase), particularly because pyruvate and ATP measurements were obtained from different groups of mice and SNP may have effects beyond simply increasing BBV.
Fig. 7: This figure suggests that vascular changes do not immediately induce changes in pyruvate or ATP. During the pre-REM phase, both theta power and BBV increase before REM onset, but astrocytic pyruvate and neuronal ATP do not change correspondingly. This indicates a temporal dissociation between neurovascular changes and metabolic signals. Given that SNP-induced BBV increases (Fig. 6) are associated with metabolic changes, the authors should discuss why vascular changes during natural sleep transitions produce different metabolic consequences compared with pharmacological vasodilation.
Fig. 8: The core observation—BBV increase and astrocytic pyruvate elevation accompanied by neuronal ATP reduction during REM, especially in posterior cortex (RSC/V1)—is novel and important. However, the mechanistic interpretation appears premature.
1. The explanation of astrocytic pyruvate as a direct reflection of glucose supply is overly simplistic. Pyruvate levels can also be influenced by glycogen mobilization, lactate/pyruvate transport, and mitochondrial consumption.
2. Although the “BBV↑ / pyruvate↑ / ATP↓” pattern is robust, the proposed mechanisms (increased ATP consumption, substrate transport limitations, altered mitochondrial metabolism) remain speculative and require further experimental

support.

Reviewer #4

(Remarks to the Author)
This is a nice study about the relation between brain state (NREM/REM sleep and wakefulness), hyperemia, and metabolite levels. The experimental procedures appears to be well-conducted, although not necessarily well-designed for the goal of the study (see below). However, I have some concerns about both authors' assumptions on measured quantities and, accordingly, interpretation of the results. These are described below. I have found the paper interesting, but quite unbalanced. Specifically, there is a lot of space and details devoted to the analysis of the spatio-temporal cortical fluorescence patterns, and then a too simplistic treatment of the metabolite data, which is forced to biochemical interpretations that are not entirely supported by the measurements. Overall, the finding that activity-CBF in sleep are characterized by an anterior-posterior axis is not entirely new (Wang et al., 2022; Stephan et al., 2025). Similarly, the rise in pyruvate during REM sleep is expected as it has been repeatedly observed that lactate drops during NREM sleep compared to either REM or wakefulness (Naylor et al., 2012; Clegern et al., 2012; Shram et al., 2002; Rempe et al., 2014; Dash et al., 2012; Cespuglio et al., 2013). The ATP finding is more interesting, although expected on the basis of previous studies (Dworak et al., 2010). The main message of the paper, i.e., assigning metabolite dynamics to either neurons or astrocytes, may suffer of sub-optimal study design (see below). This is unfortunate, because the study has clearly been effortful, and the quality of data (figures, videos) is high. My suggestion would be to remove the metabolic interepretations and slightly reframe the presentation of the study goal/findings.

Introduction

Page 3: While introducing ionic homeostasis and Na/K-ATPase (NKA) activity, please mention that not only neuronal NKA is stimulated during neuronal activity, but also astrocytic NKA. Astrocytes are though to account for at least 1/3 of extracellular K+ reuptake, and correspondingly 20-25% of cortical energy expenditure.

Pages 3-4 lines 67-81: While it is true that pyramidal neurons (as opposed to interneurons) do not directly contact the vasculature, this does not mean that neurons rely on astrocyte-derived lactate (e.g., astrocyte-neuron lactate shuttle). This has been quite debated in the field, so I'm not citing the whole literature. Briefly, Nehlig et al. (2003) demonstrated that neurons take up >50% of blood-borne glucose. Simpson et al. (2007) substantiated such observation by mathematical modeling of GLUT transporter proteins. To name just a few follow-up studies, confirmations of direct neuronal glucose uptake can be found in Patel et al. (2014), Lundgaard et al. (2015), and more recently in Li et al. (2023). Overall, as discussed repeatedly by Leif Hertz, Gerald Dienel, and Douglas Rothman (to name some of the most prominent scientists in the field) both neurons and astrocytes can take up glucose and both can release lactate under specific circumstances, possibly depending on brain state. Thus, the argument that astrocytic pyruvate utilization reduces availability of lactate for neurons is rather weak. Besides, astrocytes mobilize glycogen during high activity (in a K+ uptake-related manner), which can be another source of pyruvate for either oxidation or export. It has been further demonstrated by Gandhi et al. (2009), Ball et al. (2010), and Lundgaard et al. (2017) that intracerebrally produced lactate predominantly leaves the brain via pervivascular/lymphatic routes. These clearance pathways are thought to be dependent on the sleep-wake cycle, further complicating the picture.

Page 4 lines 84-94 : Cellular ATP levels are only minimally influenced by energy demand because ATP is buffered by creatine kinase and adenylate kinase (among others), and rapidly replenished by glycolysis and oxidative phosphorylation. As the authors states, it is very difficult to measure changes in ATP.

Page 4 lines 109-110: Again, the observation that pyruvate increases following BBV surge cannot be ascribed to astrocytes only.

Page 4 lines 114-118: The authors are right in xcluding options (1) and (2). REM sleep is as expensive as active waking, i.e., more expensive than quiet waking. Thus, it is expected that energy demand rises during the transition from NREM to REM sleep.

Results

Page 7 lines 169-172 and Page 13 lines 336-337: What support is given to exclude substantial pH effects? What is the range of pH affecting each fluorescence signal? This is relevant especially because the authors discuss the results suggesting an involvement of astrocytic acidification (Discussion, Page 19 lines 494-503)

Page 8 lines 199-201: How the finding of theta-band ECoG relates to literature (Hillman??)

Page 9 lines 214-216: Large-scale anterior-posterior waves are also present during anesthesia (e.g., ketamine-xylazine). It'd have been interesting to compare the patterns in NREM sleep and anesthesia. Did the authors have some observations during KX or other anesthetics?

Page 12 lines 323-324: This statement ("prepare the metabolic landscape for REM sleep") is too speculative. Please remove.

Page 12 line 326 (also Page 13 line 329, again Page 15 line 389, and possibly more occurrences): Please avoid using ambiguous terminology, as "energy molecule" is not appropriate, and highly misleading for pyruvate. Specifically, metabolite levels (pyruvate and ATP) cannot be used as proxy of metabolic rates. Indeed, a decrease might indicate reduced production, increased consumption, reduced uptake, increased clearance/washout, or a any combination thereof. Since "energy metabolism" is related to metabolic rates, it is not appropriate to state that this study assessed energy dynamics, also considering the complexities of intracellular ATP buffering system and the intercellular lactate trafficking mentioned above.

Page 13 lines 334-335: If I understand correctly, the pyruvate sensor was only expressed in astrocytes, and the ATP sensor was only expressed in neurons. If that's the case, the finding (described on Page 14 lines 361-366) that pyruvate increases and ATP decreases upon vasodilation cannot be interpreted in a cellular-centered manner. Very likely pyruavet is increasing in all tissue compartments (extracellular, neurons, astrocytes) and possibly the same for ATP (neuronal and astrocytic). Since pyruvate and ATP are estimated only in one cellular compartment, the authors' conclusion that "blood volume enhances the energy supply, first boosting astrocytic pyruvate levels, which is then followed by an increase in neuronal ATP production" remains speculative at best. I understand that this FRET experiments are hard to carry out, but a good study design would have attempted measuring the same metabolite in different cell types (of course in different animals, which is fine).

Page 14 lines 370-371: I'm not sure "preparatory" is appropriate here. Please notice that sleep stages/substages are not homogeneous in terms of neuromodulators NE and ACh. These neuromodulators significantly influence baseline CBF, with NE typically leading to vasoconstriction and Ach vasodilation. Transition periods between NREM and REM are associated with progressive reduction of NE and increase of ACh. Interestingly, neuromodulators (NE and ACh, but also 5-HT) are released in a synchronized fashion during NREM sleep. In other words, their release is coordinated spatio-temporally (Teng et al., 2025), which might well be related to the "wave"-like hyperemia observed in this and other studies.

Discussion

Page 17 lines 424-425: The first sentence states something that is already well known.

Page 18 lines 452-455: The possibility that astrocytes may drive state transitions is interesting and it has been proposed long ago. However, there is no result in the present study to support that claim.

Page 18 lines 466-468: Literature strongly indicates that the increase in pyruvate (and lactate) durind REM sleep does not reflect increased supply (as the authors suggest), but a rise in metabolic rate (mainly glycolsysis) in the transition from NREM to REM. This consideration makes the subsequent statement less "paradoxical" (in authors' words: "Paradoxically, despite this increased supply, neuronal cytosolic ATP levels markedly decreased"). That's because it is not increased supply but increased demand, as the authors themselves subsequently discuss, correctly suggesting other sources of energy expenditure in addition to NKA (e.g., plasticity). In addition to characterize the study being in the title ("The paradox in REM sleep"), the same wording ("paradoxical") appears on Page 20 line 522, which the authors relate to fMRI. I don't understand the argument, because increased CBF and CBV during stimulation coexist with increased energy demand and small but significant ATP reductions, all of which is physiological.

Statistical Analysis

I have not examined the statistical methods in full detail. Given the amount of concerns stated above, I will assess whether the statistical analysis is appropriate in a subsequent review round should the paper progress in the revision process.

Version 1:

Reviewer comments:

Reviewer #1

(Remarks to the Author)
The authors have addressed the majority of my concerns. Although it still takes time to understand the whole logic behind the calculation, the text reads much more reader-friendly.

Reviewer #2

(Remarks to the Author)
I co-reviewed this manuscript with one of the reviewers who provided the listed reports. This is part of the Communications Biology initiative to facilitate training in peer review and to provide appropriate recognition for Early Career Researchers who co-review manuscripts.

Reviewer #3

(Remarks to the Author)
I have no further comments.

Additionally, I carefully reviewed the authors' rebuttal and responses to Reviewer #4's concerns. Overall, I think the authors have addressed many of the major conceptual criticisms in a constructive and scientifically appropriate manner.

In particular, the authors substantially revised the framing of the metabolic findings and now more carefully distinguish between measured observations and mechanistic interpretation. Several points raised by Reviewer #4 were appropriately addressed, including:

clarification that pyruvate and ATP measurements reflect intracellular metabolite levels rather than direct measurements of metabolic flux or "energy dynamics";
removal or softening of overly causal or teleological language such as "preparatory" and "energy molecule";
clearer acknowledgment that pyruvate and ATP were measured in different cellular compartments and different animals, limiting cell-type-specific mechanistic conclusions;
broader discussion of alternative metabolic frameworks, including direct neuronal glucose uptake, astrocytic glycogen metabolism, and neuromodulatory influences;
improved discussion of pH/hemodynamic confounds and internal correction strategies;
reduction of strong astrocyte-centered interpretations and causal claims.
Importantly, the authors now present the study in a more balanced way, emphasizing that the work primarily identifies state-dependent associations among vascular dynamics and intracellular metabolite signals rather than establishing a definitive mechanistic pathway.

I also think the authors appropriately acknowledged the limitations of their experimental design, especially regarding the inability to infer causal or sequential relationships between astrocytic pyruvate and neuronal ATP dynamics.

That said, some conceptual limitations naturally remain unresolved. In particular, the study still cannot fully distinguish whether the observed metabolite changes primarily reflect altered supply, consumption, transport, buffering, or clearance processes. Similarly, the use of the term "paradox" may still be somewhat interpretive, although the revised manuscript now defines this more carefully as a dissociation between vascular/metabolite-related signals rather than a contradiction to known physiology.

Overall, however, I think the authors have responded to Reviewer #4's concerns in good faith and substantially improved the rigor and interpretational restraint of the manuscript. While some mechanistic questions remain open, I do not believe the remaining issues fundamentally preclude publication, provided the revised conservative framing is maintained throughout the manuscript.

Open Access This Peer Review File is licensed under a Creative Commons Attribution 4.0 International License, which permits use, sharing, adaptation, distribution and reproduction in any medium or format, as long as you give appropriate credit to the original author(s) and the source, provide a link to the Creative Commons license, and indicate if changes were made.
In cases where reviewers are anonymous, credit should be given to 'Anonymous Referee' and the source.

The images or other third party material in this Peer Review File are included in the article's Creative Commons license, unless indicated otherwise in a credit line to the material. If material is not included in the article's Creative Commons license and your intended use is not permitted by statutory regulation or exceeds the permitted use, you will need to obtain permission directly from the copyright holder.
To view a copy of this license, visit https://creativecommons.org/licenses/by/4.0/

Tohoku University, Graduate School of Life Sciences

Super-network Brain Physiology
2-1-1 Katahira, Aoba-ku, Sendai 980-8577 JAPAN
Phone: +81-22-217-6209, Fax: +81-22-217-6211
E-mail: matsui@ tohoku.ac.jp
http://www.ims.med.tohoku.ac.jp/matsui/

Manuscript ID: COMMSBIO-26-1065-T
Title: Energy paradox in REM sleep: differential strategies balancing metabolism and computation across brain states
Corresponding author: Ko Matsui

Point-to-point rebuttal to the referees' comments.

We are extremely grateful to the reviewers for their valuable feedback on our manuscript and appreciate their interest in our study. We have revised the manuscript with the aim of addressing the reviewers' concerns and suggestions to their satisfaction. Below, we have provided responses to each of the comments from both reviewers. The original comments are highlighted in blue, while our responses are provided in black.

Reviewer #1 (Remarks to the Author):

The manuscript by Takahashi et al. investigates the coupling between cerebral blood flow and metabolic supply across different vigilance states using wide-field fluorescence imaging in live mice. The authors report a striking "energy paradox" during REM sleep: despite a global surge in brain blood volume (BBV) and astrocytic pyruvate levels, neuronal ATP levels decline sharply. The novelty of this study lies in the use of a non-invasive surgical approach combined with multi-parameter simultaneous imaging to analyze, in real time, the temporal sequence of blood flow changes across various brain regions during sleep. It also reveals the dynamics of energy metabolism between neurons and astrocytes under different brain states. However, several issues remain unaddressed:

We thank the reviewer for the positive assessment of the novelty and significance of our

study. We also appreciate the reviewer's insightful comments, which have helped us improve the clarity and rigor of the manuscript.

1. The conclusion that the fYFP-dYFP linear relationship established during NREM holds true during the much larger hemodynamic surges of REM sleep is critical. At high blood volumes, hemoglobin absorption may become non-linear. To validate that the REM-associated deviation is a genuine metabolic signal rather than a scaling artifact, the authors should provide residual analysis plots (observed fYFP minus scaled dYFP vs. BBV amplitude) for representative ROIs. A synchronized shift during REM, independent of BBV magnitude, would substantially strengthen their claims.

We thank the reviewer for this insightful suggestion.

To address this concern, we have added a new supplementary figure (Supplementary Fig. 6) presenting fYFP and dYFP signals during SNP-induced vasodilation and during the NREM-to-REM transition, together with the corresponding fYFP–dYFP scatter trajectories and residual analysis.

Both SNP-induced vasodilation and REM sleep involve large increases in BBV. However, the direction of the residual (fYFP minus scaled dYFP) was opposite in these two conditions. If the REM-associated deviation were primarily caused by non-linear hemoglobin absorption at high blood volume, the residual should depend mainly on BBV amplitude and thus show a consistent direction across conditions. The observed bidirectional behavior is therefore not explained by a simple scaling artifact and instead supports the interpretation that the REM-associated deviation reflects a genuine metabolic signal.

We note that residual analysis alone cannot fully exclude hemodynamic contributions, as metabolite levels may covary with BBV. However, the opposite-direction responses observed under SNP and REM provide an independent constraint that is not accounted for by optical non-linearity alone.

Finally, we acknowledge that the fYFP–dYFP linear relationship was established within the

range of NREM hemodynamic fluctuations and is extrapolated to the larger BBV changes observed during REM. This limitation has been explicitly noted in the Methods (page 27, paragraph 3).

2. The authors use dYFP as a combined proxy for both pH and BBV fluctuations without independent validation of their synchronization. Given wavelength-dependent differences in hemoglobin absorption and potential non-linear YFP quenching by pH, the subtraction method could inadvertently generate artifactual ATP "drops." A control experiment using an analyte-insensitive but pH/environment-sensitive fluorescent protein (e.g., a "dead" sensor or standalone Venus/EYFP) during REM transitions is necessary. If the ATeam signal drop significantly exceeds fluctuations of this control protein, it would provide critical evidence that the "Energy Paradox" has a metabolic origin.

We thank the reviewer for this important comment highlighting the need to distinguish between hemodynamic, pH, and ATP-related signals.

We agree that an independent analyte-insensitive but pH/environment-sensitive fluorescent protein could serve as an external control. However, in the present study, we employed an internal reference based on dYFP, which originates from the same YFP fluorophore as fYFP and is largely insensitive to ATP, thereby capturing shared influences such as pH fluctuations and hemodynamic effects within the same optical and molecular context.

This approach relies on the assumption that pH similarly affects both fYFP and dYFP signals. In addition, any residual differences arising from wavelength-dependent hemoglobin absorption or optical scaling were further corrected by linear normalization, as detailed in Supplementary Fig. 6. These assumptions and procedures are now explicitly described in the manuscript (page 15, paragraph 3; page 16, paragraph 1) and Methods (page 27, paragraph 3).

Under this framework, if the observed decrease in the fYFP component were primarily driven by pH or hemodynamic effects, these influences would be expected to affect both fYFP and dYFP similarly and thus be largely removed by subtraction and normalization. However, we

consistently observe that the decrease in the fYFP component in the ATeam signal exceeds the fluctuations captured by dYFP during REM sleep, resulting in a residual signal that cannot be explained by shared pH or hemodynamic contributions alone.

Importantly, because this correction is performed within the same fluorophore and optical pathway, it inherently controls for factors that would not be fully matched by an external fluorescent protein. Therefore, the observed residual signal provides strong evidence that the ATP decrease reflects a component not accounted for by pH or BBV fluctuations.

While we acknowledge that this approach cannot fully exclude all possible nonlinear effects, the combination of internal referencing and normalization provides a stringent constraint against artifactual interpretations.

3. The interpretation of the "Energy Paradox" is highly sensitive to whether the heatmaps represent absolute ΔF/F values or are normalized to the peak response within each map. The authors must clarify this and provide a quantified bar graph comparing the absolute ΔF/F of idYFP, pyruvate, and ATP specifically for SSb versus RSC. This would confirm whether the paradox persists in regions where the vascular supply surge is physically less prominent.

Thank you for raising this important point.

We first clarify that all cortical heatmaps in Fig. 8 represent absolute ΔF/F values and are not peak-normalized. This has now been explicitly stated in the figure legends (Fig. 8 legend, page 51).

A quantitative comparison across regions is already provided in Fig. 8k, l, which show mean ± SEM of absolute ΔF/F values for idYFP, astrocytic pyruvate (k), and neuronal ATP (l) across multiple cortical regions during PreREM, REM, and WAKE.

Importantly, the key feature of the “energy paradox” is not the magnitude of the responses but their direction: during REM sleep, astrocytic pyruvate increases whereas neuronal ATP decreases. This opposite-direction relationship is consistently observed across cortical

regions, including those with relatively smaller vascular responses (e.g., SSb). Thus, the paradoxical pattern is preserved despite regional differences in vascular amplitude, supporting the interpretation that the energy paradox reflects a widespread metabolic phenomenon rather than being restricted to regions with the largest vascular changes.

To further improve clarity, we have added a new supplementary figure (Supplementary Fig. 7) showing the same data as bar graphs for direct comparison of absolute ΔF/F values across regions.

4. The choice of a 6-second window for seqNMF involves a trade-off between capturing the full hemodynamic profile and maintaining sufficient temporal resolution to distinguish discrete events. To enhance methodological clarity, the authors should briefly explain the rationale for selecting 6 seconds. Specifically, did they test other window lengths (e.g., 4 s or 10 s), and was 6 s identified as the optimal balance for capturing core motif structure without introducing excessive noise or pattern overlap?

We thank the reviewer for this important methodological question on motif extraction.

The motif duration parameter (6 seconds) was chosen based on both the frequency content of the input signals and the stability of the extracted motifs. The dYFP signal was band-pass filtered between 0.05 and 0.2 Hz prior to envelope extraction, and the power spectrum of this envelope signal showed that its frequency components were broadly distributed across periods longer than 6 seconds (< 0.16 Hz), suggesting that fast-component oscillations may contain spatiotemporal patterns at multiple time scales.

We therefore aimed to identify the longest motif duration that could be robustly extracted across recordings. When longer windows were tested (e.g., 10 s), the extracted motifs were not stable across recordings. Accordingly, a 6-second window was selected as the longest duration that allowed consistent identification of spatiotemporal motifs, representing a minimal repeating unit of the underlying dynamics. Shorter windows were not systematically explored, as our primary objective was to identify the longest stable motif duration, rather than to optimize temporal resolution across shorter time scales.

Although beyond the scope of the current study, a larger dataset may enable the extraction of longer and more complex motifs that capture higher-order spatiotemporal structure. This rationale has been added to the Supplementary Methods (pages 13, paragraph 4).

5. Lack of regional comparison: For instance, in the SSb region,where blood flow remains relatively high in both NREM and REM sleep, what is the status of energy substrates in astrocytes and neurons under these conditions?

We thank the reviewer for the comment on regional specificity.

As described in our response to Comment 3, Fig. 8k, l (and the newly added Supplementary Fig. 7) present the absolute ΔF/F values of idYFP, astrocytic pyruvate, and neuronal ATP across multiple cortical regions during PreREM, REM, and WAKE.

Importantly, even in the SSb region, where vascular changes are relatively less pronounced, REM sleep is consistently associated with an increase in astrocytic pyruvate and a decrease in neuronal ATP. Thus, the paradoxical pattern is preserved in SSb despite its distinct vascular profile.

Across all cortical regions examined, including SSb, astrocytic pyruvate increased and neuronal ATP decreased during REM sleep, indicating that the energy paradox is not restricted to regions with the largest vascular responses. We also observed an anterior-to-posterior gradient, with smaller changes in anterior regions and progressively larger changes toward posterior regions.

To make this regional specificity more explicit, we have added a corresponding description to the Results (page 18, paragraph 4).

6. Unclear regional specificity of the "energy paradox": In which specific brain regions does the phenomenon of increased astrocytic pyruvate accompanied by decreased neuronal ATP

occur? Does this occur uniformly across all cortical regions? This has not been clarified

We thank the reviewer for this question regarding the regional specificity of the energy paradox.

The paradoxical pattern, an increase in astrocytic pyruvate accompanied by a decrease in neuronal ATP during REM sleep, was consistently observed across all eight cortical regions examined (MOs, MOp, SSu, SSl, SSb, PPC, RSC, and V1), as shown in Fig. 8k, l and Supplementary Fig. 7.

Thus, the energy paradox is a region-wide feature of REM sleep rather than being confined to specific cortical areas. At the same time, the magnitude of the changes was not uniform, showing an anterior to posterior gradient, with smaller changes in anterior regions and progressively larger changes toward posterior regions, as now described in the Results (page 18, paragraph 4).

[Minor Points]

1. Overall, the manuscript suffers from poor readability due to extensive computational analyses that are difficult to follow, particularly in Figure 4. It is recommended that the authors simplify, if possible, the presentation or provide more detailed descriptions to make the content more accessible to a broader audience.

Thank you for this comment.

We acknowledge that the computational analyses, particularly those presented in Figure 4, may be complex. In the revised manuscript, we have clarified the descriptions and improved the logical flow of the analyses to enhance readability. We have also refined the corresponding explanations in the text to better guide the reader through the analytical steps.

2. It is also not very clear what is FIR-BRF in figure 1?

We thank the reviewer for pointing out that the description of FIR-BRF was not sufficiently clear. In the revised manuscript, we have added a brief explanation of the FIR-BRF in the Results section (page 9, paragraph 2), clarifying that it is a data-driven response kernel that models how past neuronal activity is transformed into hemodynamic signals, fitted individually to each recording, in contrast to the generic double-gamma HRF.

In addition, we have added a new section to the Supplementary Methods (from page 10, paragraph 3) describing the mathematical definition of the FIR-BRF and the procedure used to estimate it from each recording. We believe these additions make both the conceptual role and computation of the FIR-BRF clear to the reader.

3. In Figure 2 and Figures 4d-e, how are the thresholds defined?

Thank you for this important question. We would like to clarify how the clustering thresholds were defined.

For Fig. 2, the clustering distance threshold was set such that the dYFP signals during REM sleep were partitioned into five clusters. This choice provides a convenient reference point for comparing spatial synchronization across brain states, as REM sleep exhibits stronger large-scale synchronization than NREM. Importantly, the same threshold was then applied to the NREM data, resulting in a larger number of clusters, which quantitatively reflects the reduced spatial synchronization of BBV fluctuations in NREM relative to REM.

Conversely, for Fig. 4 (b-c and d-e), the threshold was defined based on the NREM data, where the fast component (0.05 to 0.2 Hz) shows stronger global synchronization than in REM. The threshold was set such that NREM data were partitioned into five clusters, and the identical threshold was then applied to REM data, yielding a larger number of clusters and thereby revealing the reduced long-range synchronization during REM.

Thus, in both analyses, the threshold was not tuned independently for each condition but instead defined using one reference state and then consistently applied to the other, enabling direct comparison of spatial synchronization across brain states.

These definitions are now explicitly described in the Results section (page 10, paragraph 2; page 12, paragraph 3).

4. On Page 9, line 219, "figure 2c" should likely be "figure 2d".

We thank the reviewer for catching this error. We have corrected “Figure 2c” to “Figure 2d” in the revised manuscript (page 10, paragraph 2).

5. In page 9, regarding the slow component analysis, it would be helpful to first introduce the 0.05-0.2 Hz band (fast component), as its physiological relevance is explained. Otherwise, a reader might question why <0.05 Hz is considered "slow" and not, for example, 0.01Hz or 0.02 Hz.

We thank the reviewer for this helpful suggestion. We agree that the distinction between slow and fast components should be introduced more clearly to avoid potential confusion.

In the revised manuscript, we have expanded the relevant passage in the Results (page 10, paragraph 3) to explicitly clarify the rationale for the frequency separation. Specifically, the slow component (< 0.05 Hz) captures the large-amplitude BBV increase associated with REM onset, which unfolds over tens of seconds, whereas the fast component (0.05–0.2 Hz) corresponds to the established frequency range of vasomotion.

Regarding the order of presentation, we chose to introduce the slow component first because it directly relates to the prominent REM-associated BBV increase described in Fig. 3, thereby providing a clear physiological context before analyzing the faster fluctuations in detail in Fig. 4. We believe this structure improves the overall readability while ensuring that the rationale for the frequency separation is clearly stated at the outset.

6. In Figure 3f, is the value shown also ΔF/F normalized to NREM?

We thank the reviewer for pointing this out. The values shown in Fig. 3f represent the amplitude of the fast component expressed as ΔF/F (%), and are not normalized to NREM. The REM/NREM panel shown on the right represents the ratio between the two conditions, whereas the NREM and REM maps themselves show absolute ΔF/F (%) values. The unit label (%) was missing from the color bar in the original figure, which may have caused confusion. We have added the unit to the color bar and clarified the caption accordingly (page 41, paragraph 1).

7. The conclusion that the fast component in the SSb is "relatively preserved" during REM sleep is not convincing. The graph clearly shows a drop in BBV fast-component amplitude in the SSb during REM; it is simply less pronounced than in other regions.

We thank the reviewer for this accurate observation. We agree that the term “relatively preserved” was imprecise. The fast dYFP component in SSb does decrease during REM sleep, as correctly pointed out by the reviewer, but the reduction is less pronounced compared to other cortical regions.

We have revised the relevant passages to explicitly describe this as a smaller relative decrease in SSb, and have adjusted the description of the REM/NREM amplitude ratio accordingly (page 11, paragraph 3) to better reflect this quantitative relationship.

8. In figure 4, it is not clear how the authors analyzed the time delay (TD). Given the highly fluctuating signals, how was it determined which signal leads the other?

We thank the reviewer for pointing out that the description of TD was insufficient. In the revised manuscript, we have clarified the definition of TD in two places.

First, when TD is initially introduced (page 12, paragraph 1), we now state explicitly that the lagged cross-correlation function was computed for each tile pair, and that TD was defined as the time lag at which the cross-correlation reached its peak.

Second, when the sliding window analysis is introduced, we have added that positive TD values indicate that the anterior signal leads the posterior signal, and that the sliding window approach tracks how this leading/lagging relationship shifts over time within a single episode (pages 13, paragraph 3).

Importantly, by computing TD within successive time windows, this approach enables robust estimation of leading/lagging relationships despite the fluctuating nature of the signals. We believe these additions make the TD computation and its interpretation transparent to the reader.

9. In Figures 4b and 4c, what is represented on the y-axis? The methodology for this analysis is not clearly explained.

We thank the reviewer for this question. In Figures 4b and 4c, each row (y-axis) represents the XC_{TD} values between a given cortical tile and all other tiles, and the rows are reordered by hierarchical clustering.

In the revised manuscript (page 12, paragraph 2), we have clarified how these matrices were constructed. Specifically, XC_{TD} was computed for all pairs of cortical tiles, yielding symmetric matrices analogous to a standard correlation matrix, in which each row and column corresponds to a cortical tile. The rows were then reordered by hierarchical clustering to reveal spatial groupings of tiles with similar fast-component dynamics.

We believe these revisions improve the clarity and accessibility of the Figure 4 analyses.

Reviewer #2 (Remarks to the Author):

We thank Reviewer #2 for co-reviewing this manuscript through the Communications Biology Early Career Researcher initiative, and for contributing to the peer-review process.

Reviewer #3 (Remarks to the Author):

This study uses intact-skull widefield imaging to simultaneously monitor brain blood volume (BBV), astrocytic pyruvate, and neuronal ATP, aiming to reveal energy allocation during sleep-state transitions. The attempt to integrate neurovascular coupling, astrocytic metabolism, and neuronal energy status into a unified framework is valuable. The observation during REM sleep—BBV increase and astrocytic pyruvate elevation accompanied by neuronal ATP reduction—is intriguing and potentially important. However, several issues remain unresolved. Previous studies have already shown that REM sleep is associated with vasodilation, increased glucose utilization in certain brain regions, elevated extracellular lactate, and enhanced astrocytic metabolism. Therefore, the findings of vascular dilation and increased astrocytic pyruvate during REM are not highly novel. In contrast, the observation that theta activity precedes and strongly correlates with vascular dilation shows some originality. The finding that astrocytic energy metabolism increases while neuronal ATP decreases is also notable, but the manuscript does not sufficiently explain the underlying mechanisms or biological significance.

We thank the reviewer for the careful evaluation of our manuscript and for highlighting both its strengths and the aspects requiring further clarification. We address each of the specific concerns in the point-by-point responses below.

Fig. 1: The theta–BBV coupling is interesting, but the theoretical explanation and causal interpretation remain insufficient. The authors report that theta-band ECoG power during NREM predicts BBV changes by ~4–5 seconds. However, three issues remain: 1. It is unclear why theta, rather than the more typical NREM rhythms such as delta or slow oscillations, best predicts BBV changes. Stronger theoretical justification is needed. 2. The

temporal correlation between theta and BBV does not establish causality; the data only show that neural activity precedes vascular changes. 3. The mechanism underlying the relationship between theta activity and vascular changes is not adequately explained. In particular, Fig. 7 shows that theta and BBV changes appear before alterations in pyruvate or ATP during the pre-REM phase, suggesting that theta–BBV coupling and metabolic signals are not directly or immediately linked.

Thank you for your comments on the interpretation of the theta–BBV coupling.

In our study, we found that theta-band ECoG power (6–9 Hz) precedes BBV signals by approximately 4–5 seconds and shows a strong correlation with them. When theta power was smoothed to capture its infraslow component, it closely matched BBV dynamics (r = 0.85).

1. We appreciate the reviewer's comment regarding the prominence of delta-band activity during NREM sleep. In the present study, we did not impose an a priori assumption regarding which frequency band would be most relevant to vascular dynamics. Instead, our analysis revealed that fluctuations in theta-band power, rather than those in the dominant delta band, most closely resembled BBV dynamics. We have revised the manuscript to clarify that this observation emerged from a data-driven analysis and that we currently do not have a definitive theoretical explanation for this selectivity.

2. We agree that the observed temporal relationship does not establish causality. Accordingly, we have revised the manuscript to avoid causal interpretations and to describe the relationship as a predictive or correlational association.

3. We agree that the dissociation between theta–BBV coupling and metabolic signals during the pre-REM phase indicates that these processes are not directly or immediately linked. We interpret this as suggesting that neurovascular dynamics and metabolic responses may operate on partially distinct timescales or under different regulatory mechanisms. We have revised the manuscript to clarify this point and to avoid mechanistic overinterpretation.

Taken together, we position the theta–BBV relationship as a robust empirical observation that provides a useful predictor of vascular dynamics, while its mechanistic basis remains to be established. Potential contributions of neuromodulatory systems, such as noradrenergic signaling, are discussed as a possible framework, but remain speculative in the absence of direct measurements (Discussion, page 19, paragraph 3).

Fig. 2: The finding that dYFP signals show stronger spatial correlation and fewer clusters during REM is valid. However, the analysis concerns the spatial correlation structure of BBV-related signals rather than cortical state transitions or neuronal synchrony per se. Therefore, the authors should avoid overstating the results as indicating "greater cortical functional unity" or "stronger cortical synchrony" during REM. The data only support increased spatial synchronization of BBV fluctuations.

Thank you for this important suggestion. We agree that the spatial correlation analysis of dYFP signals should be interpreted specifically in terms of BBV signal dynamics, rather than implying neuronal synchrony or cortical state coordination directly.

In the revised manuscript, we have carefully modified the relevant passages (page 10, paragraph 1; page 13, paragraph 2; Figure 4 legend, page 43) to avoid terminology such as “cortical synchrony” or “functional unity,” and instead describe the findings as reflecting increased spatial synchronization of BBV fluctuations.

These revisions ensure that the interpretation remains strictly within the scope of the measured signals and is limited to the spatial organization of BBV dynamics, without implying neuronal or cortical synchrony beyond what is directly supported by the data.

Fig. 3: The rationale for dividing signals into slow (<0.05 Hz) and fast (0.05–0.2 Hz) components is not sufficiently justified. While the separation is conceptually helpful for distinguishing global slow BBV increases from faster local fluctuations during REM, the choice of the 0.05 Hz cutoff and the 0.05–0.2 Hz fast band requires further explanation

Thank you for this helpful suggestion. We agree that the rationale for separating the dYFP signal into slow and fast components should be clarified.

In the revised manuscript (page 10, paragraph 3), we have expanded the description to explicitly state the basis for this separation. Rather than defining the cutoff purely by spectral optimization, we adopted a physiologically motivated approach. Specifically, we defined the fast component as the 0.05 to 0.2 Hz band, corresponding to the well-established frequency range of vasomotion in rodents (Sasaki et al., 2024; Davis et al., 2008). The slow component (< 0.05 Hz) was then defined as the remaining lower-frequency fluctuations, which effectively capture the large-amplitude, global BBV increase associated with the transition into REM sleep, unfolding over tens of seconds.

While alternative cutoff values could in principle be considered, this separation provides a biologically interpretable and functionally meaningful distinction between fast vasomotor dynamics and slower, state-transition-related vascular changes.

We believe this clarification strengthens the rationale for the analytical framework used in Figures 3–5.

Transition from Fig. 3 to Fig. 4: The logical progression is unclear. Fig. 3 shows a reduction in the fast component amplitude during REM, but the manuscript then directly proceeds to lag-aware spatial correlation analysis without clearly explaining why amplitude analysis alone is insufficient or what specific biological question the time-delay analysis addresses.

We thank the reviewer for this important observation. We agree that the rationale for proceeding from amplitude-based analysis to lag-aware correlation needed to be clarified.

In the revised manuscript (page 12, paragraph 1), we have added a bridging passage at the beginning of the section "Spatiotemporal dynamics of the fast component of the blood volume during NREM and REM sleep." Specifically, we now explain that although the fast component revealed distinct dynamics (Fig. 3), its amplitude was substantially reduced during REM sleep, making it difficult to directly compare spatial coordination across states

based on amplitude alone.

We further clarify that conventional zero-lag correlation analyses may underestimate inter-regional coordination when signals are temporally shifted. Therefore, to capture delayed coordination and to test whether fast BBV fluctuations exhibit structured propagation across the cortex, we employed a lag-aware cross-correlation approach.

These additions clarify both the methodological rationale and the specific biological questions addressed by the analysis.

Fig. 6: The SNP experiment shows that pharmacologically induced vasodilation increases BBV, astrocytic pyruvate, and neuronal ATP, with pyruvate rising faster than ATP. This supports the idea that increased BBV can be accompanied by changes in energy-related signals. However, the authors' interpretation is too strong. The data do not demonstrate a causal chain (BBV increase → astrocytic pyruvate increase → neuronal ATP increase), particularly because pyruvate and ATP measurements were obtained from different groups of mice and SNP may have effects beyond simply increasing BBV.

We thank the reviewer for this important comment. In our study, astrocytic pyruvate and neuronal ATP were measured in separate transgenic mouse lines, and therefore represent distinct cellular compartments.

Based on these data, we report parallel increases in astrocytic pyruvate and neuronal ATP following SNP-induced vasodilation. As noted by the reviewer, these measurements were obtained in different animals and do not establish a causal chain between these variables. We also acknowledge that SNP may have effects beyond vasodilation.

We have revised the manuscript accordingly to avoid implying a sequential causal relationship and instead describe the observed association (page 16, paragraph 2).

Fig. 7: This figure suggests that vascular changes do not immediately induce changes in

pyruvate or ATP. During the pre-REM phase, both theta power and BBV increase before REM onset, but astrocytic pyruvate and neuronal ATP do not change correspondingly. This indicates a temporal dissociation between neurovascular changes and metabolic signals. Given that SNP-induced BBV increases (Fig. 6) are associated with metabolic changes, the authors should discuss why vascular changes during natural sleep transitions produce different metabolic consequences compared with pharmacological vasodilation.

Thank you for this insightful comment. We appreciate the reviewer pointing out that the apparent discrepancy between the pre-REM period and the SNP experiment warrants explicit discussion.

One possible explanation is that the BBV increase during the pre-REM period occurs alongside a concurrent rise in neuronal activity, whereas SNP-induced vasodilation does not involve a comparable increase in neuronal activity. This difference may influence how changes in substrate delivery are reflected in intracellular metabolite signals.

We have expanded the relevant Discussion paragraphs (pages 20, paragraph 3) to address this point and to highlight that these interpretations remain speculative. We believe this addition provides a possible framework for understanding the different metabolic outcomes observed under these two conditions.

Fig. 8: The core observation—BBV increase and astrocytic pyruvate elevation accompanied by neuronal ATP reduction during REM, especially in posterior cortex (RSC/V1)—is novel and important. However, the mechanistic interpretation appears premature. 1. The explanation of astrocytic pyruvate as a direct reflection of glucose supply is overly simplistic. Pyruvate levels can also be influenced by glycogen mobilization, lactate/pyruvate transport, and mitochondrial consumption. 2. Although the "BBV↑ / pyruvate↑ / ATP↓" pattern is robust, the proposed mechanisms (increased ATP consumption, substrate transport limitations, altered mitochondrial metabolism) remain speculative and require further experimental support.

Thank you for your comment regarding the mechanisms underlying the observed changes

in intracellular metabolite levels.

In this study, we focused on the relationship between brain blood volume (BBV), a key determinant of substrate delivery, and intracellular metabolite signals. Our data show that increases in BBV are associated with increases in astrocytic pyruvate, both during pharmacological vasodilation and REM sleep.

We agree that astrocytic pyruvate levels are influenced by multiple factors, including substrate delivery, glycolytic activity, metabolite transport, and mitochondrial utilization. In the revised manuscript, we have clarified that astrocytic pyruvate should not be interpreted as a direct or exclusive reflection of glucose supply.

We also acknowledge that the mechanisms underlying the observed "BBV↑ / pyruvate↑ / ATP↓" pattern remain speculative. Our intention was not to establish a specific mechanism, but rather to outline several plausible, nonexclusive explanations consistent with the data. We have revised the text to more clearly distinguish between observed findings and proposed interpretations.

Nonetheless, we believe that the core observation—that astrocytic pyruvate increases while neuronal ATP decreases during REM sleep, with clear regional specificity—represents a novel and important contribution to understanding brain energy metabolism.

Reviewer #4 (Remarks to the Author):

This is a nice study about the relation between brain state (NREM/REM sleep and wakefulness), hyperemia, and metabolite levels. The experimental procedures appears to be well-conducted, although not necessarily well-designed for the goal of the study (see below). However, I have some concerns about both authors' assumptions on measured quantities and, accordingly, interpretation of the results. These are described below. I have found the paper interesting, but quite unbalanced. Specifically, there is a lot of space and details devoted to the analysis of the spatio-temporal cortical fluorescence patterns, and then a too simplistic treatment of the metabolite data, which is forced to biochemical interpretations that are not entirely supported by the measurements. Overall, the finding that activity-CBF in sleep are characterized by an anterior-posterior axis is not entirely new (Wang et al., 2022; Stephan et al., 2025). Similarly, the rise in pyruvate during REM sleep is expected as it has been repeatedly observed that lactate drops during NREM sleep compared to either REM or wakefulness (Naylor et al., 2012; Clegern et al., 2012; Shram et al., 2002; Rempe et al., 2014; Dash et al., 2012; Cespuglio et al., 2013). The ATP finding is more interesting, although expected on the basis of previous studies (Dworak et al., 2010). The main message of the paper, i.e., assigning metabolite dynamics to either neurons or astrocytes, may suffer of sub-optimal study design (see below). This is unfortunate, because the study has clearly been effortful, and the quality of data (figures, videos) is high. My suggestion would be to remove the metabolic interpretations and slightly reframe the presentation of the study goal/findings.

We thank the reviewer for the careful and detailed evaluation of our manuscript. We have refined the framing of the metabolite findings throughout the manuscript to more closely align the interpretations with the scope of our measurements, and we address the specific concerns in the point-by-point responses below.

Comment 1 (Page 3: NKA)

While introducing ionic homeostasis and Na/K-ATPase (NKA) activity, please mention that not only neuronal NKA is stimulated during neuronal activity, but also astrocytic NKA. Astrocytes are though to account for at least 1/3 of extracellular K+ reuptake, and

correspondingly 20-25% of cortical energy expenditure.

We thank the reviewer for this insightful suggestion. We agree that astrocytes are not merely energy suppliers but also active contributors to energy consumption through their own physiological processes.

In the revised manuscript (page 3, paragraph 3), we have added a description noting that astrocytes express Na^+/K^+-ATPase and play a major role in the reuptake of extracellular K^+ released during neuronal activity. We also revised the subsequent sentence to refer to Na^+/K^+-ATPase activity in both neurons and astrocytes.

In addition, we now explicitly acknowledge the substantial energetic cost associated with astrocytic activity, citing Barros (2022; J. Cereb. Blood Flow Metab. 42, 738–745), to better reflect the contribution of astrocytes to overall cortical energy expenditure.

Comment 2 (Pages 3–4, lines 67–81: ANLS / lactate shuttle)
While it is true that pyramidal neurons (as opposed to interneurons) do not directly contact the vasculature, this does not mean that neurons rely on astrocyte-derived lactate (e.g., astrocyte-neuron lactate shuttle). This has been quite debated in the field, so I'm not citing the whole literature. Briefly, Nehlig et al. (2003) demonstrated that neurons take up >50% of blood-borne glucose. Simpson et al. (2007) substantiated such observation by mathematical modeling of GLUT transporter proteins. To name just a few follow-up studies, confirmations of direct neuronal glucose uptake can be found in Patel et al. (2014), Lundgaard et al. (2015), and more recently in Li et al. (2023). Overall, as discussed repeatedly by Leif Hertz, Gerald Dienel, and Douglas Rothman (to name some of the most prominent scientists in the field) both neurons and astrocytes can take up glucose and both can release lactate under specific circumstances, possibly depending on brain state. Thus, the argument that astrocytic pyruvate utilization reduces availability of lactate for neurons is rather weak. Besides, astrocytes mobilize glycogen during high activity (in a K+ uptake-related manner), which can be another source of pyruvate for either oxidation or export. It has been further demonstrated by Gandhi et al. (2009), Ball et al. (2010), and Lundgaard et al. (2017) that intracerebrally produced lactate predominantly leaves the brain via perivascular/lymphatic routes. These

clearance pathways are thought to be dependent on the sleep-wake cycle, further complicating the picture

Thank you for this important and detailed comment. We fully agree that the metabolic interplay between neurons and astrocytes is highly complex and cannot be adequately explained by a single pathway.

In the revised Introduction (page 4, paragraphs 2–5), we have expanded the discussion to provide a more balanced view. In addition to the astrocyte–neuron lactate shuttle (ANLS) hypothesis, we now explicitly acknowledge substantial evidence for direct neuronal glucose uptake, citing Nehlig et al. (2003), Simpson et al. (2007), and Lundgaard et al. (2015). We also emphasize that both neurons and astrocytes can utilize multiple metabolic pathways, and that their relative contributions may vary depending on brain state.

Accordingly, we have revised our description to avoid implying that neuronal energy supply is primarily dependent on astrocyte-derived lactate. Instead, we now frame changes in astrocytic pyruvate levels as reflecting alterations in metabolic state that may influence, but do not solely determine, substrate availability for neurons.

Following the reviewer's suggestion, we have also added a description of astrocytic glycogen mobilization linked to K^+ uptake, which can provide an additional source of pyruvate for oxidation or export.

These revisions ensure that our interpretation does not rely on a specific metabolic model and better reflects the current consensus that multiple, parallel pathways contribute to energy metabolism in the brain.

Comment 3 (Page 4, lines 84–94: ATP buffering)

Cellular ATP levels are only minimally influenced by energy demand because ATP is buffered by creatine kinase and adenylate kinase (among others), and rapidly replenished by glycolysis and oxidative phosphorylation. As the authors states, it is very difficult to measure changes in ATP.

We thank the reviewer for this important comment regarding ATP buffering and the challenges of detecting ATP changes *in vivo*.

We agree that cytosolic ATP levels are generally tightly regulated by buffering systems such as creatine kinase and adenylate kinase, as well as by rapid metabolic compensation. Under most physiological conditions, these mechanisms maintain ATP levels within a narrow range.

However, previous studies have shown that measurable decreases in neuronal ATP can occur under conditions of substantial metabolic demand or imbalance, such as during hyperexcitation or pathological states. These observations indicate that ATP levels, while generally stable, can deviate from baseline under specific conditions.

In the present study, we used a FRET-based ATP sensor (ATeam) and applied a correction method using the ATP-insensitive dYFP signal to minimize contributions from hemodynamic and pH-related artifacts. As detailed in Supplementary Fig. 6, this approach enables extraction of ATP-related signals under *in vivo* conditions.

Importantly, when vasodilation was induced pharmacologically by SNP injection, the calculated ATP signal increased, in contrast to the decrease observed during REM sleep. This differential response indicates that the extracted ATP signal does not simply reflect vascular or optical artifacts, but instead captures condition-specific biological changes.

Taken together, while ATP levels are generally buffered, our results suggest that the REM-associated decrease in neuronal ATP reflects a deviation from this buffered state, rather than a measurement artifact.

Comment 4 (Page 4, lines 109–110: pyruvate ascription)
Again, the observation that pyruvate increases following BBV surge cannot be ascribed to astrocytes only.

We thank the reviewer for this important point. We agree that the increase in pyruvate

observed with the astrocyte-expressed sensor reflects changes in intracellular pyruvate levels within astrocytes, but does not specify the cellular or metabolic origin of the substrate.

We also acknowledge that similar metabolic changes may occur in other cellular compartments, including neurons, and that our measurements do not allow direct comparison across cell types. Accordingly, we have revised the text to avoid attributing the observed increase specifically to astrocytic production, and instead describe it as a change in astrocytic pyruvate levels.

We further note that such changes may arise from multiple processes, including glycolysis, glycogen metabolism, or intercellular substrate exchange.

The relevant passage has been revised accordingly (page 6, paragraph 2).

Comment 5 (Page 4, lines 114–118: exclusion of options)
The authors are right in excluding options (1) and (2). REM sleep is as expensive as active waking, i.e., more expensive than quiet waking. Thus, it is expected that energy demand rises during the transition from NREM to REM sleep.

Thank you for this supportive comment.

We agree that an increase in energy demand during REM sleep is a plausible explanation for the observed decrease in neuronal ATP. However, based on our current data, we do not consider it possible to distinguish among the three proposed mechanisms, namely changes in energy supply, production, or consumption.

While increased consumption is a commonly assumed explanation, we note that our data do not provide direct evidence that ATP demand rises to a level sufficient to account for the observed decrease. Conversely, mechanisms involving limitations in energy supply or production cannot be excluded and may contribute to the observed dynamics.

We have therefore revised the manuscript to more clearly emphasize that these possibilities

remain open and that the ATP decrease may reflect a complex interplay between supply, production, and consumption under REM-specific metabolic conditions.

Comment 6 (Page 7, lines 169–172 & Page 13, lines 336–337: pH effects)
What support is given to exclude substantial pH effects? What is the range of pH affecting each fluorescence signal? This is relevant especially because the authors discuss the results suggesting an involvement of astrocytic acidification (Discussion, Page 19 lines 494–503)

Thank you for raising this important point regarding the pH sensitivity of the fluorescence signals.

We agree that pH can influence fluorescence-based measurements and have taken several steps to minimize its impact. The ATP sensor ATeam1.03YEMK has been characterized to show minimal changes in the CFP/YFP ratio within the physiological pH range (approximately pH 7.0 to 7.4), while more pronounced effects occur under acidic conditions (e.g., pH 6.8) (Imamura et al., 2009).

To account for potential pH and hemodynamic influences, we used the directly excited YFP signal (dYFP) as an internal reference and subtracted it from the FRET-derived YFP signal (fYFP) after appropriate scaling. Because both signals originate from the same YFP fluorophore, shared effects such as pH fluctuations are largely cancelled in the resulting metabolite signal.

Consistent with this interpretation, dYFP signals were strongly anticorrelated with independently measured vascular signals (mScarlet) across sleep states ($n = 14$; NREM, $r = -0.901$; REM, $r = -0.957$), indicating that dYFP primarily reflects BBV-related changes, with any pH contribution being either small or tightly coupled to vascular dynamics.

While we cannot completely exclude the possibility of residual pH effects, these are unlikely to account for the observed metabolite signals. Importantly, our previous work has shown that astrocytes undergo acidification during REM sleep (Ikoma et al., 2023), suggesting that

physiologically meaningful pH changes may coexist with, but do not dominate, the extracted metabolite signals.

Comment 7 (Page 8, lines 199–201: theta-band ECoG and literature)
How the finding of theta-band ECoG relates to literature (Hillman??)

Thank you for this comment regarding the relationship between our theta-band ECoG findings and the existing literature.

In our study, theta-band ECoG power (6 to 9 Hz) precedes BBV signals by approximately 4 to 5 seconds and shows a strong correlation with them. When theta power was smoothed to capture its infraslow component, it closely matched BBV dynamics.

These findings are consistent with the general framework of neurovascular coupling, in which neuronal activity is temporally linked to subsequent hemodynamic responses (e.g., Hillman, 2014). In this context, our results extend previous work by identifying theta-band fluctuations as a particularly strong predictor of BBV dynamics during NREM sleep.

Previous studies have also reported that infraslow fluctuations in cortical activity are coupled to vascular signals and modulated by neuromodulatory systems such as noradrenaline (NA; or norepinephrine, NE) (Kjaerby et al., 2022; Teng et al., 2025; Hauglund et al., 2025). While these findings provide a potential framework, the contribution of such mechanisms to the theta–BBV relationship observed in our study remains speculative, as neuromodulatory signals were not directly measured.

We have added a discussion of these points in the revised manuscript (page 20, paragraph 4).

Comment 8 (Page 9, lines 214–216: anterior-posterior waves under anesthesia)
Large-scale anterior-posterior waves are also present during anesthesia (e.g., ketamine-xylazine). It'd have been interesting to compare the patterns in NREM sleep and anesthesia.

Did the authors have some observations during KX or other anesthetics?

Thank you for this interesting suggestion. We agree that comparing BBV wave patterns between natural NREM sleep and anesthesia would be highly informative. However, as our study focused on BBV dynamics under physiological sleep conditions, we did not acquire data under anesthesia.

Previous studies have reported large-scale anterior to posterior propagation patterns under anesthesia. For example, Mitra et al. (2018) characterized spatiotemporal trajectories of infra-slow and delta-band cortical activity under ketamine and dexmedetomidine anesthesia, demonstrating state-dependent directional wave propagation. This point has now been introduced in the revised manuscript (page 19, paragraph 4).

In this context, the anterior to posterior BBV dynamics observed in our study may reflect a broader organizing principle of large-scale cortical dynamics that manifests across different brain states, including both sleep and anesthesia. A direct comparison between these conditions would be an important direction for future work.

Comment 9 (Page 12, lines 323–324: "prepare the metabolic landscape")
This statement ("prepare the metabolic landscape for REM sleep") is too speculative. Please remove.

Thank you for this comment. Our intention was to highlight the temporal association between the progressive increase in posterior cortical BBV motifs during late NREM and the subsequent transition to REM sleep. However, we acknowledge that the phrase “prepare the metabolic landscape” unintentionally implies a functional or causal role that is not directly supported by our data.

We have therefore revised the statement to describe the observation in purely temporal terms, indicating that posterior cortical BBV dynamics precede and are temporally aligned with the transition to REM sleep (page 15, paragraph 1), without implying a mechanistic role.

Comment 10 (Page 12, line 326; Page 13, line 329; Page 15, line 389; and possibly more: "energy molecule")

Please avoid using ambiguous terminology, as "energy molecule" is not appropriate, and highly misleading for pyruvate. Specifically, metabolite levels (pyruvate and ATP) cannot be used as proxy of metabolic rates. Indeed, a decrease might indicate reduced production, increased consumption, reduced uptake, increased clearance/washout, or any combination thereof. Since "energy metabolism" is related to metabolic rates, it is not appropriate to state that this study assessed energy dynamics, also considering the complexities of intracellular ATP buffering system and the intercellular lactate trafficking mentioned above.

We thank the reviewer for this important terminological comment, which has helped us refine the precision of our discussion. As the reviewer points out, pyruvate is not solely involved in energy production, and we agree that the term “energy molecule” is not appropriate for describing it. We have therefore replaced “energy molecule” with “metabolite” when referring to pyruvate and ATP (page 15 paragraph 2; page 17, paragraph 4; page 18, paragraph 5).

We also agree that the metabolite levels measured in this study represent integrated outcomes of multiple processes, including production, consumption, cellular uptake, and clearance, and therefore do not directly report metabolic rates. Accordingly, we have revised our terminology to avoid expressions such as “energy metabolism” or “energy dynamics” where they may imply metabolic flux or detailed mechanistic interpretation, and instead refer explicitly to changes in intracellular metabolite levels (page 19, paragraph 1).

These revisions ensure that our interpretation is strictly limited to the measured quantities and does not imply metabolic rates or fluxes beyond what is supported by the data.

Comment 11 (Page 13, lines 334–335 & Page 14, lines 361–366: cell-type specificity of metabolite sensors)

If I understand correctly, the pyruvate sensor was only expressed in astrocytes, and the ATP sensor was only expressed in neurons. If that's the case, the finding (described on Page 14 lines 361–366) that pyruvate increases and ATP decreases upon vasodilation cannot be

interpreted in a cellular-centered manner. Very likely pyruvate is increasing in all tissue compartments (extracellular, neurons, astrocytes) and possibly the same for ATP (neuronal and astrocytic). Since pyruvate and ATP are estimated only in one cellular compartment, the authors' conclusion that "blood volume enhances the energy supply, first boosting astrocytic pyruvate levels, which is then followed by an increase in neuronal ATP production" remains speculative at best. I understand that this FRET experiments are hard to carry out, but a good study design would have attempted measuring the same metabolite in different cell types (of course in different animals, which is fine)

We thank the reviewer for highlighting the importance of cell-type specificity in the interpretation of these measurements.

In our study, pyruvate and ATP were measured in astrocytes and neurons, respectively, and therefore reflect metabolite dynamics in distinct cellular compartments. We agree that these measurements, obtained in separate cell types and in different animals, do not allow us to establish a direct causal or sequential relationship between astrocytic and neuronal metabolic processes.

Accordingly, we have revised the manuscript to avoid implying a direct or causal sequential relationship between astrocytic and neuronal metabolic processes, and to clearly distinguish between observed temporal associations and inferred mechanisms (page 16, paragraph 2).

Importantly, while the present data do not resolve how metabolites are distributed across cellular compartments, they reveal that vascular changes are accompanied by parallel but non-identical metabolic responses in astrocytes and neurons. This observation provides a basis for future studies aimed at directly comparing metabolite dynamics across cell types.

Comment 12 (Page 14, lines 370–371: "preparatory" & neuromodulators)
I'm not sure "preparatory" is appropriate here. Please notice that sleep stages/substages are not homogeneous in terms of neuromodulators NE and ACh. These neuromodulators significantly influence baseline CBF, with NE typically leading to vasoconstriction and ACh vasodilation. Transition periods between NREM and REM are associated with progressive

reduction of NE and increase of ACh. Interestingly, neuromodulators (NE and ACh, but also 5-HT) are released in a synchronized fashion during NREM sleep. In other words, their release is coordinated spatio-temporally (Teng et al., 2025), which might well be related to the "wave"-like hyperemia observed in this and other studies.

We thank the reviewer for this valuable insight into the relationship between BBV signals and neuromodulatory dynamics. We agree that the term “preparatory” may imply a teleological or causal role of BBV increases in initiating REM sleep, which is not directly supported by our data.

Accordingly, we have revised the manuscript to avoid this terminology and instead describe these BBV changes in purely temporal terms, without implying a functional or causal role (page 16, paragraph 3; page 17, paragraph 3).

In light of the reviewer’s comment, we further consider that the observed BBV dynamics may reflect state-dependent changes in neuromodulatory tone. For example, previous studies have shown that locus coeruleus activity and noradrenaline (NA; or norepinephrine, NE) levels decline prior to REM onset (Kjaerby et al., 2022), while other neuromodulators such as acetylcholine (ACh) increase. In addition, coordinated spatiotemporal dynamics of NA, ACh, and serotonin (5-HT) during sleep have been reported (Teng et al., 2025), providing a plausible framework for the wave-like BBV fluctuations observed in our study.

We have added a discussion of these neuromodulatory contributions as a potential, but not directly tested, mechanism underlying pre-REM hemodynamic changes (page 21, paragraph 2).

Comment 13 (Page 17, lines 424–425: known finding)
The first sentence states something that is already well known.

We thank the reviewer for this comment. Our intention in this sentence was not to restate a general concept, but to highlight a key observation of this study: that metabolic signals do not simply scale with neuronal activity, but can diverge from it in a state-dependent manner.

We have revised the sentence to clarify this point more explicitly and to better reflect the specific findings of our data (Discussion, page 19, paragraph 1; page 21, paragraph 4).

Comment 14 (Page 18, lines 452–455: astrocytes driving state transitions)
The possibility that astrocytes may drive state transitions is interesting and it has been proposed long ago. However, there is no result in the present study to support that claim.

We thank the reviewer for this comment. We acknowledge that the present study does not provide direct evidence that astrocytes drive sleep-state transitions.

In light of this, we have revised the manuscript to avoid such interpretations. While our data show that changes in astrocytic and vascular signals precede changes in neuronal activity, these observations indicate a temporal relationship but do not establish a causal contribution to neuronal state transitions.

Accordingly, we have softened the original phrasing, replacing “astrocytes and vasculature may also actively modulate, or even drive, neuronal network states” with “astrocytes and the vasculature may not only respond passively to neuronal activity but may also exhibit dynamics that precede changes in neuronal network states” (page 20, paragraph 2).

Comment 15 (Page 18, lines 466–468: pyruvate increase = supply vs. demand; "paradox")
Literature strongly indicates that the increase in pyruvate (and lactate) during REM sleep does not reflect increased supply (as the authors suggest), but a rise in metabolic rate (mainly glycolysis) in the transition from NREM to REM. This consideration makes the subsequent statement less "paradoxical" (in authors' words: "Paradoxically, despite this increased supply, neuronal cytosolic ATP levels markedly decreased"). That's because it is not increased supply but increased demand, as the authors themselves subsequently discuss, correctly suggesting other sources of energy expenditure in addition to NKA (e.g., plasticity). In addition to characterize the study being in the title ("The paradox in REM sleep"), the same wording ("paradoxical") appears on Page 20 line 522, which the authors relate to fMRI. I don't understand the argument, because increased CBF and CBV during

stimulation coexist with increased energy demand and small but significant ATP reductions, all of which is physiological.

We thank the reviewer for this important comment and for pointing out the established view that increases in pyruvate and lactate during REM sleep are closely linked to elevated metabolic demand.

We would like to clarify that our use of the term “paradox” was not intended to imply that metabolite levels are determined solely by vascular supply. Rather, we introduced a simplified hypothetical scenario—namely, that if metabolite levels were primarily governed by substrate delivery, then increases in BBV would be expected to elevate both pyruvate and ATP levels.

While an increase in astrocytic pyruvate during REM sleep is consistent with this expectation, the observed decrease in neuronal ATP indicates that this supply-driven framework alone is insufficient to explain the data. We therefore interpret this dissociation as evidence that additional factors beyond substrate delivery, including increased metabolic demand, must be taken into account.

In the revised manuscript, we have clarified this logical structure and revised the wording to avoid implying that metabolite changes are determined solely by vascular supply (Discussion, page 21, paragraph 4).

TOHOKU UNIVERSITY

Tohoku University, Graduate School of Life Sciences

Super-network Brain Physiology
2-1-1 Katahira, Aoba-ku, Sendai 980-8577 JAPAN
Phone: +81-22-217-6209, Fax: +81-22-217-6211
E-mail: matsui@ tohoku.ac.jp
http://www.ims.med.tohoku.ac.jp/matsui/

Manuscript ID: COMMSBIO-26-1065-T
Title: Energy paradox in REM sleep: balancing supply and consumption in brain metabolism
Corresponding author: Ko Matsui

Point-to-point rebuttal to the referees' comments.

We are grateful to the reviewers for their continued and careful evaluation of our manuscript, and we are pleased that they find their previous concerns to have been addressed. We appreciate the reviewers' positive overall assessment of our study. The original comments are highlighted in blue, while our responses are provided in black.

Reviewer #1 (Remarks to the Author):

The authors have addressed the majority of my concerns. Although it still takes time to understand the whole logic behind the calculation, the text reads much more reader-friendly.

We thank the reviewer for the positive assessment and for noting that the manuscript now reads in a more reader-friendly manner. By addressing the reviewer's comments on the calculation of the fluorescence signal and on the overall flow and detail of the analysis, we were able to make our study easier to follow. We are grateful for the reviewer's constructive engagement throughout the review process, which has meaningfully improved the clarity of the manuscript.

Reviewer #2 (Remarks to the Author):

I co-reviewed this manuscript with one of the reviewers who provided the listed reports. This is part of the Communications Biology initiative to facilitate training in peer review and to provide appropriate recognition for Early Career Researchers who co-review manuscripts.

We thank the Reviewer #2 for co-reviewing our manuscript as part of the Communications Biology initiative, and we appreciate their contribution to the evaluation of our study.

Reviewer #3 (Remarks to the Author):

I have no further comments.

We thank the reviewer for the constructive engagement with our manuscript throughout the review process. Following the reviewer's comments, we were able to add a more concrete discussion of the coupling between theta-band activity and brain blood volume. We were also able to make the overall flow of the analysis easier for readers to follow, and to refine the interpretation of our results more appropriately. We deeply appreciate the insight the reviewer has brought to evaluating our study.

Additionally, I carefully reviewed the authors' rebuttal and responses to Reviewer #4's concerns. Overall, I think the authors have addressed many of the major conceptual criticisms in a constructive and scientifically appropriate manner. In particular, the authors substantially revised the framing of the metabolic findings and now more carefully distinguish between measured observations and mechanistic interpretation. Several points raised by Reviewer #4 were appropriately addressed, including:

We thank Reviewer #3 for taking the time to carefully review our rebuttal and our responses to Reviewer #4's concerns. We are grateful that the reviewer considers the major conceptual criticisms to have been addressed in a constructive and scientifically appropriate manner,

and that the reviewer recognizes our revised framing as more carefully distinguishing our measured observations from their mechanistic interpretation.

We thank Reviewer #4 for the detailed and insightful comments provided during the earlier rounds of review. Engaging with these comments prompted us to substantially revise the framing of our metabolic findings and to distinguish more carefully between our measured observations and their mechanistic interpretation. We appreciate the rigor and interpretational restraint that the reviewer's feedback has brought to the manuscript.

clarification that pyruvate and ATP measurements reflect intracellular metabolite levels rather than direct measurements of metabolic flux or "energy dynamics"; removal or softening of overly causal or teleological language such as "preparatory" and "energy molecule"; clearer acknowledgment that pyruvate and ATP were measured in different cellular compartments and different animals, limiting cell-type-specific mechanistic conclusions; broader discussion of alternative metabolic frameworks, including direct neuronal glucose uptake, astrocytic glycogen metabolism, and neuromodulatory influences; improved discussion of pH/hemodynamic confounds and internal correction strategies; reduction of strong astrocyte-centered interpretations and causal claims. Importantly, the authors now present the study in a more balanced way, emphasizing that the work primarily identifies state-dependent associations among vascular dynamics and intracellular metabolite signals rather than establishing a definitive mechanistic pathway.

I also think the authors appropriately acknowledged the limitations of their experimental design, especially regarding the inability to infer causal or sequential relationships between astrocytic pyruvate and neuronal ATP dynamics.

We appreciate the reviewer's careful and accurate summary of the revisions made in response to these points. Through these revisions, we were able to make the interpretation of our data more accurate and balanced.

That said, some conceptual limitations naturally remain unresolved. In particular, the study still cannot fully distinguish whether the observed metabolite changes primarily reflect altered supply, consumption, transport, buffering, or clearance processes. Similarly, the use of the term "paradox" may still be somewhat interpretive, although the revised manuscript now defines this more carefully as a dissociation between vascular/metabolite-related signals rather than a contradiction to known physiology.

Overall, however, I think the authors have responded to Reviewer #4's concerns in good faith and substantially improved the rigor and interpretational restraint of the manuscript. While some mechanistic questions remain open, I do not believe the remaining issues fundamentally preclude publication, provided the revised conservative framing is maintained throughout the manuscript.

As the reviewer notes, the detailed mechanisms underlying the dissociation between vascular and metabolite-related signals that we identified here remain to be fully elucidated. We are grateful for the reviewer's overall assessment, and we have taken care to maintain this conservative framing throughout the manuscript.
